# Subnuclear genome compartmentalization controls bivalent chromatin activity

**DOI:** 10.1038/s41586-026-10832-w

**Published:** 2026-07-22

**Authors:** Sajad Hamid Ahanger, Evan R. Semenza, Chujing Zhang, Eugene Gil, Mitchel A. Cole, Serena Huei-An Lu, Li Wang, Arnold R. Kriegstein, Daniel A. Lim

**Affiliations:** 1 Department of Neurological Surgery, University of California, San Francisco, San Francisco, CA USA; 2 Eli and Edythe Broad Center of Regeneration Medicine and Stem Cell Research, University of California, San Francisco, San Francisco, CA USA; 3 Biomedical Sciences Graduate Program, University of California, San Francisco, San Francisco, CA USA; 4 Medical Scientist Training Program, University of California, San Francisco, San Francisco, CA USA; 5 Department of Neurology, University of California, San Francisco, San Francisco, CA USA; 6 San Francisco Veterans Affairs Health Care System, San Francisco, CA USA

**Keywords:** Epigenetics in the nervous system, Nuclear envelope

## Abstract

The nuclear genome is spatially organized into a three-dimensional architecture by physical association of large chromosomal domains with subnuclear compartments including the nuclear lamina at the radial periphery and nuclear speckles within the nucleoplasm^1–5^. However, how higher-order spatial genome architecture regulates human development has been overlooked, and the interplay between chromatin state and subnuclear genome compartmentalization is poorly understood. Here we generate high-resolution maps of genomic interactions with the lamina and speckles in cells of the neurogenic lineage isolated from mid-gestational human cortex, identifying an intimate association between subnuclear genome compartmentalization, chromatin state and transcription. During cortical neurogenesis, subnuclear genome compartmentalization is extensively remodelled, relocating hundreds of neuronal genes from the lamina to speckles, including key neurodevelopmental genes bivalent for trimethylation of histone H3 at Lys27 (H3K27me3) and Lys4 (H3K4me3). At the lamina, bivalent genes have exceptionally low expression, and relocation to speckles enhances resolution of bivalent chromatin to H3K4me3 monovalency and increases transcription more than eightfold. We further demonstrate that proximity to the nuclear periphery—not the presence of H3K27me3—maintains the lowly expressed, poised state of bivalent genes embedded in the lamina. We find that the repressive environment of the lamina is associated with spatial segregation of the transcriptional elongation machinery from the nuclear periphery. Our results establish a paradigm in which knowing the spatial location of a gene is necessary for understanding its epigenomic regulation.

## Abstract (web summary)

Knowledge of the spatial location of a gene is necessary to understand its epigenomic regulation.

## Main

The nuclear genome is spatially organized through interactions with distinct subnuclear compartments including the nuclear lamina and nuclear speckles^1–5^. The nuclear lamina lies underneath the inner nuclear membrane and comprises lamin intermediate filaments and associated proteins^6^. Around 30–40% of the human genome is anchored to the lamina through variably sized (10 kb to 10 Mb) lamina-associated domains (LADs) containing lowly expressed genes^7–10^. Interior to the lamina, nuclear speckles are punctate, membraneless compartments enriched in transcriptional regulators and RNA splicing factors^11–13^. Around 10–15% of the genome is organized around speckles through speckle-associated domains (SPADs) containing highly expressed genes^7,14,15^. The organization and dynamics of LADs and SPADs in developing human tissues in vivo are unclear. Moreover, the functional interplay between this spatial genome organization and other epigenomic features is poorly understood.

Chromatin modifications represent a key layer of developmental transcriptional regulation^16^. H3K4me3 at gene promoters is associated with transcriptional activation, whereas H3K27me3 marks repressed genes^17^. The identification of bivalent chromatin, characterized by local enrichment of both H3K4me3 and H3K27me3, established a paradigm in which developmental genes are maintained in a poised but repressed state in undifferentiated cells^18,19^. Resolution of bivalency to either the H3K4me3 or H3K27me3-monovalent state is associated with gene activation or repression, respectively, during cell differentiation^19–22^. However, bivalency resolution to H3K4me3 monovalency does not invariably result in transcriptional induction^20,23^, indicating that additional regulatory mechanisms shape the transcriptional output of bivalent domains.

Most of our understanding of LAD and SPAD organization comes from cell culture, owing to both technical challenges and the difficulty of performing such experiments in vivo. In this study, we developed methods to profile a specific neurogenic cellular lineage acutely isolated from mid-gestational human cerebral cortex, enabling in vivo analysis of spatial genome organization dynamics and their interplay with local chromatin state during neurogenesis. Our analyses established subnuclear genome compartmentalization as a key regulator of bivalency. Functional experiments demonstrated that the lamina represses transcription independently of local H3K27me3. Furthermore, we found that key cofactors of RNA polymerase II are spatially excluded from the lamina, providing insights into how subnuclear location contributes to the poised state of genes marked by bivalency. Together, these findings establish the interplay between spatial segregation of genomic loci and local chromatin state as a fundamental aspect of gene expression programs.

## Global LAD remodelling during neurogenesis

**Extended Data Fig. 1:**
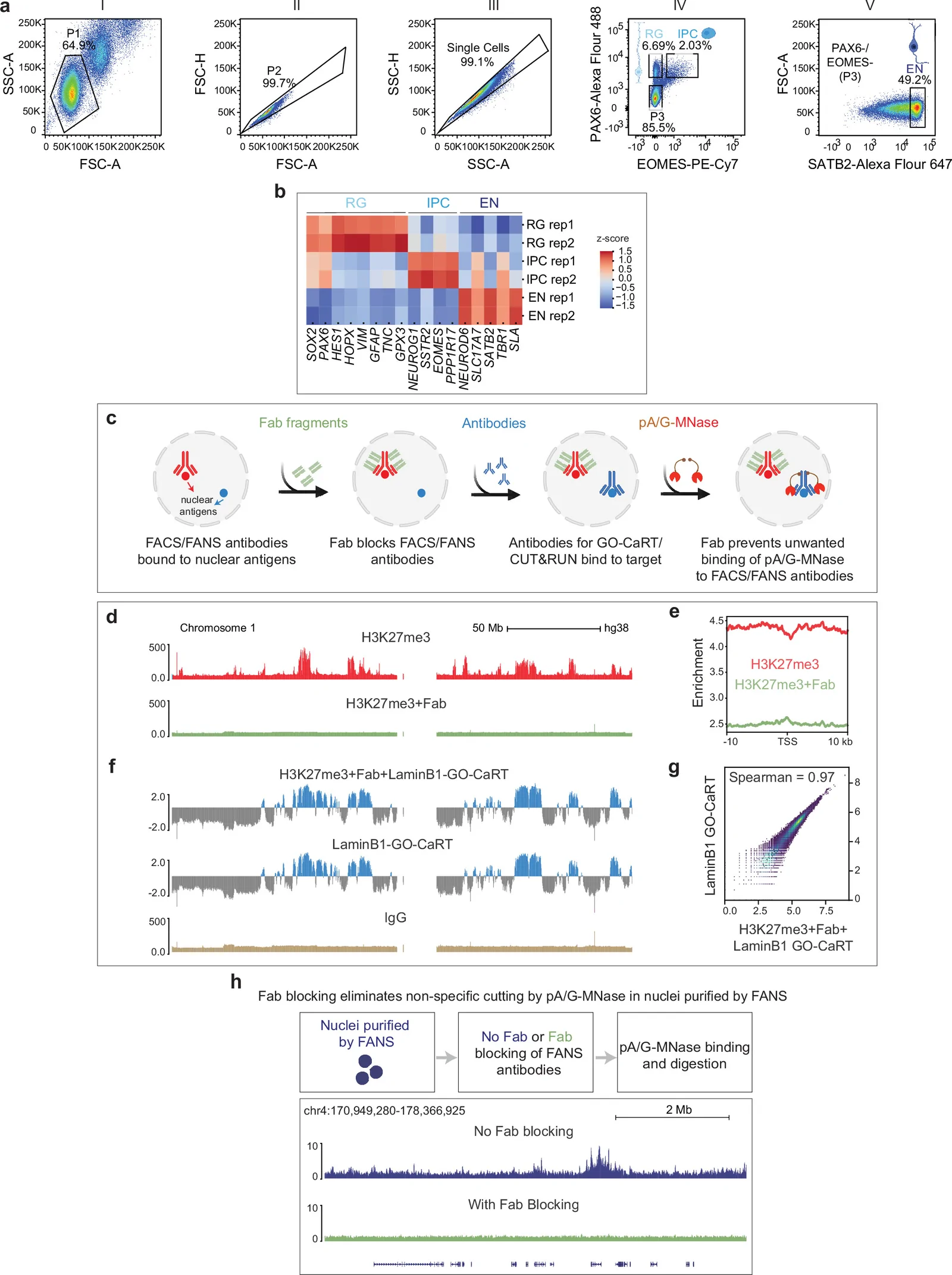
Flow cytometry gating strategy for isolation of RG, IPC and EN and development of Fab blocking for GO-CaRT. **a**, Representative gating strategy for FANS-based isolation of RG, IPC and EN from mid-gestational human brain cortex. Nuclei were separated from debris by forward scatter area (FSC-A) versus side scatter area (SSC-A) (P1, plot I). Single cells (singlets) were then selected using two gates based on area versus height of the forward scatter (FSC-A vs. FSC-H, plot II) and side scatter (SSC-A vs. SSC-H, plot III). RG populations were isolated by gating on PAX6-Alexa Fluor 488 staining and IPC by gating on PAX6-Alexa Fluor 488 and EOMES-PE-Cy7 staining (plot IV). EN were isolated from PAX6/EOMES negative population (P3) by gating on SATB2-Alexa Fluor 647 staining (plot V). **b**, Heatmap showing the expression (row normalized) of key marker genes in RG, IPC and EN isolated by FANS from GW20 cortex. R1 and R2 indicate two replicates. **c**, Schematic of Fab blocking method. Antibodies used in FANS are blocked by monovalent Fab fragments. In subsequent steps, samples are incubated with antibodies of interest for GO-CaRT and CUT&RUN. The use of Fab blocking prevents interactions between pA/G-MNase, and antibodies used in FANS allowing seamless downstream GO-CaRT and CUT&RUN analyses. **d**, Representative genome browser tracks demonstrating the efficacy of Fab fragments for blocking interaction between pA/G-MNase and antibodies to H3K27me3 in CUT&RUN assay in HEK293T cells. Without Fab treatment, CUT&RUN reveals expected levels of H3K27me3 enrichment (red track). In cells treated with Fab fragments, the H3K27me3 signal is lost genome-wide (green track). **e**, Genome wide enrichment of H3K27me3 with or without Fab blocking. **f**, Fab blocking does not interfere with subsequent GO-CaRT with LaminB1 antibody. Top blue track: HEK293T cells in which Fab fragments were used to block H3K27me3 antibodies and subsequently incubated with antibodies to LaminB1 protein for LaminB1 GO-CaRT analysis. Bottom blue: HEK293T cells that were directly subjected to LaminB1 GO-CaRT analysis. **g**, Genome-wide scatter plot showing Spearman correlation between LaminB1 GO-CaRT in HEK293T cells after Fab blocking of H3K27me3 vs. those that were directly subjected to LaminB1 GO-CaRT. **h**, Fab blocking eliminates the background signal produced by non-specific targeting of pA/G-MNase by antibodies used in FANS. Representative tracks generated by pA/G-MNase digestion with or without Fab blocking of FANS-isolated EN.

In the developing human cerebral cortex, radial glia (RGs)—the major neural stem cell population—give rise to proliferative intermediate progenitor cells (IPCs) that then produce post-mitotic excitatory neurons (ENs)^24,25^. Using fluorescence-activated nucleus sorting (FANS) with antibodies to PAX6, EOMES and SATB2, we isolated nuclei of RGs (PAX6^+^), IPCs (PAX6^+^EOMES^+^) and ENs (SATB2^+^) from primary cortical brain tissue of gestational week 17 (GW17) and GW20 human fetuses (Extended Data Fig. 1a). RNA sequencing (RNA-seq) analysis performed on FANS-isolated nuclei confirmed the separation and identity of these cell types (Extended Data Fig. 1b).

**Fig. 1:**
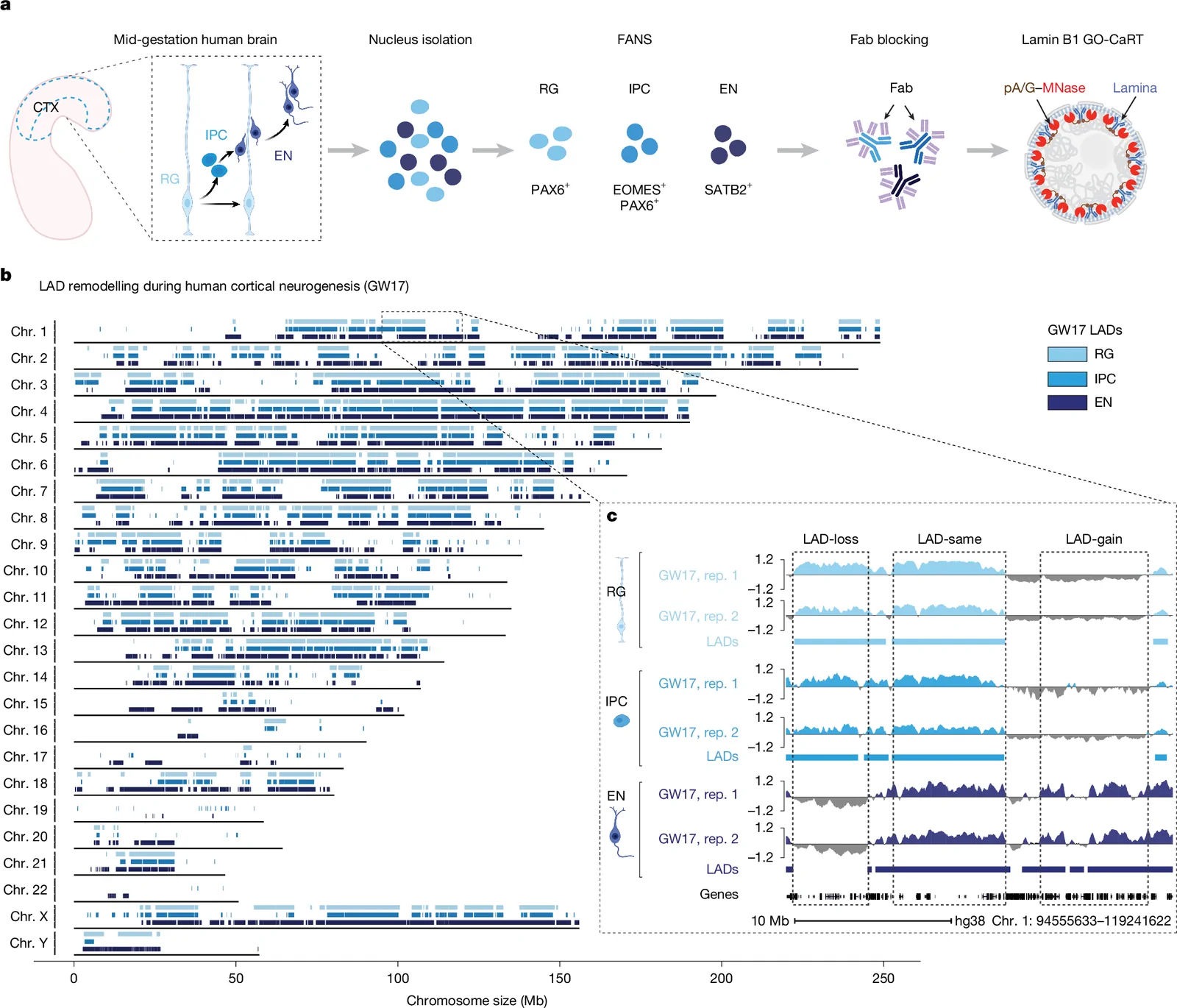
LAD architecture is extensively remodelled during human cortical neurogenesis. **a**, Workflow for FANS-based isolation of RGs, IPCs and ENs, Fab blocking of antibodies used in FANS and downstream lamin B1 GO-CaRT analyses. CTX, cortex. The diagram was created in BioRender; Hamid, S. https://biorender.com/jw25feh (2026). **b**, Distribution of LADs across all human chromosomes at GW17. **c**, Representative lamin B1 GO-CaRT tracks in RGs, IPCs and ENs from two biological replicates (rep.) of GW17 cortex. The *y* axis shows the log_2_-transformed ratio of lamin B1 over IgG. LAD calls generated from the median of the replicates are shown below the tracks by rectangles. The dashed boxes show example genomic regions in which LADs are lost (LAD-loss), gained (LAD-gain) or remain unchanged (LAD-same) during RG-to-EN differentiation.

**Extended Data Fig. 2:**
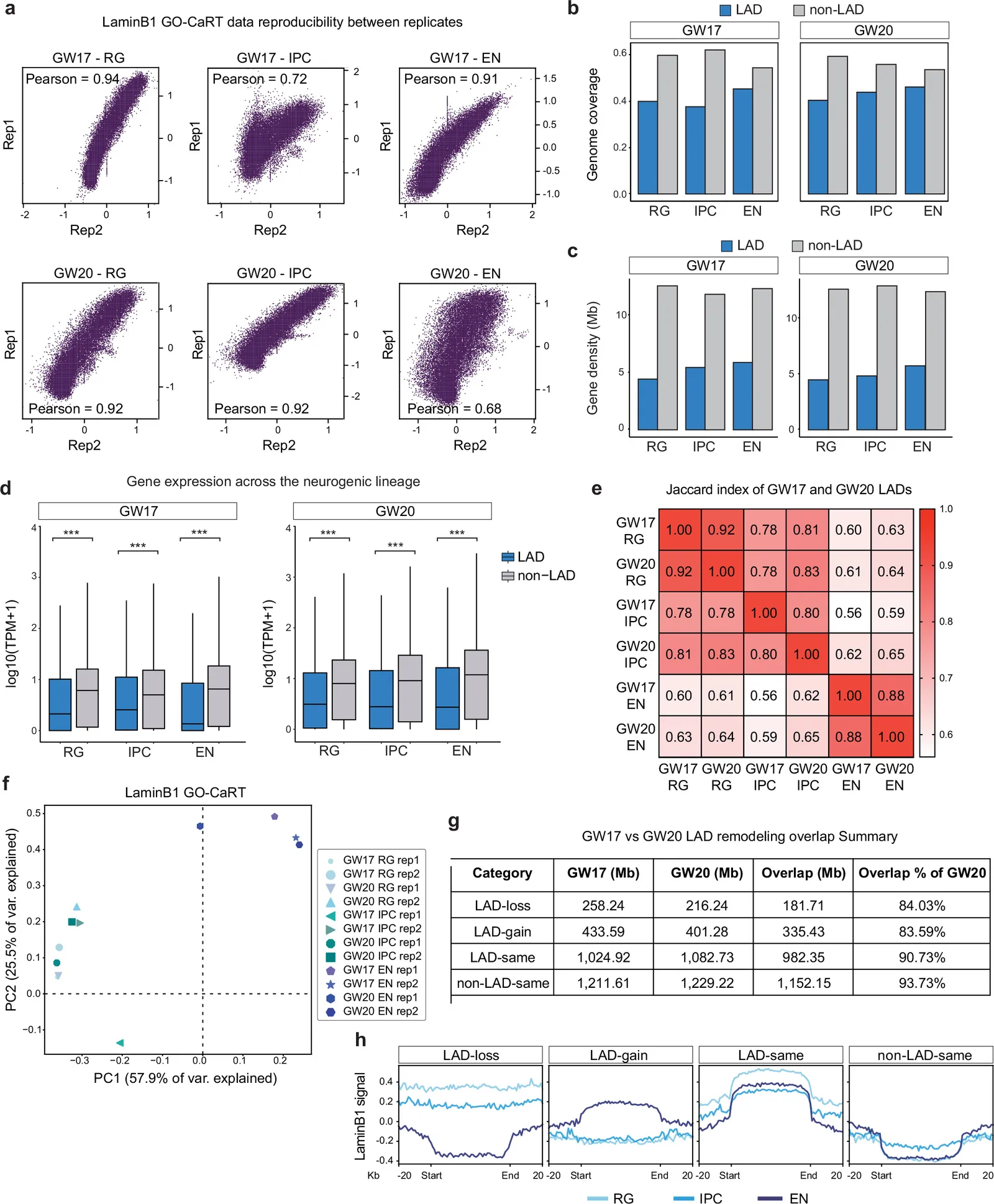
Data reproducibility and genomic features of LADs across the human neurogenic lineage. **a**, Pearson correlation of LaminB1 GO-CaRT profiles of RG, IPC and EN isolated by FANS from GW17 and GW20 cerebral cortex. **b**, Percent LAD coverage in RG, IPC and EN at GW17 and GW20. **c**, Gene density in LADs and non-LADs across the human neurogenic lineage at GW17 and GW20. **d**, Average gene expression in LADs (GW17 n = 3634 genes in RG, 4147 [IPC], and 5435 [EN]; GW20 n = 4606 [RG], 5440 [IPC], and 6871 [EN]) and non-LADs (GW17 n = 15684 [RG], 15173 [IPC], and 13878 [EN]; GW20 n = 21671 [RG], 20853 [IPC], and 19414 [EN]) as determined by RNA-seq. *p*-values determined by two-sided Wilcoxon rank sum test with continuity correction. Boxes denote interquartile range with line at median; whiskers extend 1.5x interquartile range from bounds of box. **e**, Jaccard similarity index of LADs in RG, IPC and EN at GW17 and GW20. **f**, PCA plot of LaminB1 GO-CaRT profiles of RG, IPC and EN at GW17 and GW20. **g**, Summary of LAD remodelling during cortical neurogenesis at GW17 and GW20. **h**, Metagene plot of LaminB1 signal in LAD-loss, LAD-gain, LAD-same and non-LAD-same regions in RG, IPC and EN at GW17.

In genome organization with CUT&RUN technology (GO-CaRT)—a chromatin profiling technology based on cleavage under targets and release using nuclease (CUT&RUN)^26^—antibodies that bind to proteins in specific subnuclear compartments are used to target protein-A/G-fused micrococcal nuclease (pA/G–MNase), resulting in the cutting and release of compartment-associated genomic DNA, which is then identified with next-generation sequencing^7^. We used monovalent fragment antigen-binding (Fab) fragments, which lack the fragment crystallizable (Fc) domain to which pA/G binds, to block antibodies used in FANS before carrying out the GO-CaRT protocol (Extended Data Fig. 1c). Fab fragments were highly efficient at blocking undesired antibody interactions without interfering with downstream GO-CaRT assays (Extended Data Fig. 1d–h). After Fab blocking, we performed lamin B1 GO-CaRT analysis of FANS-isolated nuclei (around 25,000) from two biological replicates each of GW17 and GW20 cortex (Fig. 1a and Supplementary Table 1). Lamin B1 GO-CaRT profiles showed high reproducibility between replicates (Pearson *r* = 0.72–94; Extended Data Fig. 2a). Across the neurogenic lineage, LADs encompassed 40–46% of the genome and exhibited genomic features typical of LADs, including low gene density and expression (Fig. 1b and Extended Data Fig. 2b–d).

**Extended Data Fig. 3:**
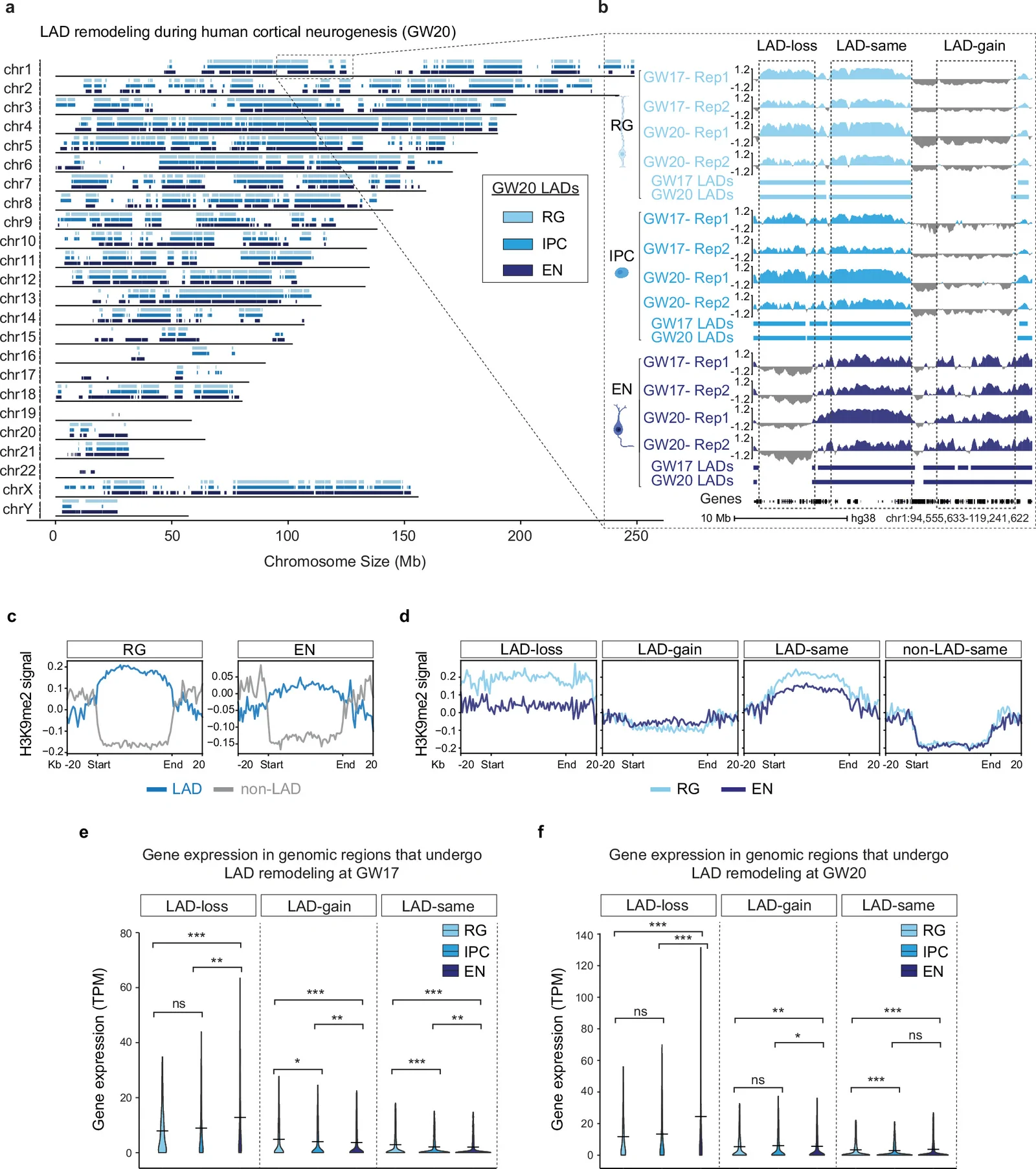
Analysis of chromatin state and gene expression in LAD remodelled regions during neurogenesis at GW17 and GW20. **a**, Chromosome ideogram showing distribution of LADs in RG, IPC and EN across all human chromosomes at GW20. **b**, Representative LaminB1 GO-CaRT tracks in RG, IPC and EN from two biological replicates of GW17 and GW20 cortex. Y-axis depicts log2 ratio of LaminB1 over IgG. LAD calls generated from the median of the replicates are depicted below tracks by rectangles. Dashed boxes show example genomic regions where LADs are lost (LAD-loss), gained (LAD-gain) or remain unchanged (LAD-same) during RG-to-EN differentiation. **c**, Metagene plot of H3K9me2 signal in LADs and non-LADs in RG and EN. **d**, Metagene plot of H3K9me2 signal in LAD-loss, LAD-gain and LAD-same regions in RG, and EN. **e**, Gene expression (in transcripts per million, TPM) in LAD-loss (n = 739 genes), LAD-gain (n = 2561) and LAD-same regions (n = 2912) at GW17. **f**, Gene expression in LAD-loss (n = 555 genes), LAD-gain (n = 2372) and LAD-same regions (n = 3156) at GW20. In **e** and **f**, *p*-values calculated by two-sided continuity-corrected Wilcoxon rank sum test with Benjamini-Hochberg correction. Violin plots extend from min to max values with line at median. Asterisks denote p-values of * p < 0.05, ** p < 0.01, *** p < 0.001.

To identify LAD architectural changes related to neurogenesis, we compared the LAD profiles of RGs, IPCs and ENs at GW17. LADs in proliferative RGs and IPCs were highly similar, whereas postmitotic ENs showed distinct LAD architecture (Extended Data Fig. 2e,f). Accordingly, we focused subsequent analyses on RGs and ENs. During neurogenic differentiation of RGs to ENs, global LAD architecture was extensively remodelled (Fig. 1b,c and Extended Data Fig. 2g). Specifically, 433 Mb (14.0% of the whole genome) moved from the nuclear interior to the lamina (LAD-gain), 258 Mb (8.5% of the genome) was released from the lamina to the nuclear interior (LAD-loss) and 1,024 Mb (33.2% of the genome) remained unchanged (LAD-same). Although RGs and IPCs shared similar LAD architecture, lamin B1 enrichment within LAD-loss regions in IPCs was intermediate to that of RGs and ENs (Extended Data Fig. 2h). Notably, LAD changes observed at GW17 were largely similar to those at GW20 (Extended Data Figs. 2g and 3a,b), indicating that LAD reorganization is a robust feature of human cortical neurogenesis.

The lamina is a transcriptionally repressive compartment and is accordingly enriched for H3K9me2-marked heterochromatin^7,9^. In both RGs and ENs, LADs were enriched for H3K9me2, and H3K9me2 levels were decreased in LAD-loss regions during neurogenesis (Extended Data Fig. 3c,d).

Genes in LAD-loss regions showed significantly increased expression in ENs compared with in RGs (Extended Data Fig. 3e,f). By contrast, genes in LAD-gain regions exhibited mildly decreased expression in IPCs and ENs relative to RGs (Extended Data Fig. 3e,f). Genes in LAD-same regions were expressed at low levels across cell types (Extended Data Fig. 3e,f).

## Neuronal genes move from LADs to SPADs

**Extended Data Fig. 4:**
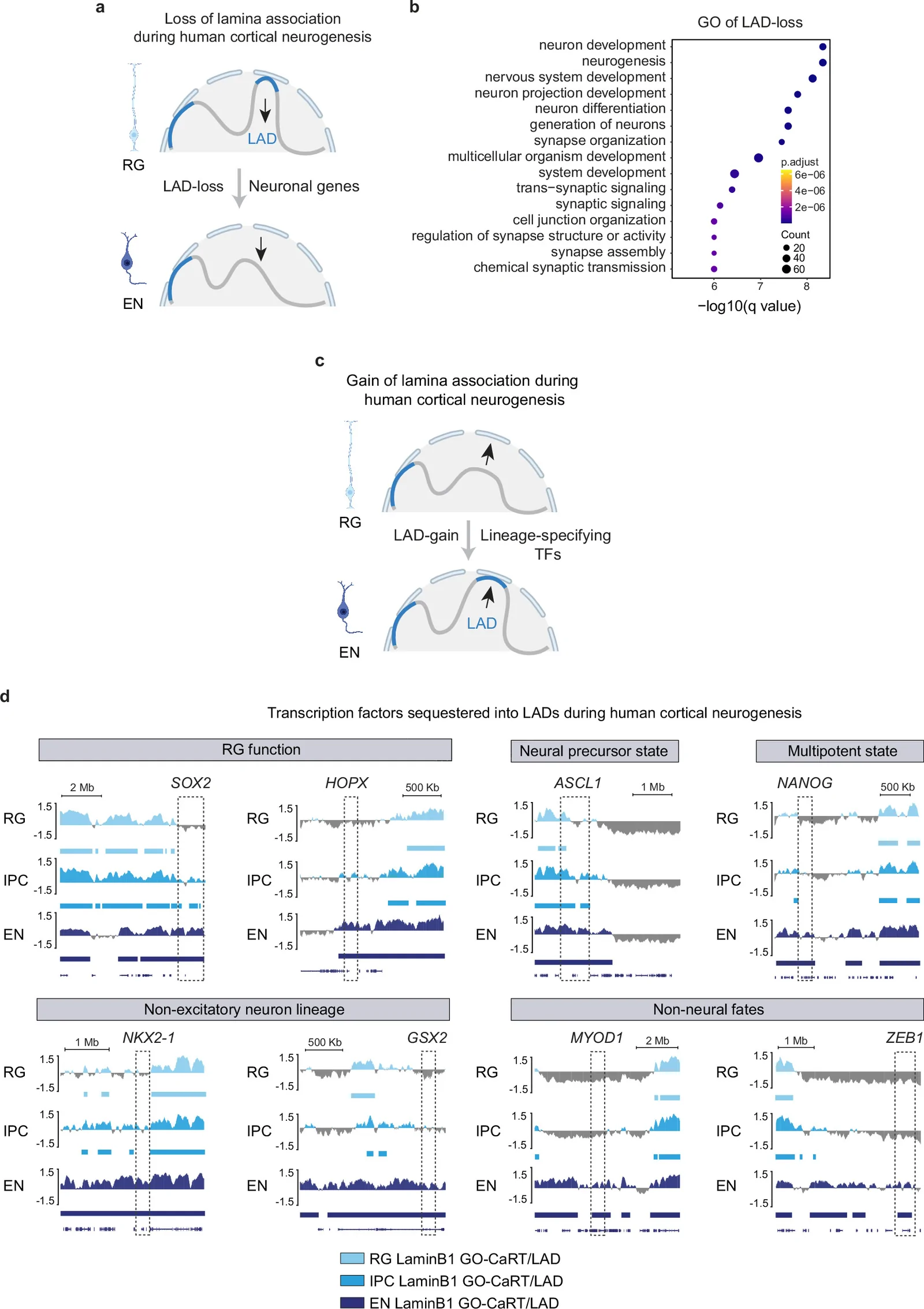
Genes in remodelled LADs relate to neuronal function. **a**, Cartoon illustrating loss of lamina association during RG to EN differentiation. **b**, Gene Ontology (GO) analysis of genes in LAD-loss regions. Top 15 enriched GO terms (biological processes) are shown. GO terms were sorted by significance (− log10(*q* value)); the size of the bubble represents the number of genes from the given ontology present in the dataset. Bubbles are colour-coded by adjusted *p*-value. *p*-values were calculated by one-sided Fisher’s Exact Test and adjusted by Benjamini-Hochberg procedure. Enrichment was considered significant at *p* < 0.05 and *q* < 0.05. **c**, Cartoon illustrating gain of lamina association during RG to EN differentiation. TF, transcription factor. **d**, LaminB1 GO-CaRT tracks in RG, IPC and EN at genes that are sequestered into LADs during cortical neurogenesis. LADs are shown below the track by rectangles. Dashed boxes show indicated gene loci. The diagrams in **a** and **c** were created in BioRender; Hamid, S. https://biorender.com/jw25feh (2026).

During cortical neurogenesis, 739 genes were released from the nuclear lamina. In these LAD-loss regions, Gene Ontology (GO) analysis revealed enrichment of genes involved in neuronal differentiation and development (Extended Data Fig. 4a,b). LAD-loss regions included synaptic genes such as *ANKS1B*,* NRG3*,* SYT1*,* SLITRK1* and *LRRC7* and transcription factors with well-known roles in cortical neurogenesis such as *MEF2C* and *SATB2*. Genes (*n* = 2,561) that gained lamina association during neurogenesis were not significantly enriched for any specific ontologies, but we noted that key transcription factors involved in RG function (*SOX2*,* HOPX*), multipotent state (*NANOG*), neural precursor state (*ASCL1*), non-excitatory neuron lineages (*NKX2-1*, *NKX2-2* and *GSX2*) and non-neural fates (*MYOD1*, *ZEB1*) were located in LAD-gain regions (Extended Data Fig. 4c,d).

**Fig. 2:**
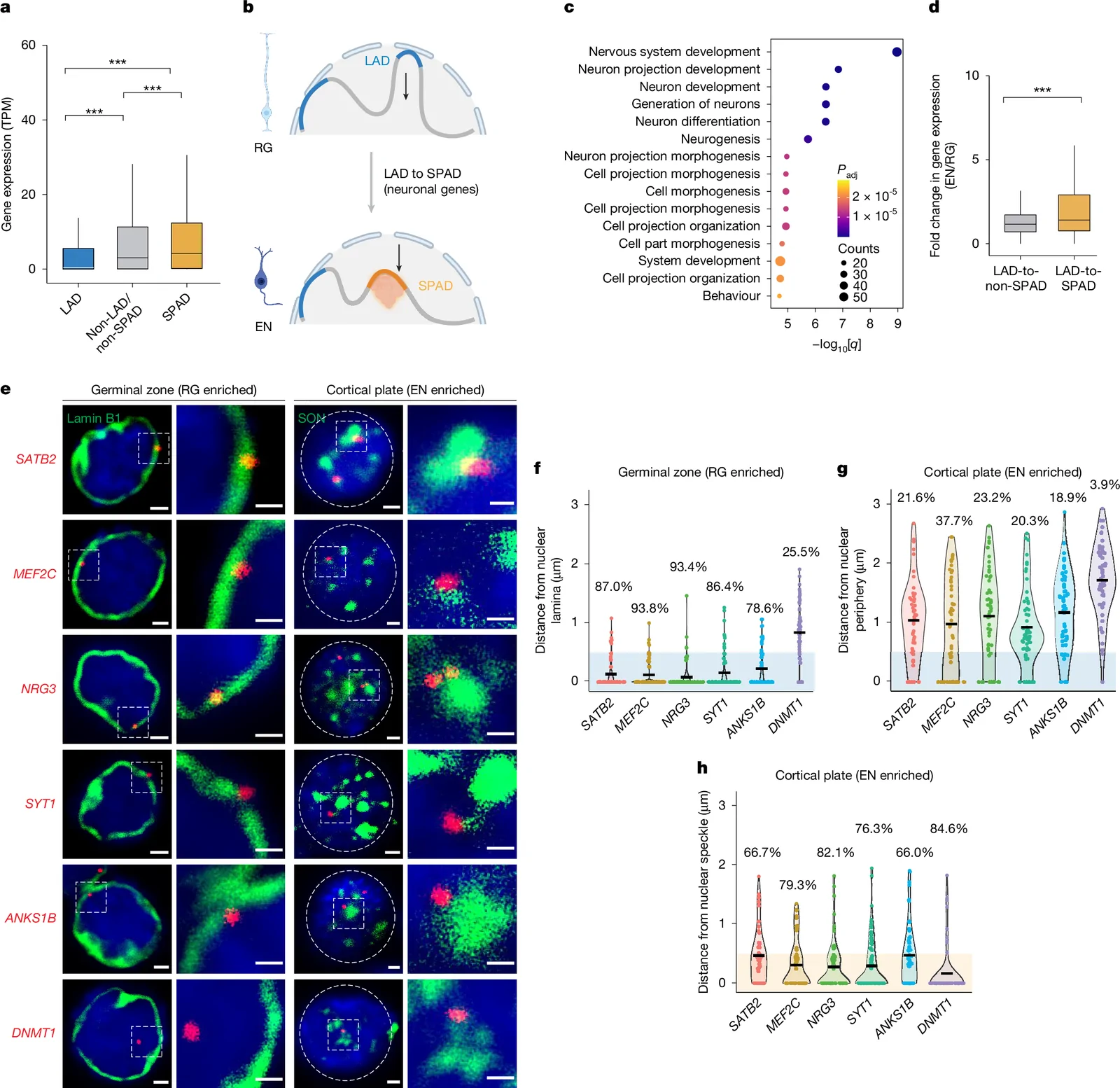
Neuronal genes move from the lamina to speckles during cortical neurogenesis. **a**, Gene expression in LADs (*n* = 3,333 (RGs), 3,746 (IPCs) and 4,842 (ENs) genes), non-LADs/non-SPADs (*n* = 5,555 (RGs), 5,847 (IPCs) and 11,383 (ENs) genes) and SPADs (*n* = 8,862 (RGs), 8,292 in (IPCs) and 1,431 (ENs)) across the neurogenic lineage. TPM, transcripts per million. **b**, Diagram of the LAD-to-SPAD switch during RG-to-EN differentiation. The diagram was created in BioRender; Hamid, S. https://biorender.com/jw25feh (2026). **c**, GO analysis of genes in LAD-to-SPAD regions. The top 15 enriched GO terms (biological processes) are shown. GO terms were sorted by significance (−log_10_[*q*]); the size of the bubble represents the number of genes from the given ontology present in the dataset. Bubbles are colour-coded by adjusted *P* value. *P* values were calculated using one-sided Fisher’s exact tests and adjusted using the Benjamini–Hochberg procedure. Enrichment was considered significant at *P* < 0.05 and *q* < 0.05. **d**, Gene expression fold change in regions that switch from LAD to non-SPAD (*n* = 434 genes) or LAD to SPAD (*n* = 305) during RG-to-EN differentiation. **e**, Representative DNA-FISH micrographs of the indicated gene loci (red) with lamin B1 and SON immunostaining (green) in cells of the germinal zone and cortical plate at GW17. Scale bars, 2 μm (lower magnification) and 0.5 μm (higher magnification). DAPI staining is visible in blue. **f**–**h**, Quantifications of DNA-FISH loci shown in **e**. The percentages indicate loci within 0.5 μm of the nuclear lamina (germinal zone; **f**), nuclear periphery (cortical plate; **g**) or nuclear speckles (cortical plate; **h**). For each locus, 40–50 nuclei were quantified. In **a** and **d**, *P* values were calculated using two-sided continuity-corrected Wilcoxon rank-sum tests with Benjamini–Hochberg correction; *P < 0.05, **P < 0.01, ***P < 0.001. The box plots show the median (centre line), the interquartile range (box limits) and the whiskers extend to 1.5× the interquartile range from bounds of box.

**Extended Data Fig. 5:**
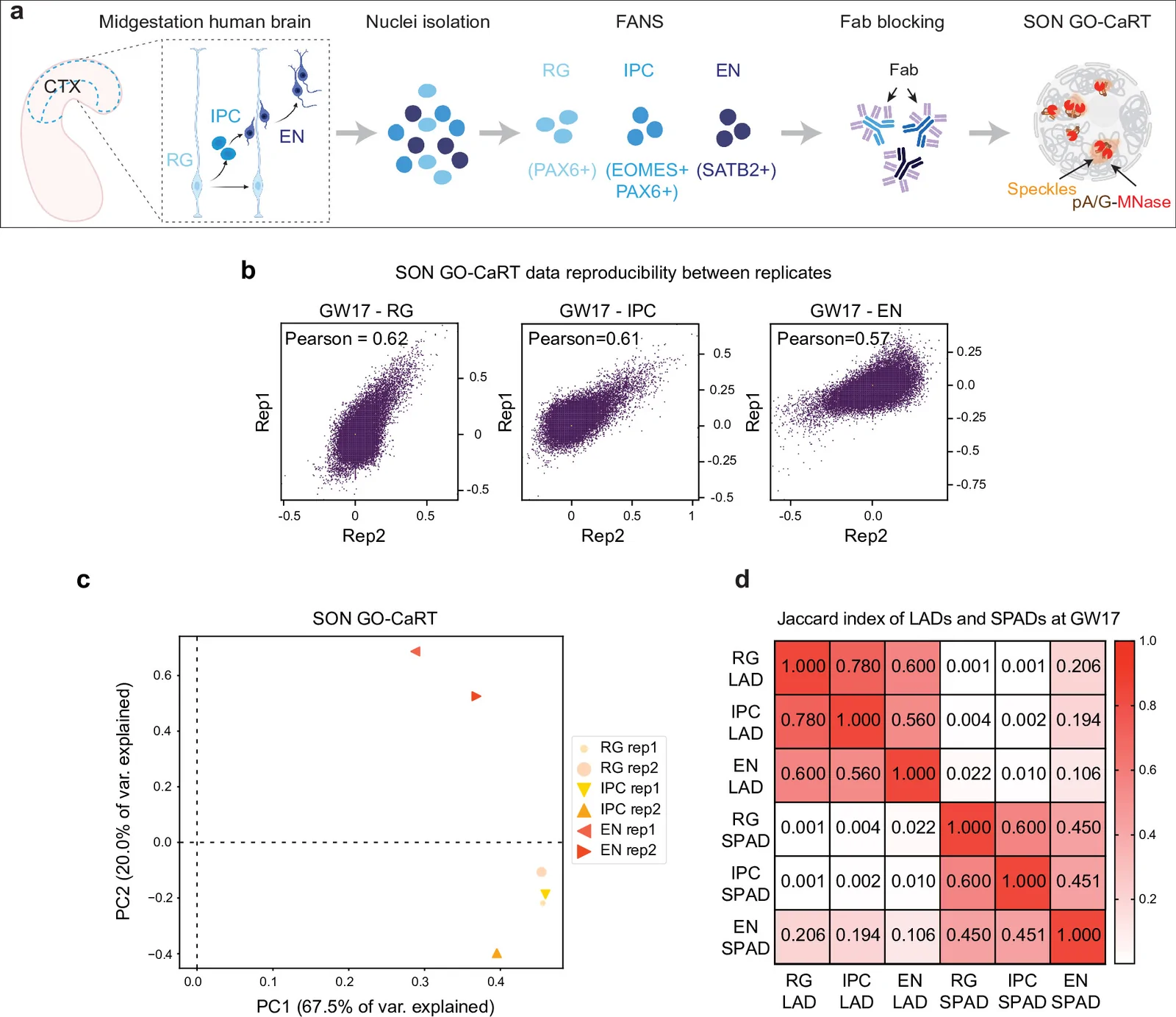
Profiling of SPAD architecture across the human neurogenic lineage by SON GO-CaRT. **a**, Schematic of workflow for FANS-based isolation of RG, IPC and EN, Fab blocking of antibodies used in FANS and downstream SON GO-CaRT analyses. The diagram was created in BioRender; Hamid, S. https://biorender.com/jw25feh (2026). **b**, Pearson correlation of SON GO-CaRT profiles of RG, IPC and EN isolated by FANS from GW17 cerebral cortex. **c**, PCA plot of SON GO-CaRT profiles of RG, IPC and EN at GW17 and GW20. **d**, Jaccard similarity index of LADs and SPADs in RG, IPC and EN at GW17.

**Extended Data Fig. 6:**
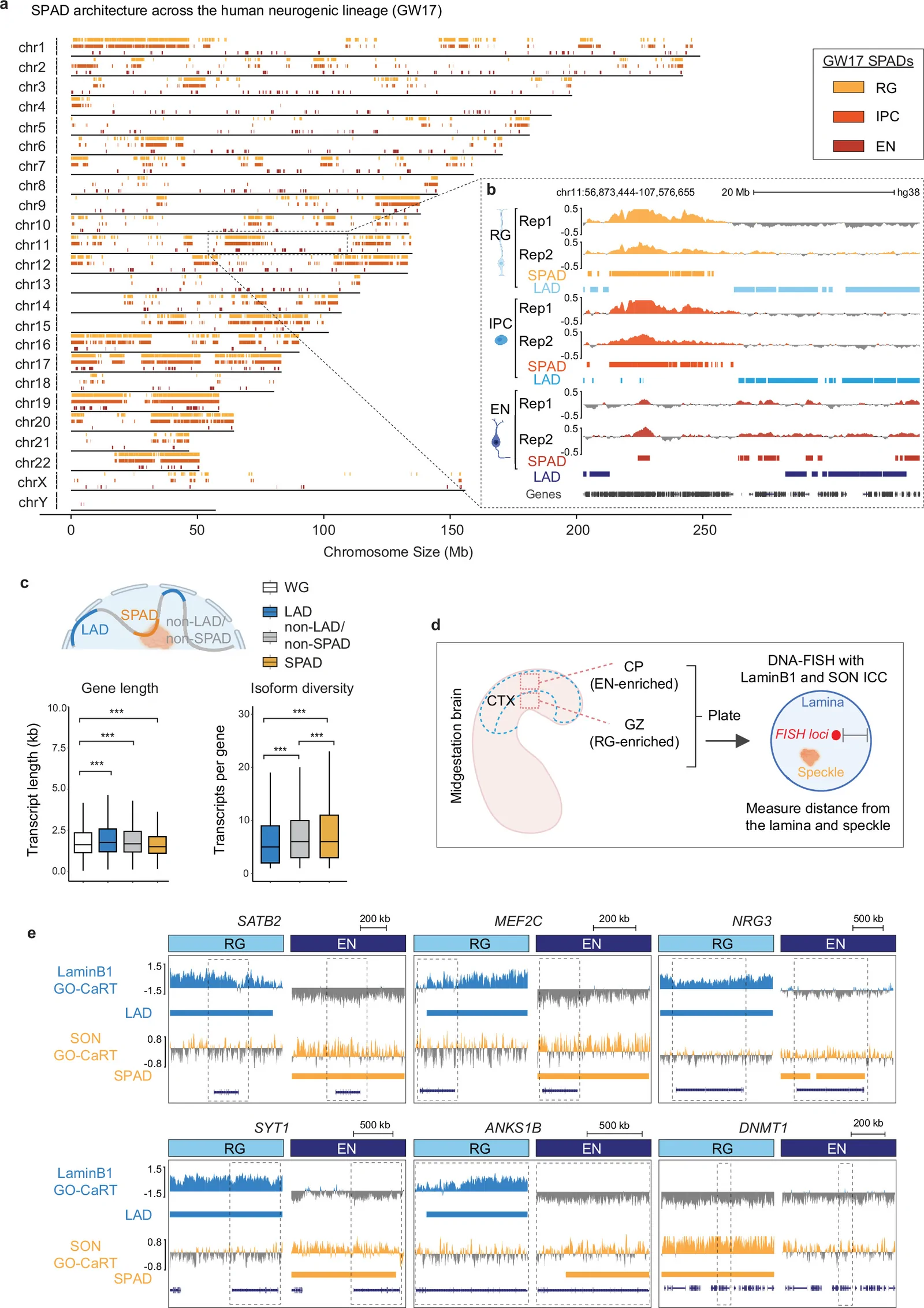
SPAD architecture across the human neurogenic lineage and neurogenic LAD-to-SPAD switching. **a**, Chromosome ideogram of SPADs across the human neurogenic lineage at GW17. SPADs are indicated by rectangles of light orange for RG, medium light orange for IPC and dark orange for EN. **b**, Representative SON GO-CaRT tracks in RG, IPC and EN showing LADs and SPADs are largely non-overlapping regions of the genome across the neurogenic lineage. SPAD (shades of orange) and LAD calls (shades of blue) are depicted below the tracks for comparison. **c**, Box plot showing average transcript length and isoform diversity in LADs (n = 3438 genes in RG, 3939 [IPC], 5188 [EN]), non-LADs/non-SPADs (n = 5791 [RG], 6165 [IPC], 11862 [EN]) and SPADs (n = 9160 [RG], 8640 [IPC], 1509 [EN]) across the neurogenic lineage. Non-LADs/non-SPADs are genomic regions that neither associate with lamina nor speckles (cartoon on top). *p*-values calculated by two-sided continuity-corrected Wilcoxon rank sum test with Benjamini-Hochberg correction. Boxes denote interquartile range with line at median; whiskers extend 1.5x interquartile range from bounds of box. Asterisks denote *p*-values of *** *p* < 0.001. **d**, Cartoon showing the experimental design for DNA-FISH. **e**, LaminB1 and SON GO-CaRT tracks in RG and EN over gene loci analysed by DNA-FISH. The diagrams in **c** and **d** were created in BioRender; Hamid, S. https://biorender.com/jw25feh (2026).

After release from the nuclear lamina, genomic domains may associate with other subnuclear compartments such as nuclear speckles. To examine SPAD architecture, we performed GO-CaRT using an antibody against SON^7^—a nuclear speckle scaffold protein—in RGs, IPCs and ENs isolated from two biological replicates of GW17 cortical tissue (Extended Data Fig. 5a). The Pearson correlation between SON GO-CaRT replicates ranged from 0.57 to 0.62, and RG and IPC profiles were more similar to each other than to ENs (Extended Data Fig. 5b,c). Across the neurogenic lineage, chromosomes were partitioned into largely non-overlapping LADs and SPADs interspersed with non-LAD/non-SPAD regions (genomic regions that associate with neither lamina nor speckles) (Extended Data Fig. 5d and Extended Data Fig. 6a,b). Consistent with previous reports^2,27^, genes within SPADs showed significantly higher expression levels, were shorter and exhibited greater isoform diversity than those in LADs or non-LAD/non-SPAD regions (Fig. 2a and Extended Data Fig. 6c).

Among the 739 genes that were released from the nuclear lamina during neurogenesis, 41% (305 genes) relocated to nuclear speckles. GO analysis revealed this set of LAD-to-SPAD-switching genes was enriched in functions related to neuron development, neurogenesis and synaptic function (Fig. 2b,c). By contrast, the set of genes (434) that relocated to non-LAD/non-SPAD regions was not significantly enriched in any specific ontology. Furthermore, compared with non-LAD/non-SPAD regions, genes that relocated to SPADs were expressed at higher levels (Fig. 2d).

Using DNA fluorescence in situ hybridization (FISH) combined with immunocytochemistry, we examined the nuclear positioning of five neuronal gene loci—*SATB2*,* MEF2C*,* ANKS1B*,* NRG3* and *SYT1*—that relocated from LADs in RGs to SPADs in ENs (Extended Data Fig. 6d,e). All five loci were preferentially localized to the lamin B1^+^ nuclear periphery in cells of the germinal zone (RG-enriched) (Fig. 2e,f). In the cortical plate (EN-enriched), these loci were distant to the nuclear periphery and instead localized to SON^+^ nuclear speckles (Fig. 2e,g,h). The non-LAD *DNMT1* locus was localized away from the nuclear periphery and close to speckles in both germinal zone and cortical plate cells (Fig. 2e–h). These results validate our GO-CaRT data and the movement of neuronal genes from the lamina to speckles during neurogenesis.

## LAD bivalent genes have low expression

**Extended Data Fig. 7:**
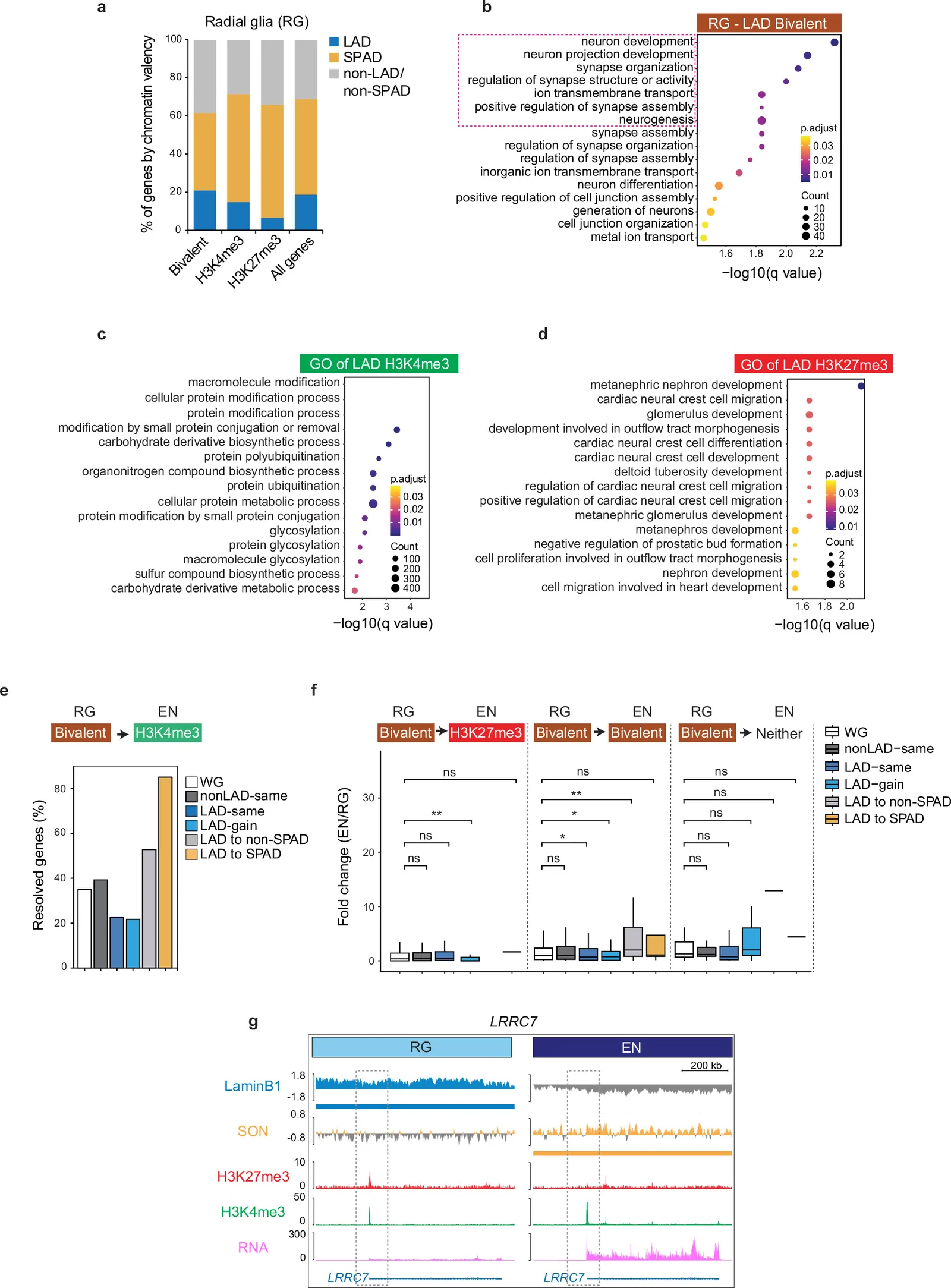
LAD-to-SPAD relocation during cortical neurogenesis. **a**, Percentage of genes stratified by chromatin state in indicated genomic compartments in RG. **b-d**, GO analyses of bivalent (**b**), H3K4me3-monovalent (**c**) and H3K27me3-monovalent (**d**) genes in RG LADs. GO terms were sorted by significance (− log10(*q* value)); the size of the bubble represents the number of genes from the given ontology present in the dataset. Bubbles are colour-coded by adjusted *p*-value. *p*-values were calculated by one-sided Fisher’s Exact Test and adjusted by Benjamini-Hochberg procedure. GO term enrichment was considered significant at *p* < 0.05 and *q* < 0.05. **e**, Percentage of RG bivalent genes that resolve to the H3K4me3-monovalent state in EN stratified by LAD-to-SPAD dynamics. **f**, Fold change of expression of bivalent genes that resolve to the H3K27me3-monovalent state (n = 211, 141, 30, 39, 0, and 1 genes), remain bivalent (n = 1569, 979, 290, 262, 34, and 4), or lose both marks (n = 38, 23, 10, 3, 1, and 1) during RG to EN differentiation stratified by LAD to SPAD dynamics. *p*-values calculated by two-sided continuity-corrected Wilcoxon rank sum test with Benjamini-Hochberg correction; asterisks denote *p*-values of * *p* < 0.05, ** *p* < 0.01; boxes show interquartile range with line at median; whiskers extend 1.5x interquartile range from bounds of box. **g**, LaminB1 GO-CaRT, SON GO-CaRT, H3K27me3, H3K4me3 (CUT&RUN) and RNA-seq tracks at *LRRC7*, an RG LAD bivalent gene that relocates to SPADs in EN and resolves to the H3K4me3-monovalent state with strong transcriptional upregulation.

Many genes in LAD-loss regions are key regulators of neurogenesis. In progenitor cells, the promoters of developmental genes are frequently marked by bivalent chromatin. We accordingly performed CUT&RUN for H3K4me3 and H3K27me3 in RGs, IPCs and ENs from GW17 cortex and identified bivalent genes containing peaks for both marks. In RGs, we identified 2,675 bivalent genes, of which 560 (around 21%) were located within LADs, a moderate enrichment over H3K4me3-monovalent and H3K27me3-monovalent genes (Extended Data Fig. 7a). These lamina-associated bivalent genes were enriched for GO terms related to neurodevelopment, including neuronal differentiation, projection and synapse organization (Extended Data Fig. 7b). By contrast, LAD genes monovalent for H3K4me3 or H3K27me3 were not enriched for such terms (Extended Data Fig. 7c,d).

**Fig. 3:**
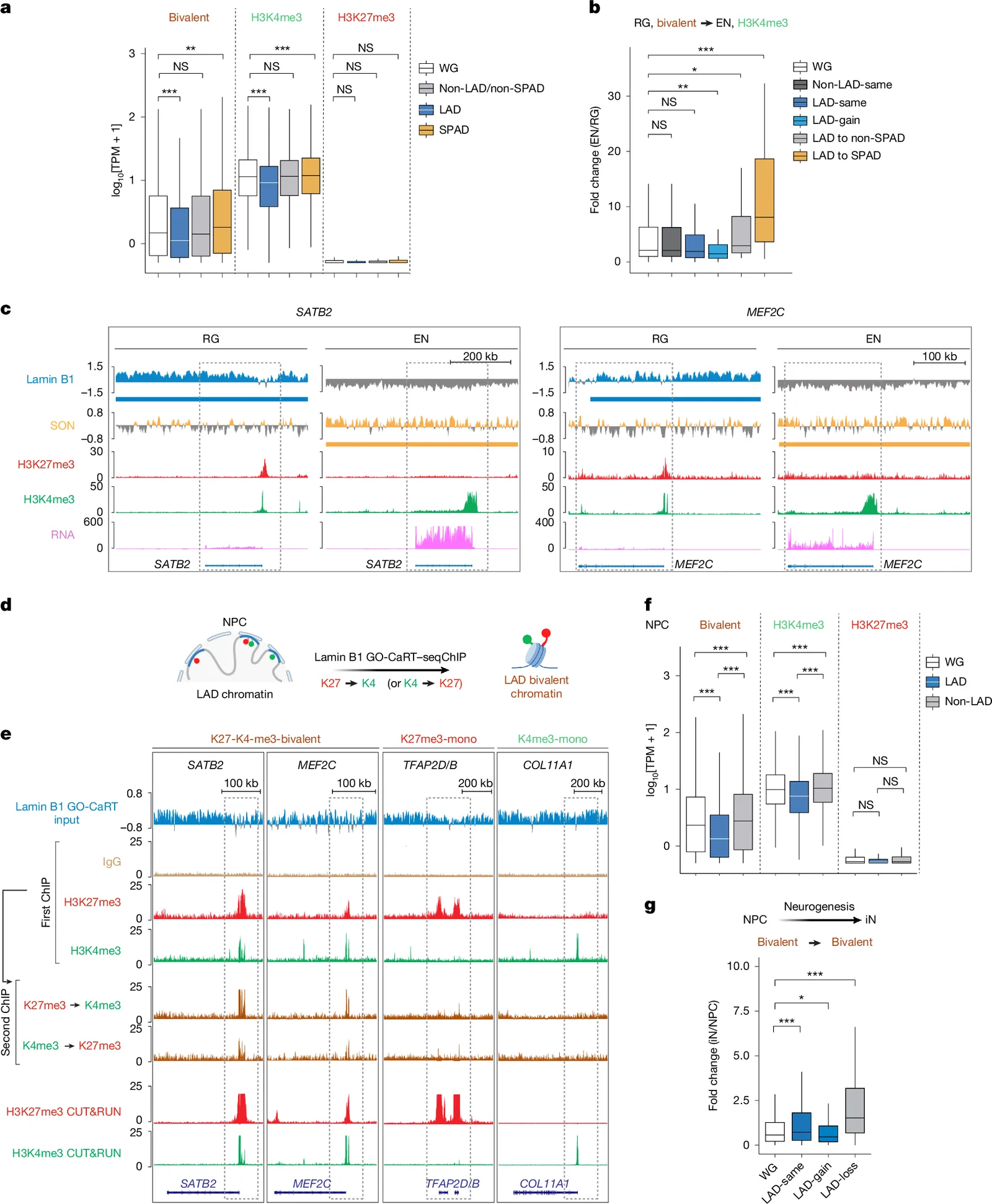
LAD and SPAD dynamics regulate the bivalent chromatin state. **a**, Expression of RG bivalent (*n* = 2,652 genes in the whole genome: 550 (LAD), 1,024 (non-LAD/non-SPAD) and 1,076 (SPAD)), H3K4me3-monovalent (*n* = 10,192 genes in the whole genome: 1,570 (LAD), 2,910 (non-LAD/non-SPAD) and 5,732 (SPAD)) and H3K27me3-monovalent (*n* = 1,468 genes in the whole genome: 97 (LAD), 502 (non-LAD/non-SPAD) and 859 (SPAD)) genes. **b**, Fold change in expression of bivalent genes that resolve to the H3K4me3-monovalent state (from left to right, *n* = 914, 658, 96, 83, 38 and 39 genes) during RG-to-EN differentiation stratified by LAD to SPAD dynamics. **c**, GO-CaRT, CUT&RUN and RNA-seq tracks at example LAD-to-SPAD switching genes (*SATB2* and* MEF2C*) that resolve from bivalency to H3K4me3 monovalency. **d**, Schematic of lamin B1 GO-CaRT–seqChIP in NPCs: lamin B1 GO-CaRT-released chromatin was used as an input for sequential chromatin immunoprecipitation (ChIP), first with anti-H3K27me3 followed by anti-H3K4me3 (or vice versa). The diagram was created in BioRender; Hamid, S. https://biorender.com/jw25feh (2026). **e**, Lamin B1 GO-CaRT–seqChIP profiles at the bivalent LAD genes *SATB2* and *MEF2C*. H3K27me3-monovalent (*TFAP2D/B*) and H3K4me3-monovalent (*COL11A1*) genes are not enriched by seqChIP. IgG was used as a negative control for ChIP. Parallel H3K27me3 and H3K4me3 CUT&RUN tracks are also shown for comparison. **f**, Expression of bivalent (*n* = 4,395 genes in the whole genome: 3,521 (non-LAD) and 874 (LAD)), H3K4me3-monovalent (*n* = 8,441 genes in the whole genome: 6,866 (non-LAD) and 1,587 (LAD)) and H3K27me3-monovalent (*n* = 764 genes in the whole genome: 708 (non-LAD) and 56 (LAD)) genes in NPCs. **g**, Fold change in the expression of NPC bivalent genes that maintain bivalency in induced neurons (iN; *n* = 2,423 genes in the whole genome: 545 (LAD-same), 307 (LAD-gain) and 53 (LAD-loss)). For **a**, **b**,** f** and **g**, *P* values were calculated using two-sided continuity-corrected Wilcoxon rank-sum tests with Benjamini–Hochberg correction. The box plots show the median (centre line), the interquartile range (box limits) and the whiskers extend to 1.5× the interquartile range from bounds of box. NS, not significant.

As expected, H3K4me3-monovalent genes had higher expression than those monovalent for H3K27me3, with bivalent genes expressed at intermediate levels (Fig. 3a). For H3K27me3-monovalent genes, location with respect to the lamina did not correspond to major differences in expression (Fig. 3a). For H3K4me3-monovalent genes, LAD localization corresponded to a slight decrease in expression compared to the whole genome or non-LAD regions (Fig. 3a). Notably, for bivalent genes, location within LADs corresponded to significantly lower levels of expression than in those across the genome or in non-LADs (Fig. 3a). Thus, bivalent genes in LADs are expressed at much lower levels, suggesting that this spatial aspect of genome organization reinforces their lowly expressed, poised state.

## LAD-to-SPAD switching resolves bivalency

Resolution of bivalent genes to the H3K4me3-monovalent state during cellular differentiation is thought to increase gene expression, but this increase is often modest and highly variable^20,23^. Of the 2,675 bivalent genes in RGs, 937 (35%) resolved to H3K4me3-monovalent in ENs, corresponding to an approximately twofold average increase in gene expression (Fig. 3b and Extended Data Fig. 7e,f). In regions located outside of LADs in both RGs and ENs, 39% of bivalent genes resolved to H3K4me3 monovalency with expression increases similar to the whole-genome average (Fig. 3b and Extended Data Fig. 7e,f). By contrast, fewer bivalent genes resolved to the H3K4me3-monovalent state in LAD-same (22%) and LAD-gain (21%) regions, with only modest increases in gene expression (Fig. 3b and Extended Data Fig. 7e,f).

Notably, resolution of bivalency and transcriptional upregulation were both significantly higher in LAD-loss regions. In the subset of LAD-loss regions that relocated to non-LAD/non-SPADs, 53% of RG bivalent genes became H3K4me3-monovalent, exhibiting an approximately threefold increase in gene expression in ENs (Fig. 3b and Extended Data Fig. 7e,f). In the subset of LAD-loss regions that relocated to SPADs in ENs, 85% of RG bivalent genes resolved to the H3K4me3-monovalent state with an approximately eightfold increase in expression—significantly higher than all other spatial categories (Fig. 3b and Extended Data Fig. 7e,f). Examples of RG bivalent genes that relocated to EN SPADs while resolving to H3K4me3 monovalency included neurodevelopmental transcription factors *SATB2* and *MEF2C* and synaptic gene *LRRC7* (Fig. 3c and Extended Data Fig. 7g). Thus, movement of genomic regions from the lamina to nuclear speckles during neurogenesis strongly corresponds to the resolution of resident bivalent genes to the H3K4me3-monovalent state and increased transcriptional output.

## GO-CaRT–seqChIP validates LAD bivalency

**Extended Data Fig. 8:**
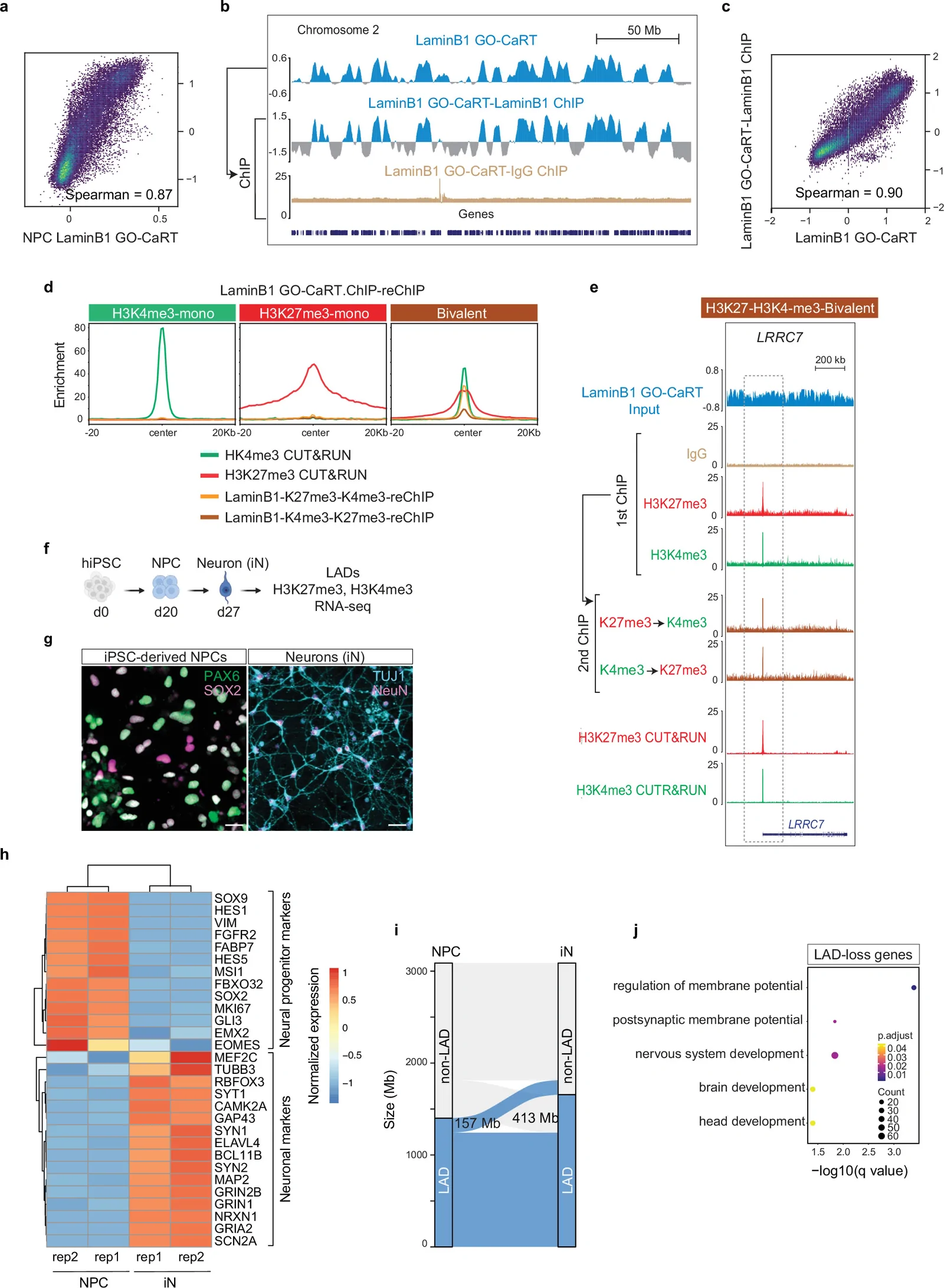
LaminB1 GO-CaRT-sequential ChIP and validation of in vitro neuronal differentiation paradigm. **a**, Genome-wide scatter plot of Spearman correlation between LaminB1 GO-CaRT in iPSC-derived NPCs and LaminB1 GO-CaRT in RG isolated from GW17 cortex. **b**, Representative genome tracks of LaminB1 GO-CaRT and subsequent LaminB1 ChIP and IgG ChIP performed on LaminB1 GO-CaRT supernatant (input). **c**, Genome-wide scatter plot of Spearman correlation between LaminB1 GO-CaRT input and LaminB1 GO-CaRT-LaminB1 ChIP. **d**, Metagene plots of LaminB1 GO-CaRT.ChIP-reChIP signal at H3K4me3-monovalent, H3K27me3-monovalent, and bivalent genes as defined by independent CUT&RUN profiling for H3K4me3 and H3K27me3. **e**, LaminB1 GO-CaRT.ChIP-reChIP profiles at neuronal LAD gene *LRRC7*. The following tracks are shown: LaminB1 GO-CaRT input, first ChIP with H3K27me3 and H3K4me3, H3K4me3 sequential ChIP on H3K27me3 first ChIP and H3K27me3 sequential ChIP on H3K4me3 first ChIP. IgG was used as a negative control for ChIP. Parallel H3K27me3 and H3K4me3 CUT&RUN tracks are also shown for comparison. **f**, Schematic of synchronous neuronal differentiation of iPSC-derived NPCs and profiling of LADs, histone marks and RNA-seq in NPC and induced neurons (iN). The diagram was created in BioRender; Hamid, S. https://biorender.com/jw25feh (2026). **g**, Representative images of immunofluorescence staining for NPC and neuronal marker genes in iPSC-derived NPC (left, scale bar 20 µm) and induced neurons (iN, right, scale bar 40 µm). Images are representative of two independent differentiations. **h**, Heatmap showing the expression (row normalized) of key NPC and neuronal marker genes in NPCs and iN. **i**, Alluvial plot depicting LAD changes (LAD-loss and LAD-gain) during the differentiation of NPC to iN. **j**, GO analysis of genes that reside in genomic regions that undergo LAD-loss during in vitro neurogenesis from (during NPC to iN differentiation). GO terms were sorted by significance (−log10(*q* value)); the size of the bubble represents the number of genes from the given ontology present in the dataset. Bubbles are colour-coded by adjusted *p*-value. *p*-values were calculated by one-sided Fisher’s Exact Test and adjusted by Benjamini-Hochberg procedure. GO term enrichment was considered significant at *p* < 0.05 and *q* < 0.05.

Parallel CUT&RUN assays for H3K4me3 and H3K27me3 cannot unequivocally establish the presence of both marks on the same chromatin fragment. To confirm the bivalent chromatin state of LAD genes, we first conducted lamin B1 GO-CaRT in neural progenitor cells (NPCs) derived from human induced pluripotent stem (hiPS) cells. The lamin-B1 GO-CaRT profile of iPS-cell-derived NPCs was highly similar to that of RGs (Extended Data Fig. 8a; Spearman* ρ* = 0.87).

We next conducted sequential chromatin immunoprecipitation (seqChIP) analysis of lamin B1 GO-CaRT-released chromatin, performing H3K27me3 ChIP followed by H3K4me3 ChIP, and vice versa (Fig. 3d and Extended Data Fig. 8b,c). Of the LAD genes called bivalent by parallel H3K4me3 and H3K27me3 CUT&RUN analysis, 88% (338 out of 384) were confirmed by lamin B1 GO-CaRT–seqChIP (Extended Data Fig. 8d). This subset included neural transcription factors *SATB2* and *MEF2C* and synaptic gene *LRRC7* (Fig. 3e and Extended Data Fig. 8e). By contrast, *COL11A1* and *TFAP2D/B*—genes monovalent for H3K4me3 and H3K27me3, respectively—were not enriched by lamin B1 GO-CaRT–seqChIP (Fig. 3e), demonstrating the specificity of seqChIP on lamin-B1-enriched chromatin. These results confirm that LADs contain bona fide bivalent chromatin at genes important for neuronal differentiation.

## LADs regulate bivalency in vitro

To further study how LAD dynamics influence the bivalent chromatin state, we generated profiles of LADs, H3K4me3 and H3K27me3 during synchronous differentiation of hiPS-cell-derived NPCs into cortical neurons^28^ (Extended Data Fig. 8f–h). After differentiation for 1 week, 157 Mb of the genome dissociated from the lamina (LAD-loss, 5.1% of the genome), 413 Mb gained lamina association (LAD-gain, 13.4% of the genome) and 781 Mb remained unchanged (LAD-same, 40.2% of the genome) (Extended Data Fig. 8i). LAD-loss regions were enriched for neuronal GO terms (Extended Data Fig. 8j).

**Extended Data Fig. 9:**
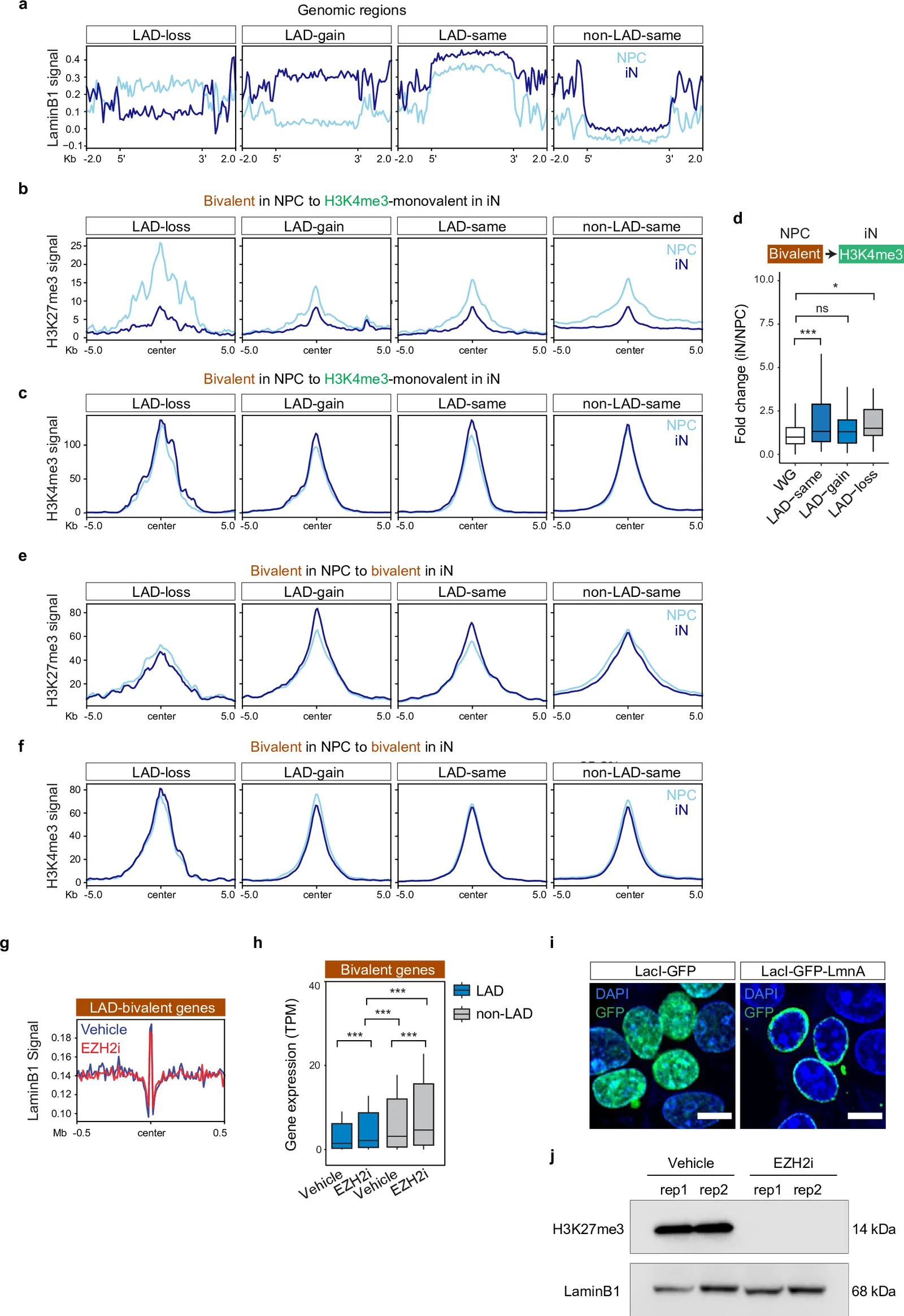
LAD dynamics and regulation of bivalent chromatin in vitro during neurogenesis and EZH2 inhibition. **a**, Metagene plots of normalized LaminB1 signal in LAD-loss, LAD-gain, LAD-same, and non-LAD-same regions in NPC and iN. **b, c**, Metagene plots of H3K27me3 (**b**) and H3K4me3 (**c**) signal at bivalent genes that resolve to the H3K4me3-monovalent state during NPC-to-iN differentiation. **d**, Fold change in expression of NPC bivalent genes in LAD-loss regions that resolve to H3K4me3-monovalent state in iN (n = 981 genes in WG, 99 in LAD-same, 75 in LAD-gain, 17 in LAD-loss). **e, f**, Metagene plots of H3K27me3 (**e**) and H3K4me3 (**f**) signal at bivalent genes that retain bivalency during NPC-to-iN differentiation. **g**, Normalized LaminB1 levels at LAD bivalent genes in NPCs treated with DMSO and 1 μM tazemetostat (EZH2i) for 7 DIV. **h**, Box plot showing average gene expression of bivalent genes in LADs and non-LADs in NPCs treated with DMSO or 1 μM tazemetostat (EZH2i) for 7 DIV. *n* = 567 genes (LAD), 2158 (non-LAD). **i**, Representative images of immunofluorescence staining for GFP in HEK293T cells transfected with either LacI-GFP or LacI-LmnA. Images are representative of two biologically independent transfections. Scale bars, 10 µm. **j**, Western blotting for H3K27me3 in HEK293T cells treated with DMSO or 5 μM tazemetostat (EZH2i) for 7 DIV. LaminB1 is shown as a loading control. Blot shows two independent biological replicates of each condition run on same gel. In **f** and **h**, *p*-values calculated by two-sided continuity-corrected Wilcoxon rank sum test with Benjamini-Hochberg correction. Boxes denote interquartile range with line at median; whiskers extend 1.5x interquartile range from bounds of box. Asterisks denote *p*-values of * *p* < 0.05, *** *p* < 0.001.

In agreement with our findings in primary cells, bivalent genes in NPCs were expressed at lower levels in LADs than in non-LADs or the whole genome (Fig. 3f). Genes monovalent for H3K4me3 and H3K27me3 exhibited high and low expression, respectively, with lower levels in LADs than in non-LADs (Fig. 3f). During differentiation, bivalent genes in LAD-loss regions that resolved to the H3K4me3-monovalent state increased expression (Extended Data Fig. 9a–d). Importantly, genes that remained bivalent in LAD-loss regions also increased their expression during differentiation (Fig. 3g and Extended Data Fig. 9a,e,f). Together, these data support the notion that lamina association contributes prominently to the low-expression, poised state of bivalency.

## LAD repression is H3K27me3 independent

**Fig. 4:**
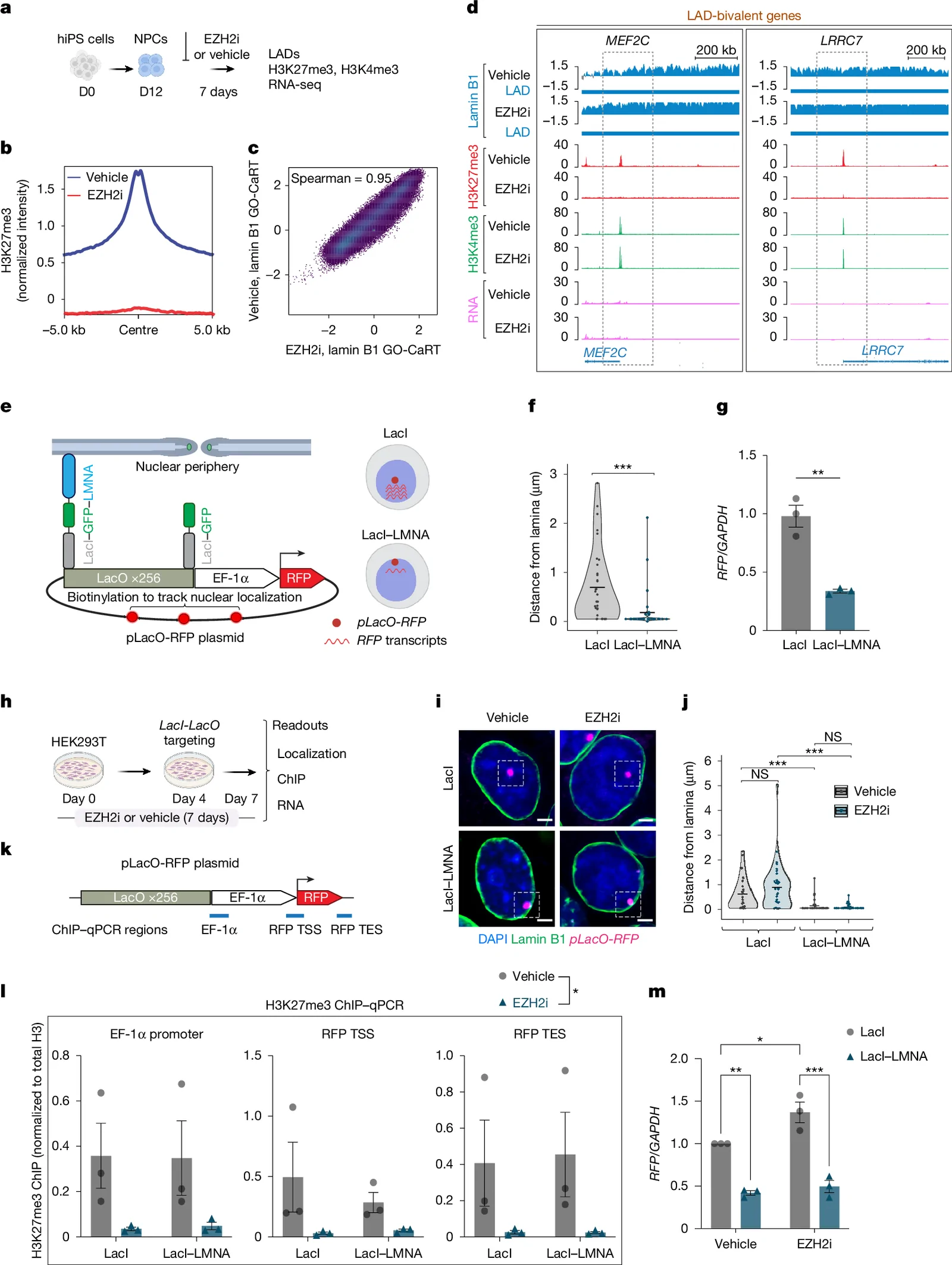
Lamina association exerts transcriptional repression independent of H3K27me3. **a**, Schematic of EZH2 inhibition in NPCs. **b**, Normalized H3K27me3 levels (at H3K27me3 peaks in vehicle-treated NPCs) in NPCs treated with vehicle or EZH2i for 7 days. **c**, Genome-wide lamin B1 signal between vehicle and EZH2i-treated NPCs. **d**, GO-CaRT, CUT&RUN and RNA-seq tracks at example LAD bivalent genes in vehicle and EZH2i-treated NPCs. The dashed boxes show loss of H3K27me3 peaks with no apparent change in transcription. **e**, Schematic of the *LacI-LacO* system for targeting plasmid DNA reporters to the lamina. **f**, Quantification of biotinylated *pLacO-RFP* distance from the lamina. *n* = 30–40 cells per condition. **g**, RFP reporter expression (*n* = 3 independent biological replicates) in LacI or LacI–LMNA-expressing cells. *P* values in **f** and **g** were calculated using two-tailed *t*-tests. **h**, Schematic of EZH2 inhibition in *LacI-LacO*-expressing HEK293T cells. **i**,**j**, Representative images and quantification of the distance of biotinylated *pLacO-RFP* (red) from the lamina (LMNB1, green) in LacI or LacI–LMNA-expressing cells treated with vehicle or EZH2i (5 µM) as in **h**. *n* = 30–40 cells per condition. *P* values were calculated using two-way analysis of variance (ANOVA) with Tukey’s post hoc test. Scale bars, 3 µm. **k**, Schematic of the *pLacO-RFP* plasmid; ChIP–quantitative PCR (qPCR) amplicons are indicated in blue. **l**, H3K27me3 ChIP–qPCR at the three plasmid amplicons in **k** in vehicle or EZH2i-treated cells expressing LacI or LacI–LMNA as in **h**. The H3K27me3 ChIP signal was normalized to the total H3 ChIP signal in each sample. *n* = 3 independent biological replicates. *P* values were calculated using two-way ANOVA. **m**, RFP reporter expression in cells expressing LacI or LacI–LMNA treated with vehicle or EZH2i as in **h**. *n* = 3 independent biological replicates. *P* values were calculated using two-way ANOVA with Tukey’s post hoc test. For **g**, **l** and **m**, data are mean ± s.e.m. The diagrams in **a**, **e** and **h** were created in BioRender; Hamid, S. https://biorender.com/jw25feh (2026).

Removal of H3K27me3 resolves bivalent chromatin to the H3K4me3-monovalent state, which theoretically results in increased transcription. However, the observed increase in gene expression after H3K27me3 loss is highly variable, and many bivalent genes are not induced after resolution of bivalency to H3K4me3 monovalency^20,23^. We hypothesized that the lamina produces a transcriptionally repressive state independent of local levels of H3K27me3. To test this concept, we treated NPCs with tazemetostat (EPZ-6438), a potent and selective inhibitor of EZH2 (EZH2i)—the enzyme that catalyses H3K27me3—to globally reduce H3K27me3 levels (Fig. 4a). Compared to a DMSO vehicle control, treatment of NPCs for 1 week with EZH2i resulted in a near total loss of H3K27me3 peaks (Fig. 4b).

Lamin B1 GO-CaRT profiles of vehicle and EZH2i-treated NPCs were highly similar, indicating that EZH2 inhibition does not grossly disrupt NPC LAD architecture (Fig. 4c). At bivalent genes, lamin B1 enrichment was also similar between vehicle and EZH2i-treated NPCs (Extended Data Fig. 9g). Although EZH2 inhibition derepressed formerly bivalent genes genome-wide, expression of those in LADs remained significantly lower than bivalent genes in non-LAD regions of vehicle-treated cells (Extended Data Fig. 9h). For example, *MEF2C* and *LRRC7* remained in LADs and were expressed at low levels after EZH2 inhibition (Fig. 4d). These results indicate that localization of bivalent genes to the lamina is not dependent on H3K27me3 and that their lowly expressed state is reinforced by the repressive environment of the lamina.

## LAD location is sufficient for repression

To more directly examine the interplay between spatial compartmentalization and H3K27me3 deposition in transcriptional repression at the lamina, we engineered a system to tether plasmid DNA reporters to the lamina. This system exploits the interaction between the *Escherichia coli* DNA-binding Lac repressor (LacI) protein and its cognate Lac operator (LacO) binding sites^29^. We generated a LacI–lamin A (LacI–LMNA) fusion protein to tether plasmid DNA containing an array of LacO sequences and an EF-1α promoter-driven red fluorescent protein (*RFP*) reporter gene (*pLacO-RFP*; Fig. 4e). Immunostaining in HEK293T cells confirmed that the LacI–LMNA fusion protein localized to the nuclear periphery, whereas the LacI-only protein was present throughout the nucleus (Extended Data Fig. 9i).

To confirm successful plasmid tethering by LacI, we biotinylated *pLacO-RFP* and visualized its subnuclear distribution through fluorescent streptavidin staining. In cells expressing LacI alone, *pLacO-RFP* was distributed throughout the nucleus (Fig. 4f). By contrast, in cells expressing LacI–LMNA, *pLacO-RFP* was recruited to the nuclear periphery and *RFP* expression was strongly repressed relative to the LacI control (Fig. 4f,g).

We next examined whether H3K27me3 contributes to transcriptional repression induced by lamina tethering. We treated HEK293T cells with EZH2i and confirmed loss of H3K27me3 by western blotting (Fig. 4h and Extended Data Fig. 9j, Supplementary Fig. 1). EZH2 inhibition did not affect *pLacO-RFP* localization to the nuclear lamina by LacI–LMNA (Fig. 4i,j). ChIP demonstrated that the plasmid reporter DNA was chromatinized and marked by H3K27me3 in vehicle-treated cells and that H3K27me3 was effectively depleted by EZH2 inhibition (Fig. 4k,l). In cells expressing LacI, EZH2 inhibition increased *RFP* reporter expression (Fig. 4m). By contrast, EZH2 inhibition did not derepress *RFP* expression in cells expressing LacI–LMNA (Fig. 4m). These findings demonstrate that localization of DNA to the nuclear lamina represses transcription independently of H3K27me3.

## Pol II cofactors are excluded from the lamina

To further dissect the transcriptionally repressive environment of the lamina, we considered the possibility that lamina-associated transcriptional repression involves spatial segregation of active RNA polymerase II (Pol II) and its regulators^30^. We profiled the genome-wide occupancy of total Pol II, its initiating form (C-terminal domain repeat Ser5-phosphorylated, Pol II-Ser5P) and elongating form (Ser2-phosphorylated, Pol II-Ser2P) during GW17 cortical neurogenesis.

**Fig. 5:**
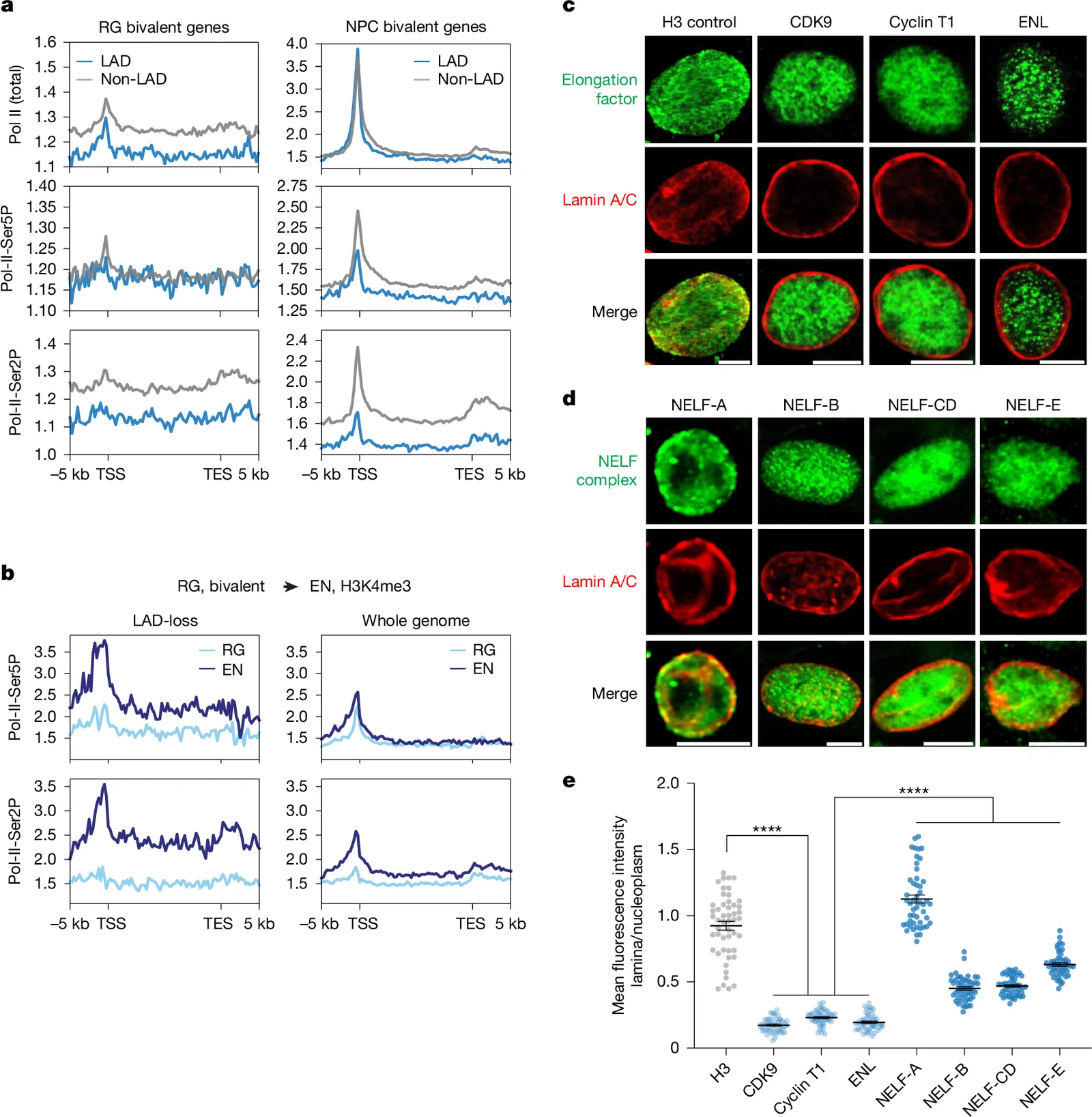
Spatial segregation of Pol II regulators limits transcription of bivalent genes at the lamina. **a**, Average IgG-normalized total Pol II (top), Pol-II-Ser5P (middle) and Pol-II-Ser2P (bottom) CUT&RUN signal at LAD (blue) and non-LAD (grey) bivalent genes in RGs (left) and NPCs (right). **b**, Average Pol-II-Ser5P (top) and Pol-II-Ser2P (bottom) signal at genes resolving from bivalent in RGs to H3K4me3-monovalent in ENs in LAD-loss regions (left) and the whole genome (right). Light blue, RGs; dark blue, ENs. **c**–**e** Representative immunocytochemical micrographs (**c**,**d**) and quantification (**e**) of the subnuclear distribution of elongation factors (**c**) and NELF complex components (**d**) with respect to the lamina (marked by lamin A/C, red) in GW20 cortical cells. Lamina enrichment ratios in **e** were calculated by measuring the ratio of elongation factor/NELF mean fluorescence intensity within a region of interest defined by lamin A/C staining at the nuclear periphery to the intensity within a region of interest encompassing the entire nuclear interior of the same cell. Scale bars, 5 μm (**c**,**d**). *n* = 50 cells per antibody from one representative experiment, which was performed twice with similar results. *P* values were calculated using one-way ANOVA with Tukey’s post hoc test. Data are mean ± s.e.m. ****P < 0.0001.

**Extended Data Fig. 10:**
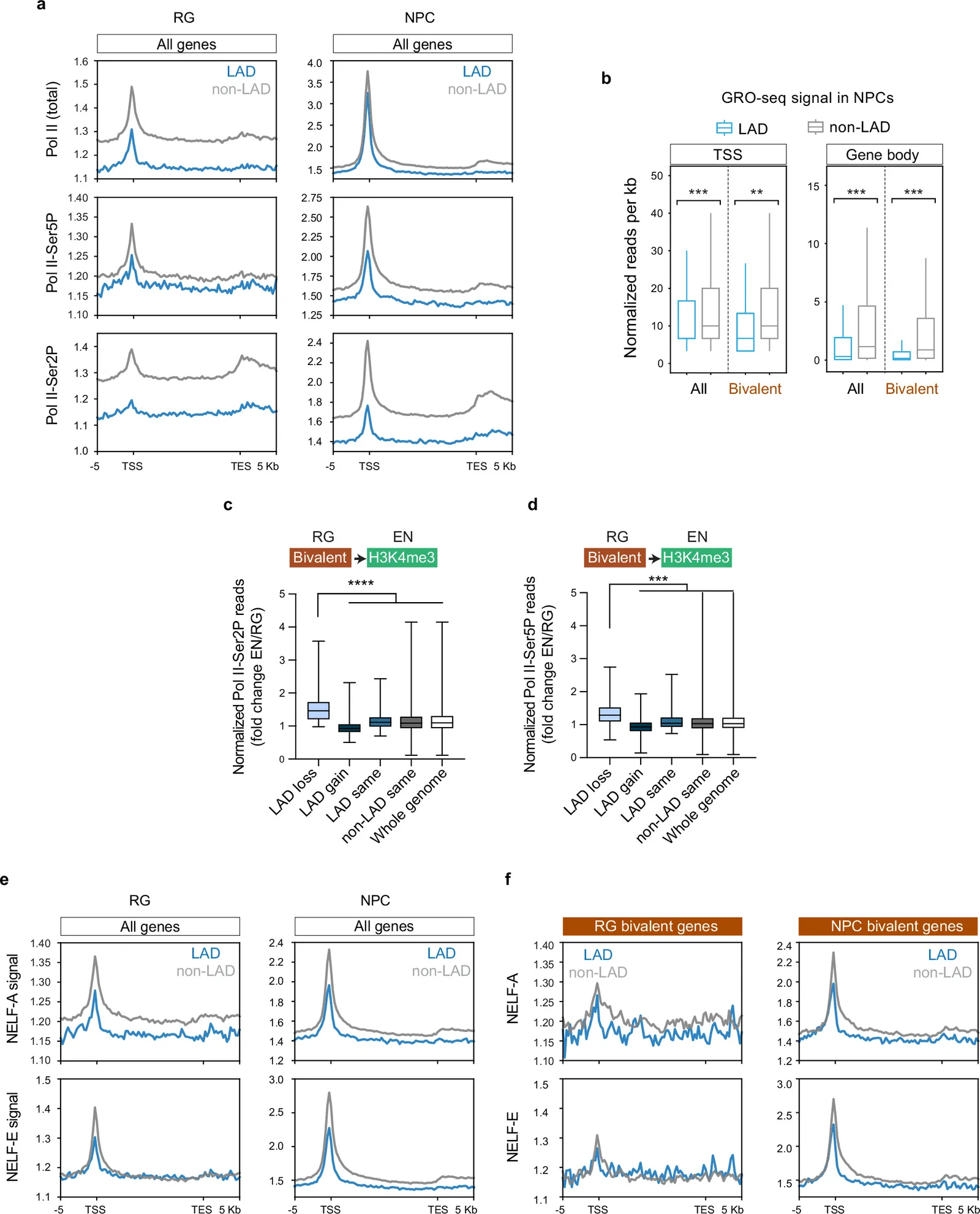
Pol II and NELF dynamics during neurogenesis. **a**, Metagene plots of IgG-normalized total Pol II (top) Pol II-Ser5P (middle) and Pol II-Ser2P (bottom) signal at genes in LADs and non-LADs in RG (left) and NPC (right). **b**, Normalized LAD (blue) and non-LAD (grey) gene GRO-seq signal in NPCs (from Barbieri et al.^31^) at TSS (left) and gene body (right). Boxes show interquartile range with line at median; whiskers extend 1.5x interquartile range from bounds of box. In order of graph bars, n = 731, 5042, 148, 1300 genes. Identical gene lists are plotted for TSS and gene body plots. *p*-values calculated by two-sided continuity-corrected Wilcoxon rank sum test with Benjamini-Hochberg correction. Boxes denote interquartile range with line at median; whiskers extend 1.5x interquartile range from bounds of box. **c, d**, Fold change in Pol II-Ser2P (**c**) and Pol II-Ser5P (**d**) signal at bivalent genes that resolve to the H3K4me3-monovalent state in iN (n = 78 genes in LAD loss, 87 [LAD gain], 100 [LAD same], 672 [non-LAD same], 937 [WG]). Boxes denote interquartile range with line at median; whiskers show min/max. *p*-values calculated by two-way ANOVA with Tukey’s post-hoc test. **e**, Average IgG-normalized NELF-A (top) and NELF-E (bottom) CUT&RUN signal at LAD (blue) and non-LAD (grey) genes in RG (left) and NPC (right). **f**, Average IgG-normalized NELF-A (top) and NELF-E (bottom) CUT&RUN signal at LAD (blue) and non-LAD (grey) bivalent genes in RG (left) and NPC (right).

In RGs, genes located within LADs exhibited globally lower levels of all three forms of Pol II than genes located outside LADs (Extended Data Fig. 10a). iPS-cell-derived NPCs exhibited a similar depletion of Pol II across LAD genes relative to non-LAD genes (Extended Data Fig. 10a). Bivalent genes within RG and NPC LADs showed markedly reduced occupancy of elongating Pol II Ser2P across gene bodies compared with non-LAD bivalent genes, while total Pol II and Pol II-Ser5P were present at bivalent promotors regardless of LAD status (Fig. 5a). Notably, in both RGs and NPCs, depletion of Pol II at LAD genes was most substantial for the elongating Pol II-Ser2P form (Fig. 5a and Extended Data Fig. 10a). Analysis of global run-on sequencing (GRO-seq) data from iPS-cell-derived NPCs^31^ revealed significantly lower nascent transcription at LAD bivalent genes, particularly across gene bodies, compared with non-LAD bivalent genes (Extended Data Fig. 10b). These findings indicate that lamina proximity preferentially restricts transcriptional elongation rather than initial Pol II recruitment.

We next assessed bivalent genes that resolve to H3K4me3 monovalency during neurogenesis. Compared to bivalent genes genome-wide, bivalent genes in LAD-loss regions showed a greater increase in Pol II enrichment after resolution to H3K4me3 monovalency in ENs, particularly for the elongating Ser2P form across the gene body (Fig. 5b and Extended Data Fig. 10c,d). These data suggest that release from the lamina facilitates transcriptional elongation at neurogenesis-induced genes.

To investigate whether the local environment of the lamina excludes the molecular machinery necessary for productive transcription, we analysed the subnuclear localization of Pol II regulatory cofactors involved in elongation. CDK9 and cyclin T1—core components of the positive transcription elongation factor P-TEFb—and ENL, a subunit of the super elongation complex, were depleted from the nuclear periphery and enriched in the interior (Fig. 5c,e). By contrast, subunits of the negative elongation factor complex (NELF-A, NELF-B, NELF-C/D and NELF-E), which promote promoter-proximal transcriptional pausing, were comparatively enriched at the lamina (Fig. 5d,e). Genome-wide profiling demonstrated binding of NELF-A and NELF-E at promoters in both LADs and non-LADs (Extended Data Fig. 10e,f), further indicating the presence of pausing machinery at lamina-associated bivalent promoters.

Together, these findings support a model in which the nuclear lamina serves as a repressive environment by spatially excluding proteins essential for productive transcription, while permitting Pol II recruitment and pausing. Repositioning bivalent genes away from the lamina during neuronal differentiation relieves this elongation constraint, enabling transcriptional activation of lineage-specific gene programs.

## Discussion

By studying LAD and SPAD dynamics during human cortical development, we identified an essential interplay between spatial genome organization and the bivalent state of neurodevelopmental genes. We found that the repressive environment of the lamina maintains the poised state of bivalent genes independent of H3K27me3. This repression of lamina-associated bivalent genes instead corresponds to differences in subnuclear enrichment of key transcriptional regulators. Together, these results establish the importance of subnuclear environments in shaping the transcriptional output of local chromatin states (Supplementary Fig. 2).

In human tissues, cellular differentiation takes place in a complex tissue microenvironment that is not fully recapitulated in cell culture. To understand the physiological significance of subnuclear compartment dynamics, we developed high-resolution LAD and SPAD maps of a key developmental lineage isolated directly from the developing human brain. To do this, we overcame technical challenges that limited previous studies mostly to cell culture. This pursuit yielded methods that will be broadly useful for studies of chromatin organization in defined cell types within tissues. More importantly, gathering data from cells developing in the in vivo tissue context revealed previously unappreciated spatial genomic dynamics.

During human cortical neurogenesis, LAD remodelling was extensive, affecting around 23% of the genome. Such large-scale changes in LAD organization have not been previously observed in in vitro differentiation models. For example, most LADs remain stable when mouse embryonic stem cells differentiate into NPCs or astrocytes^32^ (only 5–10% of the genome is remodelled) or myoblasts into myotubes^33^ (around 3% of the genome), or embryonic stem cells into cardiomyocytes^34^. These data suggest that the in vivo niche contributes importantly to LAD reorganization. This aligns with findings from mouse preimplantation embryogenesis^35^. These results nonetheless underscore the importance of studying genome architecture dynamics in cells that develop in vivo. Notably, LAD reorganization in our in vitro neurogenesis model was of comparable magnitude to in vivo changes, albeit less pronounced (23% of the genome in vivo versus 19% in vitro), suggesting that differentiation of postmitotic neurons involves uniquely extensive LAD remodelling.

Although nuclear speckles have long been proposed as hubs for active genes^4,11,12^, genome-wide analysis was only recently enabled in cell culture by tyramide signal amplification sequencing^14,36^ and in human tissue by GO-CaRT^7^. However, study of the movement of genomic regions from the lamina to speckles was lacking. Our data indicate that the relocation of genomic regions from the nuclear lamina to nuclear speckles is a key aspect of human brain development. Of the 739 genes that detach from the lamina during neurogenesis, nearly half became associated with nuclear speckles. While the molecular mechanisms driving LAD-to-SPAD translocation remain unclear, developmentally important transcription factors may guide genes to speckles^37^. Together, these findings suggest that LAD-to-SPAD switching is a key component of lineage specification and neuronal differentiation in the developing brain.

Our work goes beyond the linear analysis of chromatin state, showing how spatial genome organization regulates the activity and transcriptional output of bivalent chromatin. Our finding that lamina-associated bivalent genes are expressed at much lower levels than those in the rest of the genome suggests that the local environment of the lamina contributes importantly to transcriptional repression. This spatial segregation of specific genomic regions may be particularly important to prevent untimely activation of developmental genes, thereby ensuring fidelity of lineage progression. Consistent with this notion, during neurogenesis, bivalent genes that resolve to H3K4me3 monovalency but remain lamina-associated are only moderately induced, while those that detach from the lamina exhibit a threefold to eightfold increase in transcription. Notably, loss of lamina association is sufficient to upregulate even genes that retain bivalency during in vitro neurogenesis. These findings support the idea that lamina proximity confers a low-expression, poised transcriptional state to genes marked by bivalent chromatin in stem/progenitor cells. Moreover, these observations provide a potential explanation for the previously observed variability in transcriptional induction after bivalency resolution.

The lowly expressed, poised state of bivalent genes in LADs could be due to the repressive nature of H3K27me3 (refs. ^7,17,38^) and/or to proximity to the lamina. Our pharmacological experiments indicate that lamina-associated bivalent genes are repressed independently of H3K27me3. Moreover, depletion of H3K27me3 increased the expression of heterologous plasmid reporters located in the nuclear interior but did not upregulate gene expression from lamina-tethered plasmids. Consistent with our findings that H3K27me3 does not dictate lamina-associated transcriptional repression, recent work in mouse preimplantation embryos identified an antagonistic relationship between lamina association and H3K27me3 (ref. ^39^). These data establish a model in which spatial proximity to the nuclear lamina is a key driver of the poised state of bivalent developmental genes.

Lastly, we show that lamina-associated repression involves the spatial segregation of transcriptional machinery away from the nuclear periphery. Multiple transcriptional elongation factors were depleted at the lamina, whereas the NELF complex, a negative regulator of elongation, was relatively enriched at the nuclear periphery. These observations correspond to the lack of Pol II elongation at bivalent LAD genes in RGs and the strong increase in activated Pol II at these genes after their detachment from the lamina in ENs. Thus, the environment of the lamina may contribute to the poised state of bivalent genes by restraining their transition to transcriptional elongation. Notably, a recent genome-wide screen for repressors of lamina-associated transcription identified many negative regulators of elongation^40^. Together, these findings imply the existence of a spatial barrier between the lamina and adjacent nucleoplasm, and that the movement of bivalent genes across this barrier is a key aspect of developmental gene regulation. Future studies delineating the molecular and biophysical processes responsible for this exclusion of transcriptional machinery will clarify our understanding of this important aspect of spatial epigenomic regulation. More broadly, our findings reveal a critical role for spatial segregation of genomic loci and transcriptional machinery in shaping the transcriptional output of specific chromatin states during development.

## Methods

### Tissue collection and processing

Human brain tissue samples (GW16–20) were collected from de-identified donors with previous patient consent in strict observance of legal and institutional ethical regulations. All protocols were approved by the Human Gamete, Embryo and Stem Cell Research Committee (GESCRC) and Institutional Review Board (IRB) at the University of California, San Francisco (UCSF). Primary human brain tissue was collected and processed as previously described^41^. In brief, cortical tissue was collected in artificial cerebrospinal fluid (ACSF) containing 125 mM NaCl, 2.5 mM KCl, 1 mM MgCl_2_, 1 mM CaCl_2_ and 1.25 mM NaH_2_PO_4_ under a stereotaxic dissection microscope (Leica). Tissue samples were cut into small pieces and snap-frozen by placing the tissue onto a strip of aluminium foil in prechilled 2-methylbutane on dry ice for around 10 min. The samples were stored in cryovials at −80 °C. For individual germinal zone and cortical plate cultures, the samples were dissected in artificial CSF to separate germinal zone from the cortical plate before dissociation. Tissue pieces were then placed into a prewarmed (37 °C) solution of papain (Worthington) and incubated at 37 °C. After incubation for approximately 60 min, tissue was triturated according to the manufacturer’s protocol, and the samples were spun through an ovomucoid gradient to remove debris. The dissociation medium was removed, and cells were resuspended in NES medium (DMEM/F12 (Gibco), 1:1,000; B27 (Invitrogen), 1:100; N2 (Life Technologies); 20 ng ml^−1^ FGF (PeproTech); 20 ng ml^−1^ EGF (PeproTech); 20 µg ml^−1^ insulin (Thermo Fisher Scientific); 5 ng ml^−1^ of BDNF (PeproTech); and 10 µM ROCK inhibitor Y-27632 (Selleckchem)). Cells were plated in a Matrigel-coated (1 µg ml^−1^, Corning) eight-well chambered coverglass (Thermo Fisher Scientific) and allowed to attach for 24 h at 37 °C.

### Maintenance, neural induction and neuronal differentiation of iPS cells

The hiPS cell line WTC11 (ref. ^42^) was obtained from WiCell Stem Cell bank and authenticated by short-tandem-repeat profiling at the source. Cells were maintained in StemFlex medium (Gibco) on plates coated with Matrigel (Corning) and passaged as clumps with ReLeSR (StemCell Technologies). Differentiation of iPS cells was performed as described previously^11^ with modifications. For neural induction of iPS cells into NPCs, cells were dissociated with Accutase (Gibco) and seeded on Matrigel-coated plates at a density of 150,000 cells per cm^2^ in StemFlex supplemented with 10 µM Y-27632. The next day (day 0), cells were washed in PBS and fed with neural induction medium (NIM, DMEM/F12 GlutaMax (Gibco) containing 1% ITS-G (Gibco) and 200 µM l-ascorbic acid 2-phosphate (Santa Cruz)). On days 0–2, cells were fed daily with NIM supplemented with 100 nM LDN-193189 (SelleckChem), 10 µM SB431542 (Selleckchem) and 2 µM XAV939 (Cayman). On days 3–9, NIM was supplemented with LDN-193189 and SB431542 only. From day 10 to day 19, cells were fed daily with NPC maturation medium (1:1 mixture of DMEM/F12 GlutaMax and Neurobasal-A (Gibco) supplemented with 1% N-2 and 2% B-27 minus vitamin A (Gibco)). On day 20, NPCs were either collected for analysis or further differentiated into neurons. For neuronal differentiation, NPCs were dissociated with Accutase and seeded at a density of 275,000 cells per cm^2^ on plates coated with 100 µg ml^−1^ poly-l-ornithine, 100 µg ml^−1^ poly-d-lysine, 10 µg ml^−1^ laminin and 10 µg ml^−1^ fibronectin (Sigma-Aldrich) in neuronal differentiation medium (Neurobasal-A supplemented with 2% B-27, 2 mM l-glutamine (Gibco), 200 µM l-ascorbic acid 2-phosphate, 10 µM dibutyryl-cyclic AMP (Sigma-Aldrich), 10 ng ml^−1^ BDNF (Peprotech), 10 ng ml^−1^ GDNF (Peprotech) and 10 µM DAPT (MedChemExpress)). Cells were fed by 50% medium exchange every 2 days and collected for downstream analysis after 1 week of neuronal differentiation.

### EZH2 inhibition in NPCs

On day 10, iPS-cell-derived NPCs were passaged using Accutase and plated onto Matrigel-coated six-well plates in NPC maturation medium supplemented with 10 µM ROCKi. On days 11–12, cells were fed with NPC maturation medium. On day 13, the medium was removed and fresh medium containing EZH2 inhibitor tazemetostat (EPZ-6438, Selleckchem, 1 μM) or DMSO was added to the cells. Cells were fed fresh medium containing the inhibitor (or DMSO) every day for 1 week, at which point they were collected for downstream experimental analyses.

### Nucleus isolation and FANS

Snap-frozen cortical tissue was cut into small pieces and transferred to a prechilled 7 ml Dounce Tissue Grinder (Wheaton) containing 5 ml nucleus extraction buffer (NEB: 10 mM HEPES pH 7.4, 25 mM KCl, 5 mM MgCl_2_, 0.25 M sucrose, 0.1% Triton X-100, 1× Halt protease inhibitor cocktail (Thermo Fisher Scientific)). RiboLock (Thermo Fisher Scientific) was added to all the buffers to preserve RNA integrity. While still on ice, the tissue was dissociated with 5–6 strokes with loose pestle A followed by 8–10 strokes with tight pestle B, until no tissue pieces were visible. The sample was incubated on ice for 5 min after which it was transferred to a prechilled 15-ml conical tube. The sample was centrifuged at 500*g* for 10 min at 4 °C. The supernatant was removed, and the nuclear pellet was resuspended in 10 ml NEB without Triton X-100. The homogenate was passed through a 40 μm strainer into a 50 ml conical tube. Formaldehyde was added to a final concentration of 0.1% and the sample was incubated for 2 min at room temperature with rotation. Glycine was added to a final concentration of 75 mM to quench the reaction. BSA was added to a final concentration of 1% and the sample was centrifuged at 500*g* for 10 min at 4 °C. The supernatant was discarded, and the nuclear pellet was resuspended in 1 ml staining buffer (PBS containing 1% BSA). The nuclei were filtered through a 40 µm strainer into a 1.5 ml low-bind microcentrifuge tube and counted under a microscope. The nuclei were pelleted at 500*g* for 10 min at 4 °C and resuspended in 100–150 μl of staining buffer. Antibody staining was carried out for 1 h at 4 °C with rotation using the following antibodies: PAX6-Alexa Fluor 488 (BD Biosciences, 1:20), EOMES-PE-Cy7 (Invitrogen, 1:20) and SATB2-Alexa Fluor 647 (Abcam, 1:100). After staining, 900 μl of staining buffer was added and the samples were centrifuged at 500*g* for 10 min at 4 °C. The nuclear pellet was resuspended in 1 ml of staining buffer and centrifuged at 500*g* for 10 min at 4 °C. Nuclei were resuspended in 1–2 ml of staining buffer (depending on the starting material and yield) and filtered into a 70 µm mesh FACS tube (BD). DAPI was added at 1 μg ml^−1^ just before FANS. AbC Total Compensation capture beads (Thermo Fisher Scientific) were used for generating single-colour compensation controls. FANS was conducted on BD FACS Aria II Cytometer using a 70 μm nozzle. Sorted nuclei were collected in 5 ml tubes containing 300–500 μl of collection buffer (PBS containing 5% BSA and RNasin Plus RNase inhibitor (Promega)). Sorted nuclei were collected by centrifuging at 500*g* for 10 min at 4 °C and processed for downstream analyses (RNA-seq, Fab blocking and GO-CaRT/CUT&RUN).

### Development of Fab blocking for antibody-based chromatin profiling

HEK293T cells were obtained from ATCC and authenticated at source. Cells were collected by trypsinization. Cells were fixed in 0.1% formaldehyde for 2 min at room temperature, followed by quenching in 0.1 M glycine (prepared in wash buffer). Cells were resuspended in 1 ml wash buffer (20 mM HEPES-KOH pH 7.5, 150 mM NaCl, 0.1% BSA, 0.5 mM spermidine and 1× Halt protease inhibitor cocktail) and centrifuged at 300*g* for 5 min. This step was repeated for a total of two washes. Cells were resuspended in wash buffer and aliquoted at 100 μl per tube into 0.5 ml PCR tubes (around 100,000 cells per experimental condition). Next, 8 μl of BioMagPlus concanavalin A beads (Polysciences) activated in binding buffer (20 mM HEPES-KOH pH 7.9, 10 mM KCl, 1 mM CaCl_2_ and 1 mM MnCl_2_) were added to each sample and rotated on a nutator for 10 min at room temperature. The cells were placed onto a magnet to clear and the liquid was removed. Cells bound to beads were resuspended in 50 μl cell permeabilization buffer (20 mM HEPES pH 7.5, 0.1 mM CaCl_2_, 3 mM MgCl_2_, 100 mM KCl, 0.05% digitonin (Sigma-Aldrich) and 1× Halt protease inhibitor cocktail) and incubated for 30 min at room temperature on a nutator. The supernatant was removed, and the beads were resuspended in 50 μl of antibody binding buffer (wash buffer containing 2 mM EDTA and 0.025% digitonin) containing an antibody against H3K27me3 (Cell Signaling Technologies, 1:100). IgG (Cell Signaling Technologies, 1:100) was included in parallel as a negative control. The samples were incubated overnight at 4 °C on a nutator. The next day, the supernatant was removed, and the beads were washed twice with 200 μl wash buffer containing 0.025% digitonin (Wash-Dig). The beads were resuspended in 50 μl of Wash-Dig containing monovalent anti-rabbit Fab fragments (Jackson Immuno) and incubated for 30 min at 4 °C. We tested the following Fab dilutions: 1:20, 1:50, 1:100, 1:250, 1:500, 1:1,000, 1:10,000, 1:50,000 and 1:100,000. We also tested three incubation times: 5 min, 15 min and 30 min. Dilutions of 1:250 and lower and incubation times of 5 min and below did not result in efficient blocking by the Fab fragments. After two washes with WaB-Dig (wash buffer containing 0.025% digitonin), the beads were resuspended in 50 μl of WaB-Dig containing pA/G-MNase (purified from Addgene plasmid 123461 at Macro Lab UC Berkley) and nutated at 4 °C for 1 h. The supernatant was removed, and the beads were washed twice with WaB-Dig. The beads were resuspended in 100 μl of WaB-Dig containing 2 mM CaCl_2_ to activate pA/G-MNase. The digestion was carried out for 30 min in a chilled metal block on ice. Digestion was stopped by adding 100 μl of 2× STOP (200 mM NaCl, 20 mM EDTA, 4 mM EGTA, 50 μg ml^−1^ RNase A, 40 μg ml^−1^ glycogen and 10 pg ml^−1^ heterologous DNA). The samples were incubated at 37 °C for 30 min to release pA/G-MNase-cleaved fragments. The contents of the tube were transferred to 1.5 ml Eppendorf tubes containing 2 μl each of 10% SDS and proteinase K (20 mg ml^−1^). The samples were incubated at 55 °C for 1 h to reverse cross-linking. DNA was extracted using the phenol–chloroform method. Purified DNA fragments were analysed by TapeStation High Sensitivity D1000 assay (Agilent).

To demonstrate that Fab blocking allows subsequent binding of another antibody for GO-CaRT/CUT&RUN analyses, we incubated the Fab-blocked H3K27me3 samples with an antibody against lamin B1. In brief, after Fab blocking of H3K27me3, the beads were resuspended in 50 μl of WaB-Dig containing lamin B1 antibody (Abcam, 1:100). The sample was nutated at 4 °C for 2 h. After two washes with WaB-Dig, the steps of pA/G-MNase binding, digestion and DNA extraction were carried out as described above. Purified DNA fragments were analysed by TapeStation High Sensitivity D1000 (Agilent).

### Fab blocking followed by GO-CaRT and CUT&RUN analyses

Nuclei sorted by FANS were resuspended in PBS containing 1% BSA and divided into 100 μl aliquots in 0.5 ml PCR tubes. Next, 8 μl of activated BioMagPlus concanavalin A beads were added to each sample and rotated for 10 min at room temperature. The samples were placed onto a magnetic stand to remove the liquid. To block antibodies used in FANS, nuclei bound to concanavalin A beads were resuspended in 50 μl wash buffer (20 mM HEPES-KOH pH 7.5, 150 mM NaCl, 0.1% BSA, 0.5 mM spermidine and 1× Halt protease inhibitor cocktail) containing anti-rabbit and anti-mouse monovalent Fab fragments (Jackson Immuno) at 1:30. The samples were nutated at 4 °C for 30 min. The samples were then placed onto a magnetic stand to remove the liquid. The beads were resuspended in 200 μl wash buffer to remove excess Fab fragments. The beads were resuspended in 50 μl antibody binding buffer (wash buffer containing 2 mM EDTA) containing primary antibody of interest for GO-CaRT/CUT&RUN. Antibody incubation was carried out overnight on a nutator at 4 °C. The next day, the samples were briefly spun and placed onto a magnetic stand to remove the liquid. The samples were washed twice with 200 μl wash buffer. The beads were resuspended in 50 μl wash buffer containing pA/G-MNase (3 µg ml^−1^) and rotated for 1 h at 4 °C. The samples were briefly spun and placed onto a magnetic stand to remove the liquid. The samples were washed twice with 200 μl wash buffer. The beads were gently resuspended in a 100 μl wash buffer containing 2 mM CaCl_2_ (to activate pA/G-MNase) and placed in a prechilled metal block on ice. Digestion was carried out for 30 min and stopped by adding 100 μl of 2× STOP (200 mM NaCl, 20 mM EDTA, 4 mM EGTA, 50 μg ml^−1^ RNase A, 40 μg ml^−1^ glycogen and 10 pg ml^−1^ heterologous DNA). The samples were then incubated at 37 °C for 20 min to release pA/G-MNase cleaved fragments. Next, 2 μl SDS (10%) and 2 μl proteinase K (20 mg ml^−1^) were added to each sample and the sample was incubated at 55 °C for 1 h. DNA was extracted using the phenol–chloroform method. Purified DNA fragments were analysed using the TapeStation High Sensitivity D1000 assay (Agilent).

### Lamin B1 GO-CaRT–seqChIP

iPS-cell-derived NPCs were collected with Accutase and the cell pellet was resuspended in 1 ml ice-cold PBS. The cells were centrifuged at 500*g* for 3 min at 4 °C and the cell pellet was resuspended in 1 ml of nucleus-isolation buffer (NIB: 10 mM HEPES-KOH pH 7.9, 10 mM KCl, 0.1% NP-40, 0.5 mM spermidine and 1× Halt protease inhibitor cocktail) and incubated for 10 min on ice. The nuclear pellet was collected by centrifuging at 600*g* for 3 min at 4 °C and again resuspended in 1 ml of NIB to wash. The nuclear pellet was collected by centrifugation at 600*g* for 3 min and resuspended in 100 μl of NIB.

Lamin B1 GO-CaRT was performed as described above, and lamin-B1-enriched chromatin released into the soluble fraction was used for subsequent sequential (two-step) ChIP. Lamin B1 GO-CaRT was performed in twelve 0.5 ml PCR tubes each containing around 500,000 nuclei (total of around 6 million nuclei) in 100 μl of NIB each. For the antibody and pA/G-MNase binding steps, the volumes were scaled up from 50 μl to 100 μl. The reaction was stopped using 2× STOP containing lamin B1 blocking peptide (Abcam) at 15 μg ml^−1^ followed by incubation at 37 °C for 15 min. The samples from 12 PCR tubes were pooled together and centrifuged at 14,000*g* for 5 min at 4 °C. The supernatant containing lamin-B1-associated DNA fragments was divided into 300 μl aliquots for the first ChIP. One aliquot was kept as input and stored at −20 °C for later use. For each ChIP, we used lamin B1 GO-CaRT supernatant from around 750,000 nuclei. The samples were incubated with antibodies against lamin B1 (1:100), H3K4me3 (1:100), H3K27me3 (1:50) and IgG control (1:100) at 4 °C overnight with rotation. The next day, protein A Dynabeads (Thermo Fisher Scientific) were equilibrated by washing twice in 1 ml wash buffer containing 0.05% Tween-20 (wash + Tween). The beads were resuspended in wash + Tween in the original volume of beads. Then, 15 μl of equilibrated beads was added to each ChIP reaction and the samples were incubated at 4 °C for 1 h. The samples were washed twice with 1 ml wash + Tween by rotating for 5 min at room temperature. The tubes were placed on the magnet to remove the supernatant. At this point, the controls for the first ChIP (lamin B1 GO-CaRT–lamin B1 ChIP and lamin B1 GO-CaRT–IgG ChIP) have finished processing and these samples were resuspended in 200 μl of wash + Tween and stored at −20 °C for DNA extraction later. For H3K4me3 and H3K27me3 first ChIP samples, chromatin was eluted by resuspending the beads in 150 μl of wash + Tween containing the corresponding blocking peptides (10 μg ml^−1^) for H3K4me3 (EpigenTek) and H3K27me3 (EpigenTek), respectively. The samples were incubated at 4 °C for 2 h. For H3K4me3 and H3K27me3, the first ChIP was carried out in two tubes so the total volume of eluted chromatin was 300 μl. The samples were placed on the magnet and the supernatant containing eluted chromatin from the first ChIP was transferred to a new 1.5 ml tube. A 100 μl aliquot of eluted chromatin was saved as the first ChIP input. To the remaining 200 μl of chromatin, an antibody of interest for the second ChIP (H3K27me3 if the first ChIP was done with H3K4me3 or H3K4me3 if the first ChIP was done with H3K27me3) was added and the samples were rotated at 4 °C overnight. The next day, 15 μl of equilibrated protein A Dynabeads were added to each second ChIP sample and incubated at 4 °C for 1 h. The samples were washed twice with 1 ml wash + Tween by rotating for 5 min at room temperature. The tubes were placed on the magnet to remove the supernatant and the beads were resuspended in 200 μl of wash + Tween. To each sample (including samples from first ChIP and inputs), 2 μl of 10% SDS and 2 μl of 20 mg ml^−1^ proteinase K were added. The samples were vortexed and incubated at 55 °C for 1 h. DNA was extracted using the phenol–chloroform method.

### Library preparation and sequencing

Sequencing libraries for GO-CaRT, CUT&RUN and lamin B1-GO-CaRT–ChIP-reChIP were generated using KAPA HyperPrep Kit (Roche) according to the manufacturer’s instructions. The libraries were amplified for 12–14 PCR cycles. DNA fragments were quantified by Tapestation D1000 (Agilent) and Qubit high sensitivity dsDNA (Invitrogen) assays, pooled and sequenced by 150-bp paired-end sequencing on the NovaSeq 6000 or NovaSeq X Plus systems (Illumina).

### CUT&Tag

CUT&Tag in iPS-cell-derived NPCs and neurons was performed as previously described, with modifications^43^. In brief, iPS-cell-derived NPCs and neurons were collected by scraping in 1× NIB, triturated and incubated on ice for 10 min. Cells were then centrifuged at 500*g* for 5 min at 4 °C. Nuclei were resuspended in PBS before fixation with 0.1% formaldehyde for 2 min. Fixation was quenched with 75 mM glycine for 2 min on ice. Nuclei were centrifuged and resuspended in wash buffer 1 (20 mM HEPES pH 7.4, 150 mM NaCl, 0.5 mM spermidine, 1× Halt protease inhibitor cocktail). For each reaction, 100,000 cells were counted for binding to activated concanavalin A magnetic beads and incubated with primary antibodies overnight on a nutator at 4 °C. On day 2, the samples were placed onto a magnetic stand and washed three times with wash buffer 1. The beads were resuspended in 50 µl wash buffer 1 containing secondary antibodies at a 1:100 dilution and nutated for 1 h at 4 °C. After clearing on a magnetic stand, the beads were washed twice with wash buffer 1, resuspended in 50 µl wash buffer 2 (20 mM HEPES pH 7.4, 300 mM NaCl, 0.5 mM spermidine, 1× Halt protease inhibitor cocktail) containing protein-A-fused preloaded Tn5 transposase at 1:100 dilution, and nutated for 1 h at 4 °C. The samples were then washed twice with wash buffer 2 and resuspended in 50 µl wash buffer 1 containing 10 mM MgCl_2_ for tagmentation at 37 °C for 1 h. The tagmentation reaction was quenched by addition of 1.7 μl 0.5 M EDTA, 0.5 μl 10% SDS and 0.5 μl 20 mg ml^−1^ proteinase K for 1 h at 55 °C. DNA was phenol–chloroform extracted and resuspended in 1× TE buffer (pH 8.0). DNA was PCR amplified by adding 2 µl each of 10 µM universal i5 primer and uniquely barcoded i7 primer^44^, along with 25 µl NEBNext High-Fidelity 2× PCR Master Mix (NEB). PCR products were size-selected with SPRIselect beads (Beckman Coulter) to recover DNA fragments over 75 bp according to the manufacturer’s instructions. Eluted DNA fragments were quantified using the Tapestation D1000 (Agilent) and Qubit high sensitivity dsDNA (Invitrogen) assays, pooled and sequenced by 150-bp paired-end sequencing on the NovaSeq 6000 system (Illumina).

### GO-CaRT, CUT&RUN and CUT&Tag data processing and domain calling

Sequencing reads were first trimmed to remove adapters and low-quality sequences using Trim Galore (v.0.4.5, https://bioinformatics.babraham.ac.uk/projects/trim_galore) with the default parameters. Trimmed reads were mapped to the human reference genome (hg38) using Bowtie2 (v.2.3.5.1). PCR duplicates were removed using Picard tools (v.1.141, https://broadinstitute.github.io/picard). Post-alignment reads with a mapping quality score of less than 30 were filtered out using SAMtools (v.1.12). For lamin B1/SON, filtered .bam files were downsampled to match IgG read depth. For visualization and further analysis, downsampled .bam files of lamin B1/SON were converted to log_2_-normalized bigwig files using the deepTools (v.3.5.5) bamCompare function with the default parameters with IgG as the control. For visualization of histone modifications, bigwig files were generated using deepTools bamCoverage with the default parameters. Subsequent analysis and visualization of epigenomic data was performed in deepTools, bedtools (v.2.31.0) and ggplot2 (v.4.0.2).

### Domain and peak calling

LADs were called by a two-state hidden Markov model in pomegranate (v.1.1.2) as previously described^45^. The model was called on the median of two biological replicate IgG-normalized bigwig files using a 30 kb bin size. For SPAD calling, bigwig files were converted to bedgraph format. The bedtools map function was then used to calculate average scores across 10 kb bins. Domains were called on the resulting bedgraph using SEACR (v.1.3) in the ‘non stringent’ mode by selecting the top 25% of regions by area under the curve. To ensure robustness of SPAD calls, only regions present in both biological replicates were used for further analysis. LADs/SPADs overlapping with blacklisted regions^46^ were discarded.

H3K4me3 and H3K27me3 peaks were called on the median of two biological replicates using MACS2 (v.2.1.2) with IgG as a control with options ‘--broad --q 0.05’ in addition to the default parameters. Identified peaks were annotated on the basis of the transcription start site (TSS) using Homer (v.4.11). All intersection calculations were performed using bedtools. Promoters were defined as ±1 kb around the TSS of the longest transcript of each gene. Bivalent promoters were those that contained peaks for both H3K4me3 and H3K27me3.

### RNA-seq

Total RNA was extracted from RG, IPC and EN nuclei using the Quick-RNA FFPE RNA extraction kit (Zymo). Total RNA from NPCs and induced neurons was extracted using the Direct-Zol RNA Miniprep kit (Zymo). The samples were depleted of ribosomal RNA (rRNA) using the NEBNext rRNA Depletion kit v2 (NEB). Libraries for sequencing were prepared using the NEBNext Ultra II directional library kit (NEB) according to the manufacturer’s protocol. Libraries were quantified by TapeStation D1000 (Agilent) and Qubit high sensitivity dsDNA (Invitrogen) assays, pooled and sequenced on the NovaSeq 6000 or NovaSeq X Plus systems (Illumina).

### Gene expression analyses

All fastq files were aligned to the reference genome (GENCODE GRCh38) by STAR v.2.7.11b after adaptor trimming. Reads that passed quality control and with read length greater than 50 bp were carried over for downstream counting after mapping to a gene transfer format (GTF) file. Read counts were calculated using featureCounts (v.2.24.0). Transcripts per million values were calculated using RSEM to estimate the absolute expression level for each gene. For heat-map plotting, raw read counts were normalized using DESeq2 (v.1.36.0) to account for sequencing depth and RNA composition between samples. RNA-seq data obtained in this study from GW20 RGs, IPCs and ENs, and GW17 RGs, IPCs and ENs from published datasets^47^, were integrated together into the analysis. The number of annotated transcripts for each gene in LAD, non-LAD/non-SPAD and SPAD was extracted from the Ensembl BioMart database using the biomaRt R package.

### GO analyses

For a given list of genes of interest, GO analysis was performed using clusterProfiler (v.4.4.4) using the default parameters. GO terms with false-discovery rate < 0.05 were designated as significant functional enrichment. GO enrichment analyses was performed using over-representation analysis, corresponding to the one-sided Fisher’s exact test, to identify over-represented functional categories. *P* values were adjusted using the Benjamini–Hochberg procedure. Statistical significance was defined as an adjusted *P* < 0.05 and *q* < 0.05.

### Statistics and data reproducibility

All GO-CaRT and RNA-seq experiments in the human cortex were performed using at least two biological replicates per gestational age. No statistical test was used to predetermine sample size. *P* values were derived from Wilcoxon’s rank-sum tests for genomic and transcriptomic comparisons throughout the study.

### Integration of published GRO-seq data

iPS-cell-derived NPC fastGRO data from a previous study^31^ were obtained from GEO (GSE143844). Raw reads were processed using cutadapt (v.4.4) to remove adapters, low-quality bases and short reads. Cleaned reads were aligned to the hg38 or dm3 reference genome using STAR (v.2.7.11b) in 2-pass mode. Alignment files were normalized to the number of reads uniquely mapped to the *Drosophila* dm3 spike-in genome and converted to strand-specific bigwig files using deepTools (v.3.5.5). For downstream analysis, genes with a total length shorter than 400 bp were excluded. To quantify transcriptional activity, GRO-seq read densities at the TSS (±150 bp) and the gene body (TSS + 300 bp to the transcription end site) were calculated using bedtools (v.2.31.0) and visualized with ggplot2.

### DNA-FISH combined with immunocytochemistry and image analyses

DNA-FISH with immunocytochemistry for lamin B1 and SON was performed as described previously^8^ with slight modifications. In brief, the medium was removed from the chambered coverglass containing dissociated cells from germinal zone and cortical plate and cells were fixed with Histochoice MB (Sigma-Aldrich) for 30 min at room temperature. Cells were washed three times for 10 min each with D-PBS. Cells were permeabilized and blocked in D-PBS containing 5% NGS and 0.5% Triton X-100 for 1 h at room temperature. Cells were incubated in antibody binding solution (5% NGS and 0.1% Triton X-100 in D-PBS) containing lamin B1 or SON antibodies at a 1:500 dilution. The next day, cells were washed three times with D-PBS and incubated with Alexa Fluor 647 anti-rabbit secondary antibody (1:500) for 2 h at room temperature. After three washes with D-PBS, cells were post-fixed in Histochoice MB for 20 min at room temperature. Cells were washed twice with D-PBS, and 0.7% Triton X-100 was included in the third wash to repermeabilize the cells. After a quick wash with D-PBS, cells were treated with an ethanol gradient: 70%, 85% and 100% ethanol each for 2 min at room temperature. The chambered coverglass was allowed to dry at 45 °C until all ethanol evaporated. A humidified chamber was prepared using 50% formamide/2× SSC solution in a dark box and preheated to 83 °C. The DNA-FISH probe (Empire Genomics) mixture (3 µl probe, 17 µl hybridization buffer, 5 µl denaturation buffer (70% formamide, 2× SSC pH 7–8)) was added to the cells and the chambered coverglass was placed in the pre-heated humidified chamber and denatured at 83 °C for 10 min. The samples were transferred to a 37 °C oven and incubated for 16–24 h. The next day, wash solution 1 (0.3% NP-40 and 0.4× SSC) prewarmed to 73 °C was added and incubated for 3 min at room temperature. A second wash was performed with wash solution 2 (0.1% NP40 and 2× SSC) for 3 min at room temperature and the coverglass was allowed to dry in the dark. Cells were stained with DAPI (2 µg ml^−1^) for 10 min at room temperature, washed with D-PBS and water and mounted with ProLong Glass Antifade Mountant (Invitrogen). The samples were left in the dark overnight at room temperature to cure mountant before imaging.

Imaging was performed using the Leica TCS SP5X confocal microscope with a ×63 oil immersion objective, with a *z*-stack collected for each channel (step size 0.2 μm). Images were acquired with LAS X software (v.1.9.0, Leica). ImageJ software (v.2.0.0-rc-65/1.52q, build: 961c5f1b7f) was used to process images. Nuclear lamina and speckles were identified by lamin B1 and SON immunostaining, respectively. 3D reconstructions of cells were conducted in Imaris x64 (v.9.2.1) software (Bitplane). DNA-FISH dots for probe signals were created at the location of the highest fluorescence intensity using the Spots tool. The spot diameter ranged from 350 nm to 400 nm. Nuclear lamina/speckle surfaces were automatically detected from lamin B1/SON immunostaining using the Surfaces tool. The distance from the centre of the FISH spot to the closest lamina/speckle surface was quantified using the Measurement Points tool. For each experimental condition, 40–50 nuclei were analysed. In the case that the generated DNA-FISH spot was embedded in the laminar/speckle surface, the distance to the lamina/speckle was quantified as zero.

### Plasmid construction

LacI plasmids were constructed using the In-Fusion Snap Assembly method (Takara). GFP-LacI-NLS transgene (derived from Addgene plasmid 183920, from K. Rippe) was inserted into the pCMV-N-Flag vector to generate pCMV-Flag-GFP-LacI. Human lamin A CDS (derived from Addgene plasmid 129275, from A. Ting) was inserted C terminally to LacI to generate pCMV-Flag-GFP-LacI-LMNA. pEF-1α-pLacO-RFP was purchased from Addgene (179507, from Y. Hiraoka).

### HEK293T cell culture, plasmid biotinylation and transfection

HEK293T cells (ATCC) were cultured in DMEM supplemented with 10% FBS and 1% antibiotic-antimycotic (Sigma-Aldrich). pEF-1α-pLacO-RFP plasmid was biotinylated and repurified using the Label-IT Tracker Intracellular Nucleic Acid Localization Kit, Biotin (Mirus) according to the manufacturer’s instructions. Biotinylated and unmodified plasmids were transfected into HEK293T cells using Lipofectamine 3000 Transfection Reagent (Invitrogen) and collected 3 days after transfection. For drug treatment experiments, cells were treated with 5 µM EPZ-6438 or an equivalent volume of DMSO for 4 days before transfection. The drug was replenished every other day and treatment was continued after transfection until collection.

### Immunofluorescence and plasmid visualization

HEK293T cells transfected with GFP-LacI/GFP-LacI-LMNA and biotinylated pEF-1α-pLacO-RFP were fixed with 4% paraformaldehyde for 20 min at room temperature. Cells were washed in PBS and free aldehydes were quenched with 125 mM glycine in TBS. Cells were then permeabilized and blocked (5% normal donkey serum, 5% normal goat serum (Jackson Immunoresearch), 0.25% Triton X-100 in PBS) for 1 h at room temperature. Cells were incubated with rabbit anti-lamin B1 (Abcam, ab16048, 1:250) and chicken anti-GFP (Abcam, ab13970, 1:500) primary antibodies, diluted in antibody buffer (5% normal donkey serum, 5% normal goat serum, 0.1% Triton X-100 in PBS) overnight at 4 °C. The next day, cells were washed four times for 5 min in PBST followed by incubation in antibody buffer with Streptavidin Alexa Fluor 647, donkey anti-rabbit Alexa Fluor 555 and goat anti-chicken Alexa Fluor 488 (Invitrogen, all 1:500) for 2 h at room temperature in the dark. Cells were then washed five times in PBST, incubated with 1 µg ml^−1^ DAPI in PBS for 5 min, and mounted with ProLong Glass Antifade Mountant. The slides were imaged on the Leica Stellaris confocal microscope with a ×63 objective. The plasmid distance to the lamina was analysed in Imaris as described for DNA-FISH.

### Western blotting

Cultured cells were washed with ice-cold PBS and collected on ice in RIPA buffer (50 mM Tris-HCl pH 7.5, 150 mM NaCl, 1 mM EDTA, 0.1% SDS, 0.5% sodium deoxycholate) supplemented with 1× Halt protease inhibitor cocktail. The lysates were sonicated on ice and centrifuged at 3,000*g* for 10 min at 4 °C. Then, β-mercaptoethanol was added to supernatants to a final concentration of 5%, and the samples were mixed with LDS sample buffer (Thermo Fisher Scientific) and boiled for 5 min. Next, 20 μg of protein was resolved by SDS–PAGE. Protein was transferred onto PVDF membranes, which were blocked in 5% BSA in TBS with 0.1% Tween-20 (TBST) followed by overnight incubation in primary antibodies (diluted 1:1,000 in blocking buffer) at 4 °C. The membranes were washed in TBST and incubated in HRP-tagged anti-rabbit secondary antibody (Cell Signaling, diluted 1:5,000 in blocking buffer) for 1 h at room temperature. Proteins were visualized using SuperSignal West Pico Plus Chemiluminescent Substrate (Thermo Fisher Scientific) and imaged on the LICOR Odyssey XF system.

### RNA isolation and RT–qPCR

Cells were collected in TRIzol (Invitrogen) and RNA isolated using the Direct-zol RNA Miniprep kit (Zymo) according to the manufacturer’s instructions. cDNA was reverse transcribed from 0.5 µg RNA using the Transcriptor First Strand DNA Synthesis Kit (Roche) with oligo-dT primers according to the manufacturer’s instructions. qPCR with reverse transcription (RT-qPCR) was performed in technical triplicate on the LightCycler 480 Instrument (Roche) using the LightCycler 480 SYBR Green I Master Mix (Roche) according to the manufacturer’s instructions. Triplicate values were averaged and RNA expression was determined using the ΔΔ*C*_t_ method using *GAPDH* as the loading control.

### Chromatin immunoprecipitation

HEK293T cells in 10 cm dishes were fixed with 1% formaldehyde for 10 min at room temperature, quenched for 5 min with 125 mM glycine and washed twice in PBS. Cells were collected in lysis buffer 1 (50 mM HEPES pH 7.5, 140 mM NaCl, 1 mM EDTA, 10% glycerol, 0.5% NP-40, 0.25% Triton X-100 and 1× Halt protease inhibitor cocktail (Thermo Fisher Scientific, included in all buffers)) and freeze–thawed in liquid nitrogen. During thawing, protein G Dynabeads (Invitrogen) were washed and incubated with 1 µg normal rabbit IgG, rabbit anti-H3 or rabbit anti-H3K27me3 (Cell Signaling) in 0.5% BSA/PBS for 2–4 h at 4 °C. Thawed cells were Dounce homogenized 20 times with type B pestle to liberate nuclei, which were pelleted at 500*g* for 5 min. The nuclear pellet was resuspended in lysis buffer 2 (10 mM Tris-HCl pH 8, 200 mM NaCl, 1 mM EDTA, 0.5 mM EGTA), rotated at 4 °C for 10 min and centrifuged at 500*g* for 5 min. The washed pellet was resuspended in lysis buffer 3 (10 mM Tris-HCl, pH 8, 100 mM NaCl, 1 mM EDTA, 0.5 mM EGTA, 0.1% sodium deoxycholate) and sonicated in a Bioruptor ice bath sonicator on high power for 25 cycles (30 s on/30 s off). Before the final ten cycles, Triton X-100 was added to a final concentration of 1%. Sonicated lysates were cleared at 16,000*g* for 10 min at 4 °C. The supernatant was split equally among three tubes (10% split sample volume saved as input), mixed with appropriate bead–antibody complex and rotated overnight at 4 °C. The next day, beads were washed five times for 2 min at room temperature in wash buffer 1 (50 mM HEPES pH 7.5, 500 mM LiCl, 1 mM EDTA, 1% NP-40, 0.7% sodium deoxycholate) and once in wash buffer 2 (10 mM Tris-HCl pH 8, 1 mM EDTA, 50 mM NaCl). Chromatin was eluted from beads by shaking at 65 °C for 30 min in elution buffer (50 mM Tris-HCl pH 8, 10 mM EDTA, 1% SDS) then transferred to a fresh tube. NaCl (final concentration 540 mM) and proteinase K (0.2 mg ml^−1^) were added to ChIP and input samples followed by overnight incubation at 65 °C. The next day, the samples were treated with RNase A (0.1 mg ml^−1^) for 30 min at 37 °C. DNA was then purified by 1.8 AMPure XP Bead (Beckman) cleanup and eluted in 0.1× TE buffer. qPCR was performed as described above.

### Reporting summary

Further information on research design is available in the Nature Portfolio Reporting Summary linked to this article.

## Online content

Any methods, additional references, Nature Portfolio reporting summaries, source data, extended data, supplementary information, acknowledgements, peer review information; details of author contributions and competing interests; and statements of data and code availability are available at https://doi.org/10.1038/s41586-026-10832-w.

## Supplementary information

Supplementary Information Supplementary Figs. 1 and 2 and Supplementary Table 2.

Reporting Summary

Supplementary Table 1 Sample metadata.

Peer Review File

## Data Availability

Raw and processed sequencing data for human cell lines, along with processed data from human fetal brain samples, have been deposited at the GEO under accession numbers GSE274038 and GSE274039 and are publicly available. Raw sequencing data from human fetal brain tissue are available under controlled access through the NIH database of Genotypes and Phenotypes (dbGaP; phs000989.v7.p1) to protect donor privacy and comply with the terms of informed consent. Access requests should be submitted via the dbGaP authorized access portal (https://dbgap.ncbi.nlm.nih.gov) to the JAAMH data access committee (JAAMH-DAC@list.nih.gov) and require local IRB approval and execution of a data use certification limiting use to health/medical/biomedical research (HMB-IRB). Data access requests are typically processed within 2 weeks. No additional restrictions on downstream reuse or authorship apply beyond those specified in the data use certification. Gene expression data from human fetal cortex used in this study are available through the NeMO archive (https://assets.nemoarchive.org/dat-uioqy8b). fastGRO-seq datasets analysed in this study were obtained from GSE143844.

## Code availability

All of the software used in this study are listed in the Reporting Summary and Supplementary Table 2. The code used for data analysis is available at GitHub (https://github.com/eugene-gil/LADs-SPADs-HumanCortex).
