# Supplementary material for: Subnuclear genome compartmentalization controls bivalent chromatin activity
Source: Nature. 2026 Jul 22;657(8131):539–48. doi: 10.1038/s41586-026-10832-w (PMC13558055; doi:10.1038/s41586-026-10832-w)
Supplement: Supplementary file 1 [file 41586_2026_10832_MOESM1_ESM.pdf]

nature portfolio

https://doi.org/10.1038/s41586-026-10832-w

Supplementary information

Subnuclear genome compartmentalization controls bivalent chromatin activity

In the format provided by the authors and unedited

Subnuclear genome compartmentalization controls bivalent chromatin activity

Authors and Affiliations:
Sajad Hamid Ahanger[1,2 †], Evan R. Semenza[1,2,3 †], Chujing Zhang[1,2 †], Eugene Gil[1,2], Mitchel A. Cole[1,2,3,4], Serena Huei-An Lu[1,2,3], Li Wang[2,5], Arnold R. Kriegstein[2,5], Daniel A. Lim[1,2,6, *]

[1]Department of Neurological Surgery;
[2]Eli and Edythe Broad Center of Regeneration Medicine and Stem Cell Research;
[3]Biomedical Sciences Graduate Program;
[4]Medical Scientist Training Program;
[5]Department of Neurology;
[6]San Francisco Veterans Affairs Medical Center;
University of California, San Francisco, San Francisco, CA 94143, USA.

[†] Equal contribution

* Corresponding author

Lead contact: Daniel.Lim@ucsf.edu

Supplementary Information Guide

Supplementary Figure 1. Raw images of Western blots (page 3).

Supplementary Figure 2. Summary figure (page 4).

Supplementary Table 1. Sample metadata (provided as .xlsx).

Supplementary Table 2. Reagent and resource table (page 5).

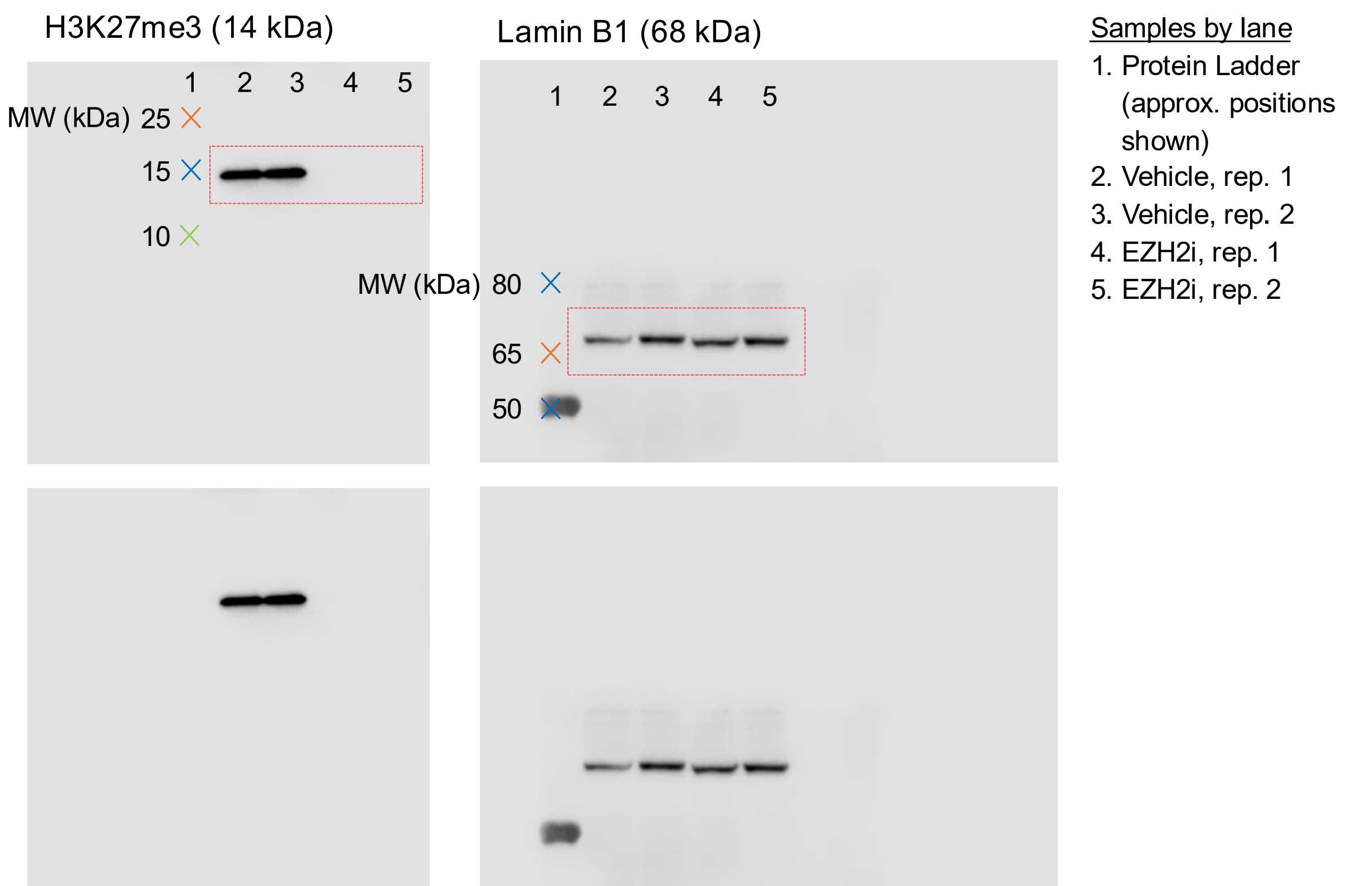

Supplementary Figure 1. Raw data for Western blots shown in Extended Data Fig. 9j. Unprocessed, uncropped blots are displayed with (top) and without (bottom) sample and molecular weight annotations. Red boxes denote portions of blots displayed in Extended Data Fig. 9j.

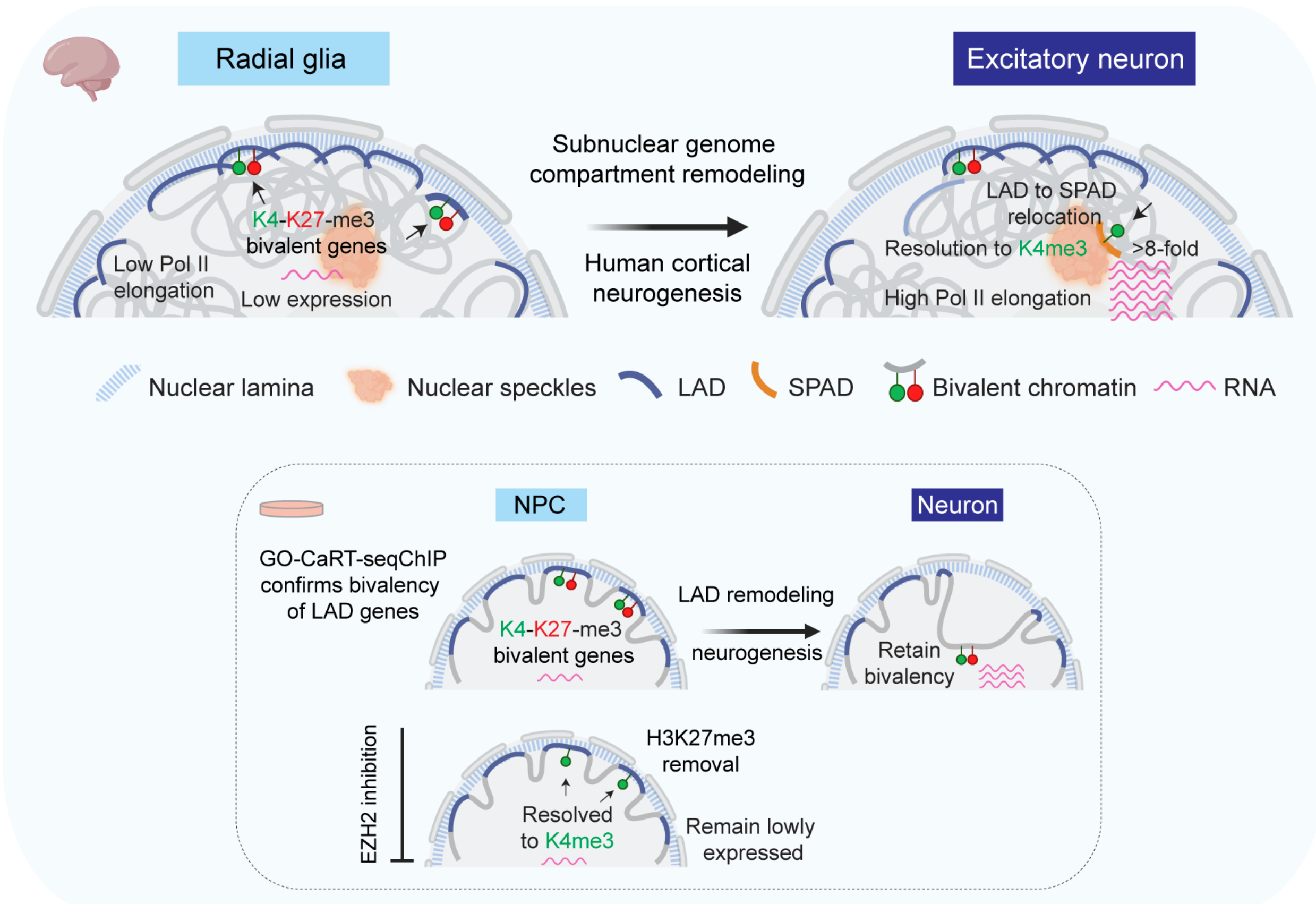

Supplementary Figure 2. Model of subnuclear genome compartmentalization dynamics regulating bivalent chromatin during human neurogenesis. Top: In neural progenitor cells (Radial glia) of the developing human brain, neurodevelopmental genes marked by H3K4me3-H3K27me3 bivalent chromatin are enriched at the nuclear lamina and are expressed at very low levels. During cortical neurogenesis (RG to EN), extensive remodeling of the spatial genome organization relocates hundreds of neuronal genes from the lamina to nuclear speckles. This lamina-to-speckle translocation is associated with enhanced resolution of bivalency to the H3K4me3-monovalent state and robust transcriptional upregulation. The repressive lamina environment is characterized by spatial segregation of RNA polymerase II (Pol II) elongation machinery from the nuclear periphery, whereas loss of lamina-association enables productive transcription in the nuclear interior. **Bottom:** Cell culture models of human neurogenesis confirm the bivalent state of genes embedded in the lamina and demonstrate that lamina proximity– and not the presence of H3K27me3– is the primary determinant of low transcriptional output of bivalent genes.

Supplementary Table 2: Key Reagents and Resources

Reagent or Resource	Source	Identifier
**Antibodies**		
Alexa Fluor® 488 Mouse anti-Human Pax-6	BD Biosciences	561664
EOMES Monoclonal Antibody (WD1928), PE-Cyanine7	Invitrogen	25-4877-42
Recombinant Alexa Fluor® 647 Anti-SATB2 antibody	Abcam	ab196536
AffiniPure Fab Fragment Donkey Anti-Rabbit IgG (H+L)	Jackson Immuno	711-007-003
AffiniPure Fab Fragment Donkey Anti-Mouse IgG (H+L)	Jackson Immuno	715-007-003
Anti-Lamin B1 antibody	Abcam	ab16048
Anti-SON antibody	Atlas antibodies	HPA031755
Tri-Methyl-Histone H3 (Lys27) rabbit mAb	Cell Signaling Technology	9733
Tri-Methyl-Histone H3 (Lys4) rabbit mAb	Cell Signaling Technology	9751
Histone H3K9me2 pAb	Active Motif	39239
Histone H3 mAb	Active Motif	39064
Histone H3 rabbit mAb	Cell Signaling Technology	4499
Anti-TUBB3 (Tuj1) antibody	BioLegend	801201
Anti-NeuN antibody	Abcam	ab177487
Sox2 antibody	Santa Cruz Biotechnology	sc-365823
PAX6 antibody	Biolegend	901301
Phospho-Rpb1 CTD (Ser2) rabbit mAb	Cell Signaling Technology	13499
Phospho-Rpb1 CTD (Ser5) rabbit mAb	Cell Signaling Technology	13523
Rpb1 CTD (4H8) mouse mAb	Cell Signaling Technology	2629
Lamin A/C mouse mAb	Cell Signaling Technology	4777
Cyclin T1 rabbit mAb	Cell Signaling Technology	81464
MLLT1/ENL rabbit mAb	Cell Signaling Technology	14893
Anti-CDK9 antibody	Abcam	ab239364
NELF-A pAb	ProteinTech	10456-1-AP
COBRA1 (NELF-B) rabbit mAb	Cell Signaling Technology	14894
TH1L (NELF-C/D) rabbit mAb	Cell Signaling Technology	12265
NELF-E pAb	ProteinTech	10705-1-AP
GFP antibody	Abcam	ab13970
Normal Rabbit IgG	Cell Signaling Technology	2729S

Guinea Pig anti-Rabbit IgG	Antibodies-online.com	ABIN101961
Donkey anti-Rabbit IgG (H+L) Antibody, Alexa Fluor™ Plus 488	Invitrogen	A32790
Donkey anti-Mouse IgG (H+L) Antibody, Alexa Fluor™ Plus 647	Invitrogen	A32787
Goat anti-Chicken IgY (H+L) Antibody, Alexa Fluor™ 488	Invitrogen	A11039
Donkey anti-Rabbit IgG (H+L) Highly Cross-Absorbed Antibody, Alexa Fluor™ 555	Invitrogen	A31572
Anti-rabbit IgG, HRP-linked antibody	Cell Signaling Technology	7074
**Peptides, recombinant proteins, chemicals**		
Mouse Lamin B1 peptide	Abcam	ab16375
Histone H3K27me3 Trimethyl Peptide	EpigenTek	R-1034-200
Histone H3K4me3 Trimethyl Peptide	EpigenTek	R-1022-200
Streptavidin, Alexa Fluor™ 647 conjugate	Invitrogen	S21374
pA/G-MNase	Macro Lab (UC Berkeley)	-
pA-Tn5	Macro Lab (UC Berkeley)	-
Proteinase K Solution (20 mg/mL)	Thermo Fisher	AM2548
DNase-free RNase A	Thermo Fisher	EN0531
RiboLock RNase Inhibitor	Thermo Fisher	EO0382
RNasin® Plus Ribonuclease Inhibitor	Promega	N2615
Glycogen, RNA grade	Thermo Fisher	R0551
Spermidine >99% (GC)	Sigma	S2626-1G
ProLong™ Glass Antifade Mountant	Thermo Fisher	P36980
Tazemetostat (EPZ-6438)	Selleckchem	S7128
UltraPure™ Phenol:Chloroform:Isoamyl Alcohol (25:24:1, v/v)	Thermo Fisher	15593031
Pierce™ 16% Formaldehyde (w/v), Methanol-free	Thermo Fisher	28906
HistoChoice® Tissue Fixative	Millipore Sigma	H2904
Halt™ Protease Inhibitor Cocktail (100X)	Thermo Fisher	78438
Digitonin solution- high purity	Millipore Sigma	CHR103
Trizol reagent	Thermo Fisher	15596018
Dynabeads® Protein A	Thermo Fisher	10002D
Dynabeads® Protein G	Thermo Fisher	10003D
BioMag®Plus Concanavalin A	Polysciences	86057-10
RNAClean XP	Beckman Coulter	A63987
Agencourt AMPure XP	Beckman Coulter	A63881
SPRIselect Reagent	Beckman Coulter	B23318

ReLeSR	StemCell Technologies	5872
StemPro Accutase	Thermo Fisher	A1110501
Matrigel® Growth Factor Reduced (GFR) Basement Membrane Matrix	Corning	356231
Laminin	Millipore Sigma	L2020-1MG
Fibronectin bovine plasma	Millipore Sigma	F4759-1MG
Poly-L-ornithine hydrobromide	Millipore Sigma	P3655-50MG
Poly-D-lysine hydrobromide	Millipore Sigma	P0899-50MG
StemFlex Medium	Thermo Fisher	A3349401
DMEM/F-12, GlutaMAX™ supplement	Thermo Fisher	10565042
Neurobasal-A Medium	Thermo Fisher	10888022
B27 minus Vitamin A	Invitrogen	12587-010
N2 supplement	Life Technologies	17502-048
DMEM	Corning	10-017-CV
FBS	VWR	97068-085
Antibiotic/antimycotic 100X	Thermo Fisher	15240062
L-Glutamine 200mM	Thermo Fisher	25030081
Y-27632 ROCK inhibitor	Selleckchem	S1049
Recombinant Human EGF	PeproTech	AF-100-15
Recombinant Human FGF-basic	PeproTech	100-18B
Insulin-Transferrin-Selenium (ITS-G)	Thermo Fisher	41400045
L-Ascorbic acid 2-phosphate sesquimagnesium salt	Santa Cruz Biotech	sc-228390
LDN-193189	Selleckchem	S2618
SB431542	Selleckchem	S1067
XAV939	Cayman Chemical	13596
N6,2′-O-Dibutyryladenosine 3′,5′-cyclic monophosphate sodium salt	Millipore Sigma	D0627-100MG
Recombinant Human/Murine/Rat BDNF	PeproTech	450-02
Recombinant Human GDNF	PeproTech	450-10
DAPT	MedChemExpress	HY-13027
**Critical commercial assays**		
KAPA HyperPrep Kit with KAPA Library Amplification Primer Mix (10X)	Kapa	KK8504
TruSeq DNA Single Indexes Set A (12 Indexes, 24 Samples	Illumina	20015960
TruSeq DNA Single Indexes Set B (12 Indexes, 24 Sample)	Illumina	20015961
NEBNext® rRNA Depletion Kit v2 (Human/Mouse/Rat)	New England Biolabs	E7400L

NEBNext® Ultra™ II Directional RNA Library Prep with Sample Purification Beads	New England Biolabs	E7765S
NEBNext® Multiplex Oligos for Illumina® (Index Primers Set 3)	New England Biolabs	E7710S
NEBNext® Multiplex Oligos for Illumina® (Index Primers Set 4)	New England Biolabs	E7730S
NEBNext® High-Fidelity 2X PCR Master Mix	New England Biolabs	M0541
Papain Dissociation System	Worthington	LK003150
AbC™ Total Antibody Compensation Bead Kit	Thermo Fisher	A10513
Qubit dsDNA HS Assay Kit	Thermo Fisher	Q32854
RNA ScreenTape	Agilent	5067-5576
RNA ScreenTape Sample Buffer	Agilent	5067-5577
D1000 ScreenTape	Agilent	5067-5582
D1000 Reagents	Agilent	5067-5583
High Sensitivity D1000 ScreenTape	Agilent	5067-5584
High Sensitivity D1000 Reagents	Agilent	5067-5585
Direct-Zol RNA Miniprep	Zymo research	R2052
Quick-RNA FFPE Miniprep	Zymo research	R1008
Phase Lock Gel Heavy	VWR	10847-802
8-well Chambered Coverglass w/ non-removable wells (1.5 Borosilicate Glass)	Thermo Fisher	155409PK
InFusion Snap Assembly Master Mix	Takara	638948
Label IT® Tracker™ Intracellular Nucleic Acid Localization Kit, biotin	Mirus Bio	MIR 7024
**Experimental models: Cell lines**		
HEK293T	ATCC	
WTC11-hiPSC	Conklin lab; Kreitzer et al., 2013	
**DNA-FISH probes (genomic coordinates, hg38)**		
NRG3 locus (chr10:81994239-82200685)	Empire Genomics	RP11-828D16
SYT1 locus (chr12:79120096-79278995)	Empire Genomics	RP11-76L7
ANKS1B locus (chr12:99144442-99346123)	Empire Genomics	RP11-315N20
SATB2 locus (chr2:199280574-199450448)	Empire Genomics	RP11-755G8
MEF2C locus (chr5:88738836-88895936)	Empire Genomics	RP11-1147F22

DNMT1 locus (chr19:10027189-10224063)	Empire Genomics	RP11-298C17
**Plasmids**		
pAG/MNase	Addgene	123461
3XFlag-pA-Tn5-Fl	Addgene	124601
GFP-LacI	Addgene	183920
V5-APEX2-LMNA	Addgene	129275
pLacO-pEF1a-RFP	Addgene	179507
pCMV-FLAG-GFP-LacI	This paper	
pCMV-FLAG-GFP-LacI-LmnA	This paper	
**Software**		
Trim Galore (v0.4.5)	Babraham Bioinformatics	
Picard Tools (v1.141)	Broad Institute	
Bowtie2 (v2.3.5.1)	Langmead & Salzberg (2012)	PMID:22388286
SAMtools (v1.12)	Danecek *et al.* (2021)	PMID:33590861
deepTools (v3.5.5)	Ramirez *et al.* (2016)	PMID:27079975
BEDTools (v2.31.0)	Quinlan and Hall (2010)	PMID:20110278
ggPlot2 (v4.0.2)	Wickham (2016)	ggplot2.tidyverse.org
Pomegranate (v1.1.2)	Schreiber (2018)	pomegranate.readthedocs.io
SEACR (v1.3)	Meers *et al.* (2019)	PMID:31300027
MACS2 (v2.1.2)	Zhang *et al.* (2008)	PMID:18798982
HOMER (v4.11)	Heinz *et al.* (2010)	PMID:20513432
STAR (v2.7.11b)	Dobin *et al.* (2013)	PMID:23104886
featureCounts (v2.24.0)	Yang *et al.* (2014)	PMID:24227677
DESeq2 (v1.36.0)	Love *et al.* (2014)	PMID:25516281
clusterProfiler (v4.4.4)	Yu *et al.* (2012)	PMID:22455463
RSEM (v1.3.3)	Li and Dewey (2011)	PMID:21816040
biomaRt	Durinck *et al.* (2009)	PMID:19617889
cutadapt (v4.4)	Martin (2011)	doi.org/10.14806/ej.17.1.200
LAS X (v1.9.0)	Leica	
Imaris x64 (v9.2.1)	Bitplane	
ImageJ (v2.0.0-rc65/1.52q)	NIH	
**qPCR Primers**	**Forward primer**	**Reverse Primer**
RFP	CCCGCCGACATCCCCGACTA	GGGTCACGGTCACCACGCC
GAPDH	CAATGACCCCTTCATTGACC	TTGATTTTGGAGGGATCTCG
ChIP - pLacO: EF1a prom	cacatgtggaattcctcgacta	actgcacttatatacggttctcc
ChIP - pLacO: RFP TSS	agtgggtggagactgaagtta	gaagcgcatgaactccttga
ChIP - pLacO: RFP TES	gactacaccatcgtggaacag	ctacaaatgtggtatggctgattatg
ChIP - pLacO: SV40 prom	tccgctcatgagacaataacc	accctaactgacacacattcc
