# Supplementary material for: Subnuclear genome compartmentalization controls bivalent chromatin activity
Source: Nature. 2026 Jul 22;657(8131):539–48. doi: 10.1038/s41586-026-10832-w (PMC13558055; doi:10.1038/s41586-026-10832-w)
Supplement: Supplementary file 2 [file 41586_2026_10832_MOESM2_ESM.pdf]

nature portfolio

Corresponding author(s): Daniel Lim

Last updated by author(s): Apr 24, 2026

Reporting Summary

Nature Portfolio wishes to improve the reproducibility of the work that we publish. This form provides structure for consistency and transparency in reporting. For further information on Nature Portfolio policies, see our Editorial Policies and the Editorial Policy Checklist.

Statistics

For all statistical analyses, confirm that the following items are present in the figure legend, table legend, main text, or Methods section.

n/a	Confirmed	
☐	☒	The exact sample size (*n*) for each experimental group/condition, given as a discrete number and unit of measurement
☐	☒	A statement on whether measurements were taken from distinct samples or whether the same sample was measured repeatedly
☐	☒	The statistical test(s) used AND whether they are one- or two-sided<br>*Only common tests should be described solely by name; describe more complex techniques in the Methods section.*
☒	☐	A description of all covariates tested
☐	☒	A description of any assumptions or corrections, such as tests of normality and adjustment for multiple comparisons
☐	☒	A full description of the statistical parameters including central tendency (e.g. means) or other basic estimates (e.g. regression coefficient) AND variation (e.g. standard deviation) or associated estimates of uncertainty (e.g. confidence intervals)
☐	☒	For null hypothesis testing, the test statistic (e.g. *F*, *t*, *r*) with confidence intervals, effect sizes, degrees of freedom and *P* value noted<br>*Give P values as exact values whenever suitable.*
☒	☐	For Bayesian analysis, information on the choice of priors and Markov chain Monte Carlo settings
☒	☐	For hierarchical and complex designs, identification of the appropriate level for tests and full reporting of outcomes
☐	☒	Estimates of effect sizes (e.g. Cohen's *d*, Pearson's *r*), indicating how they were calculated

Our web collection on statistics for biologists contains articles on many of the points above.

Software and code

Policy information about availability of computer code

Data collection	Raw sequencing data was generated using 150-bp paired-end sequencing on NovaSeq 6000 or NovaSeq X Plus systems (Illumina).<br>Images were collected using Leica LAS X v1.9.0, FANS data was collected in BD FACS Diva v9.0.
Data analysis	We used the following softwares in our study: Trim Galore v0.4.5, Picard Tools v1.141, bowtie2 v2.3.5.1, SAMtools v1.12, deepTools v3.5.5, BEDTools v2.31.0, ggPlot2 v4.0.2, pomegranate v1.1.2, SEACR v1.3, MACS2 v2.1.2, HOMER v4.11, STAR v2.7.11b, featureCounts v2.24.0, DESeq2 v1.36.0, clusterProfiler v4.4.4, RSEM v1.3.3, Ensembl BioMart release 115, cutadapt v4.4, Imaris x64 v9.2.1, ImageJ v2.0.0-rc65/1.52q<br><br>The code used for data analysis is available on GitHub (https://github.com/eugene-gil/LADs-SPADs-HumanCortex).

For manuscripts utilizing custom algorithms or software that are central to the research but not yet described in published literature, software must be made available to editors and reviewers. We strongly encourage code deposition in a community repository (e.g. GitHub). See the Nature Portfolio guidelines for submitting code & software for further information.

Data

Policy information about availability of data

All manuscripts must include a data availability statement. This statement should provide the following information, where applicable:

- Accession codes, unique identifiers, or web links for publicly available datasets
- A description of any restrictions on data availability
- For clinical datasets or third party data, please ensure that the statement adheres to our policy

Raw and processed sequencing data for human cell lines, along with processed data from human fetal brain samples, have been deposited in GEO under accession numbers GSE274038 and GSE274039 and are publicly available. Raw sequencing data from human fetal brain tissue have been deposited in dbGaP under accession number phs000989.v7.p1(https://www.ncbi.nlm.nih.gov/projects/gap/cgi-bin/study.cgi?study_id=phs000989.v7.p1) and are available through controlled access. Gene expression data from human fetal cortex used in this study are available through the NeMO archive (https://assets.nemoarchive.org/dat-uioqy8b). fastGRO-seq datasets analyzed in this study were obtained from GSE143844.

Research involving human participants, their data, or biological material

Policy information about studies with human participants or human data. See also policy information about sex, gender (identity/presentation), and sexual orientation and race, ethnicity and racism.

Reporting on sex and gender	No gender or sex-based analyses was performed in this study. Samples was obtained from de-identified donors with previous patient consent in strict observance of the legal and institutional ethical regulations.
Reporting on race, ethnicity, or other socially relevant groupings	No race, ethnicity- or other socially relevant groupings-based analyses was performed in this study. Samples used in this study were obtained from de-identified donors with previous patient consent.
Population characteristics	Because of the sensitivity of the samples, no population characteristics are known or recorded.
Recruitment	No recruitment criteria other than consent were required. Primary human brain tissue (Gestational week 20) was obtained from de-identified donors with previous patient consent in strict observance of the legal and institutional ethical regulations.
Ethics oversight	Protocols were approved by the Human Gamete, Embryo, and Stem Cell Research Committee (institutional review board) at the University of California, San Francisco. All experiments were performed in accordance with protocol guidelines. Informed consent was obtained before sample collection and use for this study.

Note that full information on the approval of the study protocol must also be provided in the manuscript.

Field-specific reporting

Please select the one below that is the best fit for your research. If you are not sure, read the appropriate sections before making your selection.

☒ Life sciences ☐ Behavioural & social sciences ☐ Ecological, evolutionary & environmental sciences

For a reference copy of the document with all sections, see nature.com/documents/nr-reporting-summary-flat.pdf

Life sciences study design

All studies must disclose on these points even when the disclosure is negative.

Sample size	Human data was generated for at least two biological replicates. The cortical tissue was obtained from brains of two de-identified subjects at gestational (GW) weeks 17 and 20. The biological replicates showed high concordance. No statistical methods were used to pre-determine sample sizes but our sample sizes are similar to those reported in previous publications:<br>https://www.nature.com/articles/nature06947<br>https://www.nature.com/articles/s41593-021-00879-5<br>https://www.sciencedirect.com/science/article/pii/S1097276510003217<br>https://rupress.org/jcb/article/217/11/4025/120670/Mapping-3D-genome-organization-relative-to-nuclear
Data exclusions	“No data were excluded from the analyses”.
Replication	GO-CaRT and CUT&RUN experiments on Radial glia (RG), Intermediate progenitor cells (IPC) and Excitatory neurons (EN) were performed on nuclei purified by fluorescence-activated nuclei sorting (FANS) from fresh/or frozen cortical brain tissue at gestational weeks 17 and 20. ~25,000 sorted nuclei were used per antibody for GO-CaRT and CUT&RUN. These experiments were carried out on tissues from two different subjects. All attempts at replication were successful.<br>RNA-seq data was generated from GW20 RG, IPC and EN and data from GW17 published previously (https://www.nature.com/articles/s41586-020-2825-4) was also used in the analyses.<br>Experiments in iPSC-derived NPCs were carried out two times with high data reproducibility.<br>Immunofluorescence and DNA FISH experiments in human cortical cells were carried out in biological duplicate (cells from two different subjects). 30-50 cells were quantified across subjects per condition.

	Plasmid targeting experiments in HEK 293T cells were carried out in biological triplicate (three separate transfections and experiments using cells of different passage number on different days). All replicates performed were included in the final data.
Randomization	Randomization is not relevant to our study because we do not apply any differential treatment, intervention or perturbation to our samples. We compare 3D spatial genome architecture between the neurogenic lineage of RG, IPC and EN. In our experiments, human brain tissue was obtained from de-identified donors with similar developmental age (GW17 and GW20).
Blinding	For all experiments involving manual acquisition and quantification of data (DNA-FISH, immunocytochemistry), imaging and quantification was performed by two scientists. One of them was blinded to experimental groups during image acquisition or quantification.

Reporting for specific materials, systems and methods

We require information from authors about some types of materials, experimental systems and methods used in many studies. Here, indicate whether each material, system or method listed is relevant to your study. If you are not sure if a list item applies to your research, read the appropriate section before selecting a response.

Materials & experimental systems

n/a	Involved in the study
☐	☒ Antibodies
☐	☒ Eukaryotic cell lines
☒	☐ Palaeontology and archaeology
☒	☐ Animals and other organisms
☒	☐ Clinical data
☒	☐ Dual use research of concern
☒	☐ Plants

Methods

n/a	Involved in the study
☒	☐ ChIP-seq
☐	☒ Flow cytometry
☒	☐ MRI-based neuroimaging

Antibodies

Antibodies used

PAX6-Alexa Flour-488, Mouse (BD #561664, clone O18-1330) was used at 1:20 dilution for FANS and 1:100 for immunocytochemistry.

EOMES-PE-Cy7, Mouse (Invitrogen #25-4877-42, clone WD1928) was used at 1:20 for FANS.

SATB2-Alexa Flour-647, Rabbit (Abcam #ab196536, clone EPNCIR130A) was used at 1:100 for FANS.

LaminB1 antibody, Rabbit polyclonal (Abcam# ab16048) was used at 1:100 for GO-CaRT and CUT&Tag, 1:1000 for Western blot, and 1:500 for combined immunocytochemistry/DNA-FISH.

SON antibody, Rabbit polyclonal (Atlas antibodies #HPA031755) was used at 1:100 dilution for GO-CaRT, 1:500 for combined Immunocytochemistry/DNA-FISH.

AffiniPure Fab Fragment Donkey anti-Rabbit and anti-Mouse (Jackson #711-007-003 and 715-007-003) were used at 1:30 for Fab blocking after FANS

H3K27me3 antibody, Rabbit monoclonal (Cell Signaling technologies #9733, clone C36B11) was used at 1:50 for CUT&RUN and CUT&Tag, 1ug/reaction for ChIP, and 1:1000 for Western blot.

H3K4me3 antibody, Rabbit monoclonal (Cell Signaling #9751, clone C42D8) was used at 1:75 for CUT&RUN and CUT&Tag.

Normal Rabbit IgG (Cell signaling technologies #2729) was used at 1:100 for GO-CaRT, CUT&Tag and CUT&RUN and 1ug/reaction for ChIP.

Guinea Pig anti-Rabbit IgG (Antibodies-online #ABIN101961) was used at 1:100 for CUT&Tag.

Histone H3K9me2 pAb (Active Motif #39239) was used at 1:100 for CUT&RUN.

Histone H3 rabbit mAb (Cell Signaling #4499, clone D1H2) was used at 1ug/reaction for ChIP.

Anti-TUBB3 (Tuj1) antibody (BioLegend #801201, clone TUJ1), anti-NeuN antibody (Abcam #ab177487, clone EPR12763), Sox2 antibody (Santa Cruz Biotechnology #sc-365823, clone E-4), Pax6 antibody (BioLegend #901301), Histone H3 mAb (Active Motif #39064, clone MABI 0301), Lamin A/C mouse mAb (Cell Signaling #4777, clone 4C11), Cyclin T1 rabbit mAb (Cell Signaling #81464, clone D1B6G), MLLT1/ENL rabbit mAb (Cell Signaling #14893, clone D9M4B), anti-Cdk9 antibody (Abcam #ab239364, clone EPR22956-37), COBRA1 (NELF-B) rabbit mAb (Cell SIgnaling #14894, clone D6K9A), and TH1L (NELF-C/D) rabbit mAb (Cell Signaling #12265, clone D5G6W) were used at 1:100 for immunocytochemistry.

Chicken anti-GFP antibody (Abcam #ab13970) was used at 1:500 for immunocytochemistry.

Donkey anti-Rabbit IgG Alexa Fluor Plus 488, Donkey anti-Mouse IgG Alexa Fluor Plus 647, Donkey anti-Rabbit IgG Alexa Fluor 555, and Goat anti-Chicken IgY Alexa Fluor 488 (Invitrogen #A32790, A32787, A31572, A11039) were used at 1:500 for immunocytochemistry.

Anti-rabbit IgG, HRP-linked antibody (Cell Signaling #7074) was used at 1:5000 for Western blot.

Phospho-Rpb1 CTD (Ser2) rabbit mAb (Cell Signaling #13499, clone E1Z3G), Phospho-Rpb1 CTD (Ser5) rabbit mAb (Cell Signaling #13523, clone D9N5I), and Rpb1 CTD mouse mAb (Cell Signaling #2629, clone 4H8) were used at 1:20 for CUT&RUN.

NELF-A pAb (ProteinTech #10456-1-AP) and NELF-E pAb (ProteinTech #10705-1-AP) were used at 1:50 for CUT&RUN and 1:100 for immunocytochemistry.

Validation

All antibodies have previously been validated for immunodetection of their corresponding proteins (see publications below).

PAX6-Alexa Flour-488 (BD Biosci); EOMES-PE-Cy7 (Invitrogen); and SATB2-Alexa Flour-647 (Abcam): Song M et al., Cell-type-specific 3D epigenomes in the developing human cortex. Nature 2020, doi:10.1038/s41586-020-2825-4

anti LaminB1 antibody (Abcam): Poleshko A et al., Genome-Nuclear Lamina Interactions Regulate Cardiac Stem Cell Lineage Restriction. Cell 2017, doi:10.1016/j.cell.2017.09.018; Ahanger SH et al., Distinct nuclear compartment-associated genome architecture in the developing mammalian brain. Nat Neurosci 2021, doi:10.1038/s41593-021-00879-5

anti-SON antibody (Atlas):: Chen Y et al., Mapping 3D genome organization relative to nuclear compartments using TSA-Seq as a cytological ruler. J Cell Biol 2018, doi:10.1083/jcb.201807108; Ahanger SH et al., Distinct nuclear compartment-associated genome architecture in the developing mammalian brain. Nat Neurosci 2021, doi:10.1038/s41593-021-00879-5

anti-H3K27me3 antibody and anti-H3K4me3 antibody (Cell Signaling): Skene PJ & Henikoff S, An efficient targeted nuclease strategy for high-resolution mapping of DNA binding sites. Elife 2017, doi:10.7554/eLife.21856

Normal Rabbit IgG antibody (Cell Signaling) and Histone H3K9me2 pAb (Active Motif): Ahanger SH et al., Distinct nuclear compartment-associated genome architecture in the developing mammalian brain. Nat Neurosci 2021, doi:10.1038/s41593-021-00879-5

Histone H3 mAb (Active Motif): Mohan N et al., Alternative splicing of BAZ1A in colorectal cancer disrupts the DNA damage response and increases chemosensitization. Cell Death Dis 2024, doi:10.1038/s41419-024-06954-6

Histone H3 rabbit mAb (Cell Signaling): Bandopadhayay P et al., Neuronal differentiation and cell-cycle programs mediate response to BET-bromodomain inhibition in MYC-driven medulloblastoma. Nat Commun 2019, doi:10.1038/s41467-019-10307-9

anti-TUBB3/Tuj1 (BioLegend): Jongbloets BC et al., Stage-specific functions of Semaphorin7A during adult hippocampal neurogenesis rely on distinct receptors. Nat Commun 2017, doi:10.1038/ncomms14666

anti-NeuN (Abcam): Gobrecht P et al., Promotion of Functional Nerve Regeneration by Inhibition of Microtubule Detyrosination. J Neurosci 2016, doi:10.1523/JNEUROSCI.4486-15.2016

Sox2 antibody (Santa Cruz): Xiao Z et al., 3D reconstruction of a gastrulating human embryo. Cell 2024, doi:10.1016/j.cell.2024.03.041

Pax6 antibody (BioLegend): Ziffra RS et al., Single-cell epigenomics reveals mechanisms of human cortical development. Nature 2021, doi:10.1038/s41586-021-03209-8

GFP antibody (Abcam): Feeney L et al., RPA-Mediated Recruitment of the E3 Ligase RFWD3 Is Vital for Interstrand Crosslink Repair and Human Health. Mol Cell 2017, doi:10.1016/j.molcel.2017.04.021

Phospho-Rpb1 CTD (Ser2) and Phospho-Rpb1 CTD (Ser5) antibodies (Cell Signaling): Nakatani K et al., Inhibition of OCT4 binding at the MYCN locus induces neuroblastoma cell death accompanied by downregulation of transcripts with high-open reading frame dominance. Front Oncol 2024, doi:10.3389/fonc.2024.1237378

Rpb1 CTD antibody (Cell Signaling): Jalan M et al., RAD52 resolves transcription-replication conflicts to mitigate R-loop induced genome instability. Nat Commun 2024, doi:10.1038/s41467-024-51784-x

Lamin A/C antibody (Cell Signaling): Xia J et al., Chromatin heterogeneity modulates nuclear condensate dynamics and phase behavior. Nat Commun 2025, doi:10.1038/s41467-025-60771-9

Cyclin T1 antibody (Cell Signaling) and anti-CDK9 antibody (Abcam): Lysakovskaia K et al., Promoter-proximal RNA polymerase II termination regulates transcription during human cell type transition. Nat Struct Mol Biol 2025, doi:10.1038/s41594-025-01486-9

MLLT1/ENL antibody (Cell Signaling): Bishop TR et al., Acetyl-CoA biosynthesis drives resistance to histone acetyltransferase inhibition. Nat Chem Biol 2023, doi:10.1038/s41589-023-01320-7

COBRA1 (NELF-B) (Cell Signaling), TH1L (NELF-C/D) (Cell Signaling), NELF-A (ProteinTech) and NELF-E (ProteinTech) antibodies: Wu B et al., RNA polymerase II pausing factor NELF in CD8+ T cells promotes antitumor immunity. Nat Commun 2022, doi:10.1038/s41467-022-29869-2

Eukaryotic cell lines

Policy information about cell lines and Sex and Gender in Research

Cell line source(s)	HEK293T cells were obtained from ATCC.<br>WTC11 hiPSC line was obtained from WiCell Stem Cell Bank (Kreitzer et al., 2013).
Authentication	Cell lines were authenticated at the source.
Mycoplasma contamination	Cell lines were not tested for mycoplasma contamination
Commonly misidentified lines<br>(See ICLAC register)	No commonly misidentified cell lines were used in the study.

Plants

Seed stocks	*Report on the source of all seed stocks or other plant material used. If applicable, state the seed stock centre and catalogue number. If plant specimens were collected from the field, describe the collection location, date and sampling procedures.*
Novel plant genotypes	*Describe the methods by which all novel plant genotypes were produced. This includes those generated by transgenic approaches, gene editing, chemical/radiation-based mutagenesis and hybridization. For transgenic lines, describe the transformation method, the number of independent lines analyzed and the generation upon which experiments were performed. For gene-edited lines, describe the editor used, the endogenous sequence targeted for editing, the targeting guide RNA sequence (if applicable) and how the editor was applied.*
Authentication	*Describe any authentication procedures for each seed stock used or novel genotype generated. Describe any experiments used to assess the effect of a mutation and, where applicable, how potential secondary effects (e.g. second site T-DNA insertions, mosiacism, off-target gene editing) were examined.*

Flow Cytometry

Plots

Confirm that:

☒ The axis labels state the marker and fluorochrome used (e.g. CD4-FITC).

☒ The axis scales are clearly visible. Include numbers along axes only for bottom left plot of group (a 'group' is an analysis of identical markers).

☒ All plots are contour plots with outliers or pseudocolor plots.

☒ A numerical value for number of cells or percentage (with statistics) is provided.

Methodology

Sample preparation	Nuclei were isolated from fesh or frozen cortical brain tissue (GW17 and GW20) as described in the methods section. Nuclei were lightly fixed in 0.1% formaldehyde for 2 min, filtered through a 40 micron filter and stained with antibodies to PAX6, EOMES and SATB2. Nuclei were sorted for PAX6-positve (Radial glia), PAX6/EOMES-positive (Intermediate progenitor cells) and SATB2-positive (Excitatory neurons). Sorted nuclei were directly used for downstream GO-CaRT, CUT&RUN and RNA-seq analyses.
Instrument	Samples were sorted on a FACS Aria III cytometer (Becton, Dickinson Biosciences) with 70-micron size nozzle.
Software	Data collection was perfomed using FACsDiva, and representative images were created using FlowJo.
Cell population abundance	Radial glia represented ~6% singlets; Intermediate progenitor cells represented ~2% singlets and Excitatory neurons represented ~50% singlets.
Gating strategy	Nuclei were separated from debris by forward scatter area (FSC-A) versus side scatter area (SSC-A). Single cells (singlets) were then selected using two gates based on area versus height of the forward scatter (FSC-A vs. FSC-H) and side scatter (SSC-A vs. SSC-H). RG populations were isolated by gating on PAX6-Alexa Fluor 488 staining and IPC by gating on PAX6-Alexa Fluor 488 and EOMES-PE-Cy7 staining. EN were isolated from PAX6/EOMES negative population (P3) by gating on SATB2-Alexa Fluor 647 staining.

☒ Tick this box to confirm that a figure exemplifying the gating strategy is provided in the Supplementary Information.
