# Supplementary material for: Subnuclear genome compartmentalization controls bivalent chromatin activity
Source: Nature. 2026 Jul 22;657(8131):539–48. doi: 10.1038/s41586-026-10832-w (PMC13558055; doi:10.1038/s41586-026-10832-w)
Supplement: Supplementary file 3 [file 41586_2026_10832_MOESM4_ESM.pdf]

nature portfolio

Peer Review File

Subnuclear genome compartmentalization controls bivalent chromatin activity

Corresponding Author: Dr Daniel Lim

Any redactions in this file are there to maintain patient confidentiality, the confidentiality of unpublished data, or to remove third-party material.

Parts of this Peer Review File have been redacted as indicated to remove third-party material.

Attachments originally included by the reviewers as part of their assessment can be found at the end of this file.

Version 0:

Reviewer comments:

Referee #1

(Remarks to the Author)
In this manuscript, Ahanger and colleagues characterized the remodeling of subnuclear genome compartmentalization during neurogenesis by examining the genomic regions marked by LaminB1 and SON in RG, IPC, and EN using the developing human cortex. They further demonstrated that during neurogenesis, bivalent genes—co-marked by H3K4me3 and H3K27me3—relocate to speckles, resulting in increased gene expression, particularly for neurodevelopmental genes. Additionally, they concluded the key factor in maintaining the lowly expressed, poised state of bivalent genes is not H3K27me3. This is nice study, well-written, and the functional validation is nice convincing. There are a number of issues need to be addressed.

Major comments:
1. The authors concluded that 'the LaminB1 profiles from RG, IPC, and EN populations were highly similar between GW17 and GW20 (Fig. 1a, Extended Data Fig. 3a), indicating that these developmental time points are reasonable biological replicates for studies of LAD architecture'. Throughout the study, GW17 and GW20 were treated as replicates. However, there are notable differences in the correlation matrix of LaminB1 (Extended Data Fig. 3a), which suggests they may not be suitable as replicates. For instance, the correlation between IPC-GW17 and IPC-GW20 is 0.69, which represents the 'same group', but the correlation between RG-GW17 and IPC-GW17 (different groups) is actually higher at 0.72-. As a reference, when interpret the results from SON, the authors considered a Jaccard index of 0.59 as similar (Extended Data Fig. 4b), further raising questions about the validity of using these time points as replicates.
2. As indicated, the LaminB1 and SON regions were identified at scales ranging from ~Kb to Mb, while the histone-marked regions were identified at much smaller scales, from hundreds to thousands of base pairs. This difference in resolution makes it tricky to accurately identify overlapping regions, especially when calling the differential regions (gain, loss, same). The authors should make this clear and use the same standard throughout the study.
3. As we know, large domain which usually contain more than one gene, how the author chooses the associated genes for gene expression level and GO term analysis? And what is the rational for that?
4. The authors make a strong conclusion the dominant role of spatial genome positioning in regulating bivalent gene expression, however, the data presented does not fully support this claim. For example, in figures 4a and 6c, genes within H3K4me3 group consistently exhibit higher expression levels than those in the bivalent and H3K27me3 groups, regardless of whether those genes are located within LADs, non-LADs/non-SPADs, or SPADs. In an extreme case, H3K4me3 only genes within LADs express at a higher level than bivalent genes within SPADs. Does this suggest chromatin state plays a more significant role, while genome positioning serve as a “tuning” mechanism in transcriptional regulation?
5. Probably the strongest evidence presented in this manuscript to support the claim that spatial positioning plays a more important role than chromatin state in determining bivalent gene transcription comes from the EZH2 inhibition experiments. The results show that H3K27me3 depletion will not disrupt global LAD architecture and low-expression state of genes remained in LADs. However, it is a possible that other repressive chromatin modifications, like H3K9me2/3, are still present in these bivalent genes and are unaffected by H3K27me3 depletion. In this scenario, gene repression could still be attributed to chromatin state rather than solely to 3D genome positioning. Therefore, the authors have not fully disentangled

the interaction between spatial positioning and chromatin states and their respective contributions to gene transcription.
6. Since H3K9me2/3 are highly enriched in the LADs and play critical roles in LADs architecture and gene repression, the author could perform H3K9me2/3 CUT&RUN in RG, IPC and EN. It would be insightful to see how H3K9me2/3 changes during neurogenesis and how those changes relate to LAD reorganization and gene transcriptional changes. Additionally, the author might consider including DNA methylome profiling to the dataset, as DNA methylation is another crucial repressive modification in LADs and interacts extensively with H3K27me3.
7. It is not very clear how the LADs and SPADs change during neurogenesis. For example, how many LADs exist in all three cells? how many LADs only exist in RG? how many LADs only exist in EN? how many LADs lost in IPC and then regained in EN. They author could use a figure to summarize these changes.
8. The authors have not clearly shown the percentage of bivalent genes (as well as H3K4me3-only and H3K27me3-only genes) that are located within LADs, non-LADs/non-SPADs, and SPADs in the three cell types. This data would help readers better perceive to what extent bivalent genes are regulated by LADs.
9. One observation from the example cases is that the bivalent regions marked by H3K4me3 and H3K27me3 were depleted in LaminB1/SON domains, as shown in Fig. 4d, Fig. 5d, and Fig. 7d. Is this depletion a common feature throughout the genome? If so, do the authors have any thoughts on this pattern?
10. The authors used co-staining with DNA-FISH and LaminB1/SON to validate the movement of genes from the lamina to speckles. However, this measurement should be conducted in 3D rather than 2D (Fig. 3) for more accurate results.
11. In Fig 4C, the authors focused their analysis on bivalent regions at the RG state, but what about regions that become bivalent at the EN state? Exploring these regions could provide additional insights. In addition, it would be useful to examine the changes in expression levels for genes within the same Lamin/SON domain but with different H3K4me3/H3K27me3 features.
Minor points:
1. There is a typo in Fig. 4a and Extended Data Fig. 3c, which labeled “log1pTPM”, maybe should be “log10 TPM”.
2. The authors separately displayed the LADs and SPADs regions in figure 1 and figure 2 and noted that they are “largely mutually exclusive”. However, it is challenging for readers to compare those figures visually. The authors could consider combining LADs and SPADs in a single figure, and labeling them with different colors, and using with another color for overlapping regions. Meanwhile, they should provide the percentage of overlap between LADs and SPADs in the three cells.
3. The authors demonstrated that LAD-gain and LAD-loss are associated with the decrease and increase of gene expression during neurogenesis (RG to EN), respectively. However, the LAD-same regions also displayed significant gene expression changes between RG vs IPC and RG vs EN (Fig. 1D). The authors need to comment on this point.
4. When identifying differential LAD regions, the focus was only on RG and EN. What about IPC? Does it represent an intermediate state between these two?
5. The authors need to provide characterization of the cell identity of the in vitro cultures which they used for profiling and validation studies.

Referee #2

(Remarks to the Author)
Ahanger et al performed genomic mapping of LADs and SPADs in midgestational human cortex in radial glia, intermediate progenitor cells and excitatory neurons. They find extensive reorganizations of genomic regions in both spatial compartments that include genes with neurodevelopmental function. Spatial repositioning away from the nuclear lamina and subsequent association with SPADs or non-LAD regions is associated with an increase in gene expression. The authors also identify genes in LADs that are in a bivalent state, for which rendering to monovalent H3K4me3 is associated with gene activation.

I find the observations interesting as not much work is performed in in vivo settings, though I also find that minimal new biological insight is obtained on the mechanistic role for LADs and SPADs in gene regulation. The manuscript is descriptive and data is at times difficult to interpret due to lack of analytical descriptions or in-debt qualitative and quantitative analyses. The concept that is proposed in the abstract is interesting, yet at present, I find it an oversimplification and insufficiently supported by the data. I find this manuscript more suited for a specialized genomics or neuronal journal and not for the broad readership of Nature.

Comments in chronological order

-Line 167 reference to Fig. 1a should be Fig. 1b. This also makes that there is no reference to Fig. 1a.

-No LADs are found on five entire chromosomes. This is very surprising, unusual and unprecedented. This is a technical concern in relation to the sensitivity of the method. Perhaps due to the low-input cell number? This needs to be addressed i.e. by performing LAMINB1 GO-CaRT with dilution series of input material in comparable tissue types or iPCS. Or is this related to some form of computational thresholding that is applied? This I can’t assess because of insufficient descriptions on this part (see comment below).

-Jaccard index (wrongly named Jaccard correlation) between GW17 and GW20 IPC is lower than between IPC and RG GW17 and GW20. This makes me question whether the origin of the cell-type specific changes are technical or biological.

-What is the reason behind choosing GW17 and GW20 as replicates? I assume this is because of tissue availability, but

there is no explanation provided and these samples do not represent true biological replicates. This decision complicates data interpretation.

-Quantitative comparisons are performed between RG and EN stages for Fig ED3e-f. However, there is no mentioning of how this comparison is performed. I assume this is based on the combined data of the GW17 and GW20 sample of each of the three cell types? Also, how is the comparison performed? Is this based on domain calling or some binary cut-off? Smoothing or not? How is the data normalized and is the data quality between the three cell types compatible? All these factors are hugely important for data interpretation and evaluation and much more in debt descriptions (both in main and methods) and analyses are needed to substantiate and evaluate these findings.

-Fig. ED1d it is unclear if i.e. LAD loss in EN represents the change compared to IPC -> RG or both. It would be informative to include the number of genes involves per category. Also, the effect size is very small between most classes. With genomics data the tiniest changes are often significant, yet that does not necessarily mean that the effect is relevant. I.e. in the LAD-same category, the effect size of the changes are within similar range between all categories except for the increase in mRNA counts for the LAD-gain EN category. I therefore also find the statement on lines 180-182 not nuanced enough. I would say that the change in mRNA counts is neglectable for the LAD-gain category.

-It is unclear whether the SON-Go-CaRT data is obtained with GW17 and GW20 stages like for LADs. I am missing a comparison between the replicates to assess overall data quality. I.e. the Jaccard index for Fig. ED4b does not include replicates. Unlike the LAD data in Fig. 1b, the SON data in Fig. 2b appears fragmented and much noisier. An in-debt analysis of data quality is needed to evaluate the profiles. Similarly, the dynamic range of the data plotted in Fig. 2d is very narrow. This usually is an indicator of poor data quality. The y-axis label does not indicate what is plotted here, so I find it hard to assess what the nature of the low dynamic range could be (plus my comments above).

-Fig. ED4a I am surprised genes in LADs are so short. Normally genes in LADs are long, such as the olfactory receptor genes.

-What is the genomic coverage of SPADs per cell type? It appears EN cells have much lower coverage? Is this related to data quality? In general, in such instances, the domain-size is also smaller as domains become more fragmented due to detection limitations. This needs to be shown and addressed. Especially when concluding that the SPADs in EN are very different from IPC and RG.

-For the GO-analyses in Fig.3 it would be good to include only the genes that change in expression upon LAD/SPAD loss or gain between cell types. Many genes with LADs are “bystander” products of spatial genome repositioning, yet are irrelevant to the biological process.

-The conclusion that bivalent genes in LADs are enriched for neurodevelopmental function on lines 270-271 requires a comparison to the entire genome and non-LADs. It is expected that across the genome in RG cells genes with bivalent marks are enriched for neurodevelopmental. This does not necessarily mean anything in relation to LAD function.

-For data interpretation, I suggest to plot the levels of H3K27me3 and H3K4me3 over promoters for the bivalent category stratified by LADs, non-LADs and SPADs. Are the levels of these marks the same? If not, this may also explain why these genes are transcribed at lower levels compared to non-LAD regions. LADs are known to attenuate gene activities in general.

-Fig. 4c the gene expression changes of genes going from bivalent (RG) to monovalent H3K4me3 (EN) is similar for genes that stay within LADs (LAD-same) as for genes that change their spatial location to SPADs or elsewhere. What does that mean for the importance of spatial redistribution of genes for gene regulation? And the overall conclusions related to bivalency in general?

-It would be interesting to plot the re-ChIP experiments in Fig.5 genome-wide. I.e. as a density scatter plot of all bi-valent genes in LADs (H3K4me3 and H3K27me3 on the axis) compared to non-LADs or SPADs.

-In Fig. 6d the data is noisy for LAMINB1 and H3K27me3 and the difference between NPC and iN is very small. Is this related to few genes included in this analysis? I am surprised that genes are identified as LAD-loss if the differences are so small. This may be related to the flanking regions being more LAD-associated in NPC compared to iN, yet the promoters do not. Because of the domain calling, these promoters then become annotated as LAD regions. This is also apparent from the examples in Fig. 4d-e. It appears the promoters of the bivalent genes are not actually themselves associated with the nuclear lamina. How do the authors interpret this, and can we then still talk about spatial repositioning?

-The conclusion of the experiments presented in Fig. 7 is that upon H3K27me3 inhibition gene expression of bivalent genes does not change in LADs. However, it does also not change in non-LADs (Fig. 7c). I therefore do not see the significance of the finding and how it contributes conceptually to interrelationship between LADs and bivalent genes.

Version 1:

Reviewer comments:

Referee #1

(Remarks to the Author)
The reviewers provided new data and analyses and have addressed most of my previous concerns. The new data on mechanistic insight is exciting.

Referee #2

(Remarks to the Author)
The authors have carefully addressed most of my earlier comments, and the study overall provides interesting insights into spatial genome organization. Nevertheless, the data supporting Fig. 6 are too limited in quality to substantiate the conclusions drawn, and I would recommend reconsidering their inclusion in the manuscript. Furthermore, while the work is of clear relevance to the field, I do not believe the findings reach the level of broad conceptual advance expected for Nature more for a specialized journal like Nature Neuroscience.

I find the conclusion that RG LAD-associated genes show reduced Pol II-Ser2 occupancy relative to non-LAD bivalent genes, whereas Pol II-Ser5 does not, insufficiently supported by the data in Fig. 6a. In fact, Pol II-Ser5 signal appears somewhat higher in LADs compared to non-LAD bivalent domains. The observed differences are not particularly convincing and may primarily reflect differences in baseline signal rather than meaningful biological variation.

Expanding the analysis window to ±10 kb upstream and downstream of the gene body might help clarify whether the apparent trends persist beyond the immediate gene region, especially given the current signal appears quite noisy.

Additionally, I am puzzled by the difference in both pattern and baseline levels of Pol II-Ser2 and Pol II-Ser5 in RG samples for LAD-associated genes when comparing Fig. 6a and Fig. 6b. While I understand that the gene selections differ slightly between panels, such discrepancies raise concerns about the consistency of the signal and reduce confidence in the robustness of the conclusions.

To better assess the quality of this dataset, it would be useful to perform a similar analysis across all active and inactive genes. This would help establish the expected differences in Pol II levels and clarify the baseline signal levels.

As it stands, the data quality appears insufficient to robustly support the key conclusions drawn in relation to Fig. 6.

The results shown in Fig. 6c–d are promising and raise interesting hypotheses. Nonetheless, the current analysis appears to be based on single experimental replication and low cell numbers, which weakens the robustness of the conclusions. In particular, the findings related to NELF enrichment are potentially significant but would benefit from supporting genomic evidence to confirm its specific localization at the nuclear periphery and association with bivalent genes.

Version 2:

Reviewer comments:

Referee #2

(Remarks to the Author)
I appreciate the authors' careful and constructive response to my previous concerns. They have addressed the majority of the technical issues I raised, and the revisions have significantly strengthened the robustness of the data presented in Figure 6.

From a conceptual standpoint, I still feel that the scope and focus of the work may be better aligned with a more specialized journal. However, I recognize that this consideration ultimately falls within the editorial remit rather than the reviewer's role.

Open Access This Peer Review File is licensed under a Creative Commons Attribution 4.0 International License, which permits use, sharing, adaptation, distribution and reproduction in any medium or format, as long as you give appropriate credit to the original author(s) and the source, provide a link to the Creative Commons license, and indicate if changes were made.
In cases where reviewers are anonymous, credit should be given to 'Anonymous Referee' and the source.
The images or other third party material in this Peer Review File are included in the article's Creative Commons license, unless indicated otherwise in a credit line to the material. If material is not included in the article's Creative Commons license and your intended use is not permitted by statutory regulation or exceeds the permitted use, you will need to obtain permission directly from the copyright holder.
To view a copy of this license, visit https://creativecommons.org/licenses/by/4.0/

RESPONSE TO REVIEWERS

Summary of major revisions:

We thank the reviewers for their thoughtful and thorough comments on our work. We hope the substantial additions and clarifications described below address the reviewers' concerns, which have significantly strengthened the manuscript.

To address concerns regarding data robustness and reproducibility, we have included additional age-matched replicates of human brain tissue. These new datasets support and reinforce our original findings on LAD-to-SPAD remodeling during neurogenesis and the regulation of bivalent gene expression by spatial organization of the genome (**revised Figs. 1–3; ED Figs. 2-4**).

To directly interrogate the interplay between spatial compartmentalization and H3K27me3 deposition in transcriptional repression at the lamina, we engineered and employed a synthetic LacO-LacI tethering system to recruit reporter plasmids to the nuclear lamina. These experiments demonstrated that lamina association alone is sufficient to strongly repress transcription, independently of H3K27me3 (**Fig. 5**). Furthermore, we repeated our functional perturbation studies by performing EZH2 inhibition in an independent model system – iPSC-derived NPCs. These experiments further substantiate our conclusion that the poised, low-expression state of LAD-associated bivalent genes is not primarily governed by H3K27me3, but rather by their subnuclear positioning at the lamina (**Fig. 5, ED Fig. 10**). Together, our genomic and plasmid tethering experiments reinforce that spatial genome positioning – rather than H3K27me3 – is the primary regulator of bivalent gene expression.

To further address the reviewers' requests for a more mechanistic basis to our findings, we provide new evidence that lamina-associated gene repression involves spatial segregation of transcriptional machinery, including active forms of RNA Pol II and transcription elongation factors. Bivalent genes within LADs show reduced elongation-competent Pol II occupancy, suggesting lamina proximity acts as a spatial barrier to productive transcription. During RG to EN differentiation, only bivalent genes that leave LADs and become H3K4me3-monovalent show strong Pol II recruitment, underscoring the functional role of genome repositioning in transcriptional activation (**Fig. 6, ED Fig. 10**).

Collectively, these new results strengthen our central conclusion that spatial genome architecture plays a primary role in regulating bivalent chromatin states during human brain development. We believe the revised manuscript now presents a more mechanistically grounded and biologically insightful view of how subnuclear genome compartmentalization contributes to neurodevelopment. The principles uncovered – particularly the interplay between subnuclear genome compartmentalization and local chromatin states – have broad implications for gene regulation in development and disease.

Referees' comments:

Referee #1 (Remarks to the Author):

In this manuscript, Ahanger and colleagues characterized the remodeling of subnuclear genome compartmentalization during neurogenesis by examining the genomic regions marked by LaminB1 and SON in RG, IPC, and EN using the developing human cortex. They further demonstrated that during neurogenesis, bivalent genes—co-marked by H3K4me3 and H3K27me3—relocate to speckles, resulting in increased gene expression, particularly for neurodevelopmental genes. Additionally, they concluded the key factor in maintaining the lowly expressed, poised state of bivalent genes is not H3K27me3. This is nice study, well-written, and the functional validation is nice convincing. There are a number of issues need to be addressed.

We thank the reviewer for their thoughtful and constructive feedback and are pleased that they found our work of interest, well-written and supported by convincing functional validation. In response to the reviewer's comments, we have substantially strengthened the manuscript by incorporating several new experiments and analyses, including those specifically suggested by the reviewer. Collectively, these new results strengthen our central conclusion that spatial genome architecture plays a primary role in regulating bivalent chromatin states during human brain development. Below, we detail our responses to each of the reviewer's comments.

Major comments:

1. The authors concluded that 'the LaminB1 profiles from RG, IPC, and EN populations were highly similar between GW17 and GW20 (Fig. 1a, Extended Data Fig. 3a), indicating that these developmental time points are reasonable biological replicates for studies of LAD architecture'. Throughout the study, GW17 and GW20 were treated as replicates. However, there are notable differences in the correlation matrix of LaminB1 (Extended Data Fig. 3a), which suggests they may not be suitable as replicates. For instance, the correlation between IPC-GW17 and IPC-GW20 is 0.69, which represents the 'same group', but the correlation between RG-GW17 and IPC-GW17 (different groups) is actually higher at 0.72-. As a reference, when interpret the results from SON, the authors considered a Jaccard index of 0.59 as similar (Extended Data Fig. 4b), further raising questions about the validity of using these time points as replicates.

We have strengthened the statistical rigor underlying our conclusions by obtaining additional biological replicates for both GW17 and GW20. These new data allowed us to generate true biological replicates at these two time points. LAD profiles from GW17 RG, IPC and EN showed strong concordance between replicates (Pearson correlation 0.72-0.94; **reviewer Fig. 1a**). Moreover, LAD profiles were largely similar between RG and IPC according to Jaccard index calculations for LAD profiles called on the median of the two biological replicates (**reviewer Fig. 1b**) and PCA analysis of LaminB1 GO-CaRT (**reviewer Fig. 1c**). Thus, for analyses of LAD dynamics during neurogenesis, we focused on comparisons between RG and EN.

Importantly, our main conclusions remain unchanged. Analyses based solely on GW17 biological replicates confirm the extensive remodeling of LADs and SPADs during the RG to EN differentiation, as well as the strong association between spatial genome positioning and the regulation of bivalent chromatin states (**revised manuscript Figs. 1–3**). Further, analysis of two biological replicates each at GW17 and GW20 revealed that the LAD architectural changes observed at GW17 are also essentially the same at this later developmental stage (**ED Figs. 2,**

3, 4c), supporting the conclusion that LAD remodeling is a robust feature of human neurogenesis.

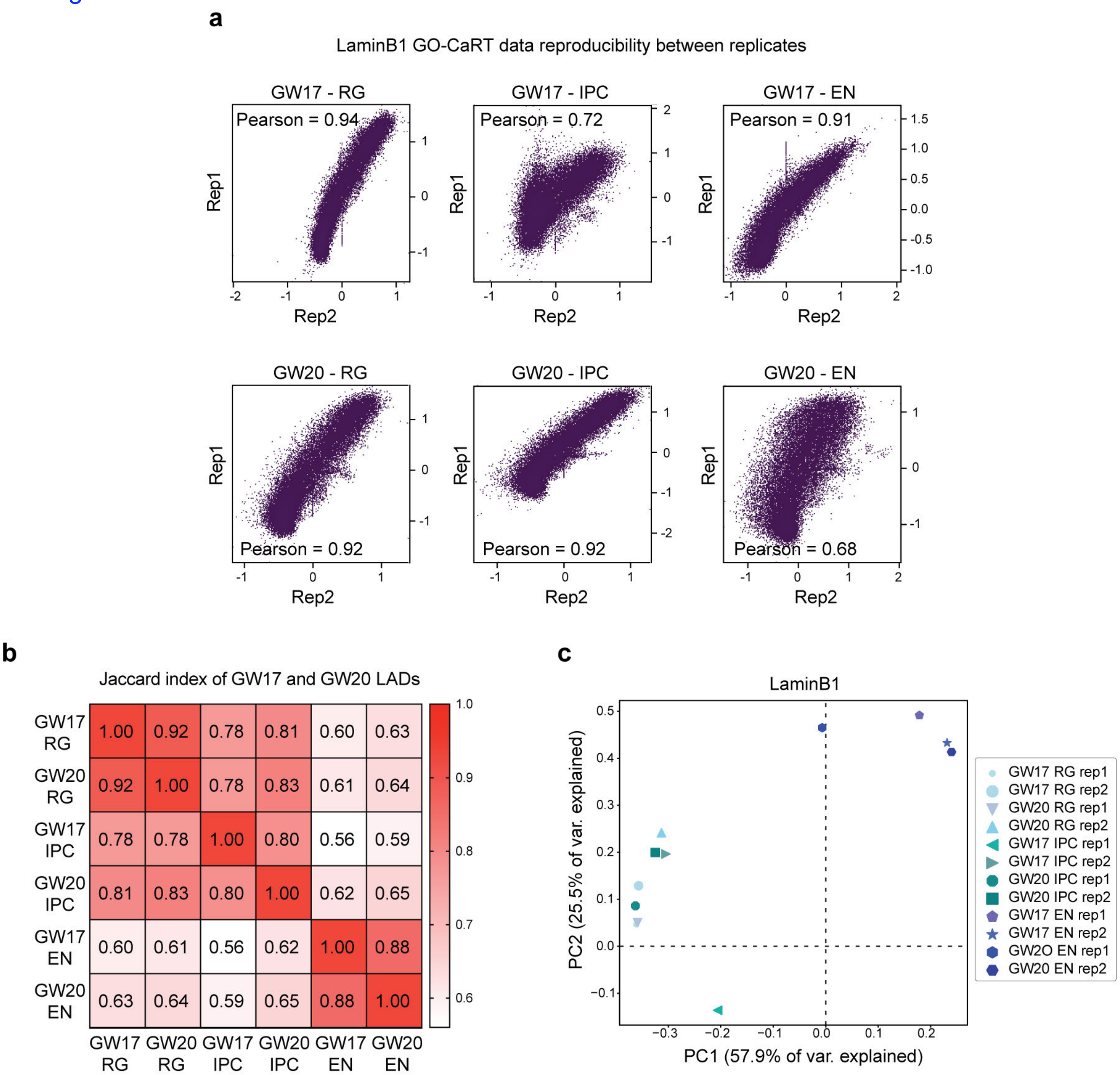

Reviewer Response Figure 1. a, Pearson correlation of LaminB1 GO-CaRT profiles of RG, IPC and EN isolated by FANS from GW17 and GW20 brain cortex. **b,** Jaccard similarity index of LADs in RG, IPC and EN at GW17 and GW20. **c,** PCA plot of LaminB1 GO-CaRT profiles of RG, IPC and EN at GW17 and GW20.

2. As indicated, the LaminB1 and SON regions were identified at scales ranging from ~Kb to Mb, while the histone-marked regions were identified at much smaller scales, from hundreds to thousands of base pairs. This difference in resolution makes it tricky to accurately identify overlapping regions, especially when calling the differential regions (gain, loss, same). The authors should make this clear and use the same standard throughout the study.

We apologize for any confusion about the relationship between subnuclear compartment-associated genomic domains and smaller peaks of histone modification. We have modified the

methods section to clarify the methodology for LAD-loss/gain/same regions and histone-marked peaks. As the reviewer notes, LAD-loss/gain/same domains are large (kb-Mb-scale) regions, while the histone-marked peaks are indeed much smaller. In our comparative analyses, we are asking which of these small peaks exist within the different subsets of compartment-associated broad domains (LAD-loss, etc.). We define the inclusion/non-inclusion of that overlap by restricting our analyses to the peaks present at gene TSS. Thus, genes that have been classified as containing peaks of H3K4/27me3 are stratified by LAD status (LAD, nonLAD, LAD-loss, etc.) according to the location of their TSS. Importantly, the differences in methodology between LAD domain calling and histone peak calling reflect the distinct biological nature of these two epigenomic phenomena and are consistent with methods widely used in the field.

3. As we know, large domain which usually contain more than one gene, how the author chooses the associated genes for gene expression level and GO term analysis? And what is the rational for that?

For both gene expression and gene ontology (GO) term enrichment analyses, we defined LAD- or SPAD-associated genes as those whose promoter regions—defined as ±1 kb around the transcription start site (TSS)—overlapped with LADs or with SPADs. By using TSS overlap with LAD/SPAD domains, we aimed to reduce ambiguity arising from genes located entirely/or partially within large domains but whose regulatory elements may not be spatially associated with the nuclear compartment in question. This promoter-based criterion is widely accepted in the field as a functionally relevant window for regulatory influence on transcription, as it captures proximal chromatin contacts that most directly influence transcription. This approach has been used in our previous work (Ahanger *et al.,* Nat. Neurosci. 2021) and is consistent with published studies from other groups (e.g., Guelen *et al*., Nature 2008*;* Peric-Hupkes *et al.,* Mol. Cell 2010). This has been clarified in the revised manuscript under the Methods section.

4. The authors make a strong conclusion the dominant role of spatial genome positioning in regulating bivalent gene expression, however, the data presented does not fully support this claim. For example, in figures 4a and 6c, genes within H3K4me3 group consistently exhibit higher expression levels than those in the bivalent and H3K27me3 groups, regardless of whether those genes are located within LADs, non-LADs/non-SPADs, or SPADs. In an extreme case, H3K4me3 only genes within LADs express at a higher level than bivalent genes within SPADs. Does this suggest chromatin state plays a more significant role, while genome positioning serve as a "tuning" mechanism in transcriptional regulation?

We appreciate the reviewer's thoughtful comments. We would like to clarify that our original statement of a "dominant role" for the lamina was meant to refer only to the case of bivalent genes and their repressed, "poised" state, and was not meant to draw any conclusions about the relative "significance" of genome positioning vs. chromatin state in overall transcriptional regulation. Our data (**Fig. 3c**) suggest that lamina localization is not a "tuning mechanism" in the case of bivalency resolution, as the influence of LAD-loss on transcriptional induction upon bivalency resolution was quite dramatic compared to all other spatial categories. However, for other aspects of local chromatin state, we agree that spatial location may indeed serve more of a tuning function. We have clarified these points in the revised manuscript. To strengthen our central claims about the lamina and bivalency, we have performed several new experiments that illustrate how lamina association maintains repression of bivalent genes:

A) Enhanced statistical power for EZH2 inhibition experiments in NPCs
First, we repeated our EZH2 inhibition experiments in NPCs derived from an iPSC line (**Fig. 5a-d, ED Fig. 10a-b**). As compared to ESC-derived NPCs, iPSC-derived NPCs have a greater number of bivalent genes across the genome, which enhanced statistical analyses. As previously observed in ESC-derived NPCs, EZH2 inhibition led to a global loss of H3K27me3 in iPSC-derived NPCs but did not alter LAD architecture. Importantly, bivalent genes within LADs remained transcriptionally repressed despite the depletion of H3K27me3. These results demonstrate that lamina association plays a strong role in maintaining repression of bivalent genes independently of H3K27me3.

B) Functional tests of H3K27me3 in lamina-associated repression with plasmid reporters
To directly interrogate the interplay between lamina association and H3K27me3 deposition in transcriptional regulation, we engineered a LacI-LacO-based system to tether plasmid DNA reporters to the lamina. We generated a LacI-LaminA (LmnA) fusion protein to tether plasmid DNA containing an array of LacO sequences and a red fluorescent protein (RFP) reporter gene driven by the EF-1α promoter. We confirmed successful tethering of pLacO-plasmids to the lamina by LacI-LmnA by microscopy. Importantly, lamina tethering strongly expressed mRNA expression from the plasmid.

We next employed EZH2 inhibition to determine whether H3K27me3 contributes to transcriptional repression induced by lamina tethering. EZH2i effectively depleted H3K27me3 from plasmid DNA (ChIP) and did not affect the localization of reporter plasmid to the nuclear lamina by LacI-LmnA. In cells expressing LacI, EZH2i increased RFP reporter expression. In contrast, EZH2i failed to derepress RFP expression in cells expressing LacI-LmnA. These findings demonstrate that localization of DNA to the nuclear lamina represses transcription independently of H3K27me3. These results are presented in the revised manuscript under the section “Nuclear lamina-tethered DNA constructs are repressed independent of H3K27me3” and shown in **Fig 5e-l and ED Fig. 10c-e**.

Collectively, our genomic analyses and plasmid tethering experiments strongly support a model in which nuclear lamina association is a critical regulator of the transcriptionally repressive state of LAD bivalent genes, acting independently of local H3K27me3.

C) Spatial segregation of Pol II regulators limits bivalent gene transcription at the lamina:
To provide insight into the molecular mediators of lamina-associated transcriptional repression, we considered the possibility that lamina-associated transcriptional repression involves spatial segregation of active RNA Pol II and its regulators. We profiled the genome-wide occupancy of the initiating (Ser5P) and elongating (Ser2P) forms of Pol II during GW17 neurogenesis. RG LAD genes exhibited markedly lower levels of Pol II Ser5P and Ser2P as compared non-LAD genes (**ED Fig. 10f-g**).

Notably, bivalent genes in RG LADs showed reduced occupancy of elongating Pol II Ser2P relative to non-LAD bivalent genes, whereas Pol II Ser5P levels were similarly low at LAD and non-LAD bivalent gene promoters (**Fig. 6a**). Importantly, bivalent genes in LAD-loss regions, compared to bivalent genes genome-wide, showed greater increase in Pol II enrichment in EN, particularly for the elongating Ser2P form across the gene body (**Fig. 6b, ED. Fig. 10h-i**). These data suggest that proximity to the nuclear lamina restricts transcriptional elongation of bivalent genes.

To investigate whether the local environment of the nuclear lamina excludes transcriptional machinery necessary for productive transcription, we analyzed the subnuclear localization of Pol II regulatory cofactors involved in elongation. CDK9 and Cyclin T1 (positive transcription elongation factor P-TEFb subunits) – and ENL (super elongation complex), were depleted from the nuclear periphery and enriched in the interior (**Fig. 6c-d**). In contrast, the negative elongation factor NELF-B was relatively enriched at the lamina (**Fig. 6c-d**). Together,

these findings support a model in which the nuclear lamina acts as a repressive environment by spatially segregating components essential for productive transcription. These new data have been incorporated into the revised manuscript in the subsection "Spatial segregation of Pol II regulators limits transcription of bivalent genes at the lamina" and shown in **Fig. 6 and ED Fig. 10f-i**.

5. Probably the strongest evidence presented in this manuscript to support the claim that spatial positioning plays a more important role than chromatin state in determining bivalent gene transcription comes from the EZH2 inhibition experiments. The results show that H3K27me3 depletion will not disrupt global LAD architecture and low-expression state of genes remained in LADs. However, it is a possible that other repressive chromatin modifications, like H3K9me2/3, are still present in these bivalent genes and are unaffected by H3K27me3 depletion. In this scenario, gene repression could still be attributed to chromatin state rather than solely to 3D genome positioning. Therefore, the authors have not fully disentangled the interaction between spatial positioning and chromatin states and their respective contributions to gene transcription.

The reviewer raises an interesting question which we worked to address experimentally. To assess the role of H3K9me2, we used inhibitors of G9a/GLP, the methyltransferase complex primarily responsible for H3K9me2 deposition. iPSC-derived NPCs were treated with DMSO, A366 (a selective G9a/GLP inhibitor), or a combination of A366 (2 μM) and EZH2 inhibitor (1 μM) (dual inhibition, **reviewer Fig. 2a**). The drug doses used were the maximum concentrations tolerated without substantial cell death.

In DMSO-treated NPCs, we observed a strong positive correlation between LaminB1 and H3K9me2 signals (Spearman = 0.63, **reviewer Fig. 2b**), confirming substantial overlap between LADs and H3K9me2-enriched regions. Treatment with A366 resulted in focal depletion of H3K9me2 at discrete genomic loci, primarily within LADs (**reviewer Fig. 2c)**. These same regions also exhibited H3K9me2 loss under dual inhibition (**reviewer Fig. 2c)**. Incomplete reduction of H3K9me2 was likely due to incomplete G9a/GLP inhibition by A366, as higher drug concentrations induced massive cell death. Although global LAD architecture remained largely unchanged following G9a/GLP or dual inhibition (**reviewer Fig. 2d, e)**, regions with reduced H3K9me2 exhibited decreased LaminB1 signal (**reviewer Fig. 2f)**, suggesting weakened genome-lamina interactions at these sites. Gene expression within these H3K9me2-depleted regions was modestly upregulated following G9A/GLP or dual inhibition. (**reviewer Fig. 2g)** These findings suggest that reducing H3K9me2 contributes to genome-lamina interactions in select LAD regions. However, given the tight coupling between H3K9me2 and lamina association, it remains challenging to determine whether the observed modest transcriptional derepression with G9a/GLP inhibition is a direct consequence of H3K9me2 loss or an indirect effect of weakened lamina interactions. Recent work supports the latter conclusion as detachment of H3K9me2-marked heterochromatin from the nuclear periphery upregulates gene transcription without decreasing H3K9me2 levels (*Marin et al.,* Nat. Cell Biol. *2025*).

[Figure Redacted]

Reviewer Response Figure 2. a, Schematic of G9A/GLP inhibition or G9A/GLP+EZH2 dual inhibition experiments in human iPSC-derived NPCs. **b,** Spearman correlation of LaminB1 GO-CaRT and H3K9me2 CUT&RUN profiles in NPCs. **c,** Genome browser tracks of H3K9me2 CUT&RUN and LaminB1 GO-CaRT in DMSO vs. inhibitor treated cells. **d, e** Spearman correlation of LaminB1 GO-CaRT in DMSO vs. inhibitor treated NPCs. **f,** LaminB1 signal at genes in H3K9me2-depleted regions in DMSO vs. inhibitor treated cells. **g,** Genome browser tracks of H3K9me2 CUT&RUN, LaminB1 GO-CaRT and RNA-seq in DMSO vs. inhibitor treated cells at example genes located in H3K9me2-depleted regions.

6. Since H3K9me2/3 are highly enriched in the LADs and play critical roles in LADs architecture and gene repression, the author could perform H3K9me2/3 CUT&RUN in RG, IPC and EN. It would be insightful to see how H3K9me2/3 changes during neurogenesis and how those changes relate to LAD reorganization and gene transcriptional changes. Additionally, the author might consider including DNA methylome profiling to the dataset, as DNA methylation is another crucial repressive modification in LADs and interacts extensively with H3K27me3.

We thank the reviewer for encouraging us to think more deeply about the basis of lamina-associated transcriptional repression. We performed CUT&RUN profiling of H3K9me2 in RG and EN populations. Consistent with previous studies (Ahanger *et al.* 2021 Nat. Neurosci; Guelen *et al.* 2008 Nature), H3K9me2 was broadly enriched within LADs across the human neurogenic lineage (**reviewer Fig. 3a**), and H3K9me2 and LaminB1 domain calls had moderately high correlation (Jaccard index 0.74 for RG, 0.63 for EN). Next, we examined H3K9me2 enrichment specifically in regions that undergo LAD remodeling during RG to EN differentiation. In LAD-loss regions, H3K9me2 levels were decreased in EN, corresponding to the strong increase in gene expression (**reviewer Fig. 3c**). Interestingly, in LAD-gain regions there was only a marginal increase in H3K9me2 levels in EN (**reviewer Fig. 3c**), but gene expression was still strongly repressed. We also identified genomic regions that exhibited binary changes in H3K9me2 levels – gains, losses, or no change – during differentiation from RG to EN. Like LAD-gain, H3K9me2 gain was associated with a mild decrease in gene expression (**reviewer Fig. 3b**). However, in contrast to LAD-loss, H3K9me2 loss did not correlate with a significant increase in gene expression (**reviewer Fig. 3b**). These findings together suggest that the dynamic spatial localization of genes to the lamina contributes to transcriptional repression without the need for high levels of H3K9me2. These data have been incorporated into the revised subsection, "Global LAD architecture is extensively remodeled during human cortical neurogenesis," and are presented in **ED Fig. 4a-b**.

With regards to DNA methylation, we were unable to perform such experiments due to limited tissue availability. Further, we and others (Ahanger *et al.* 2021 Nat. Neurosci; Berman *et al.* 2012 Nat. Genet.) have shown that DNA methylation is generally depleted in LADs, making it an unlikely candidate for mediating lamina-associated transcriptional repression.

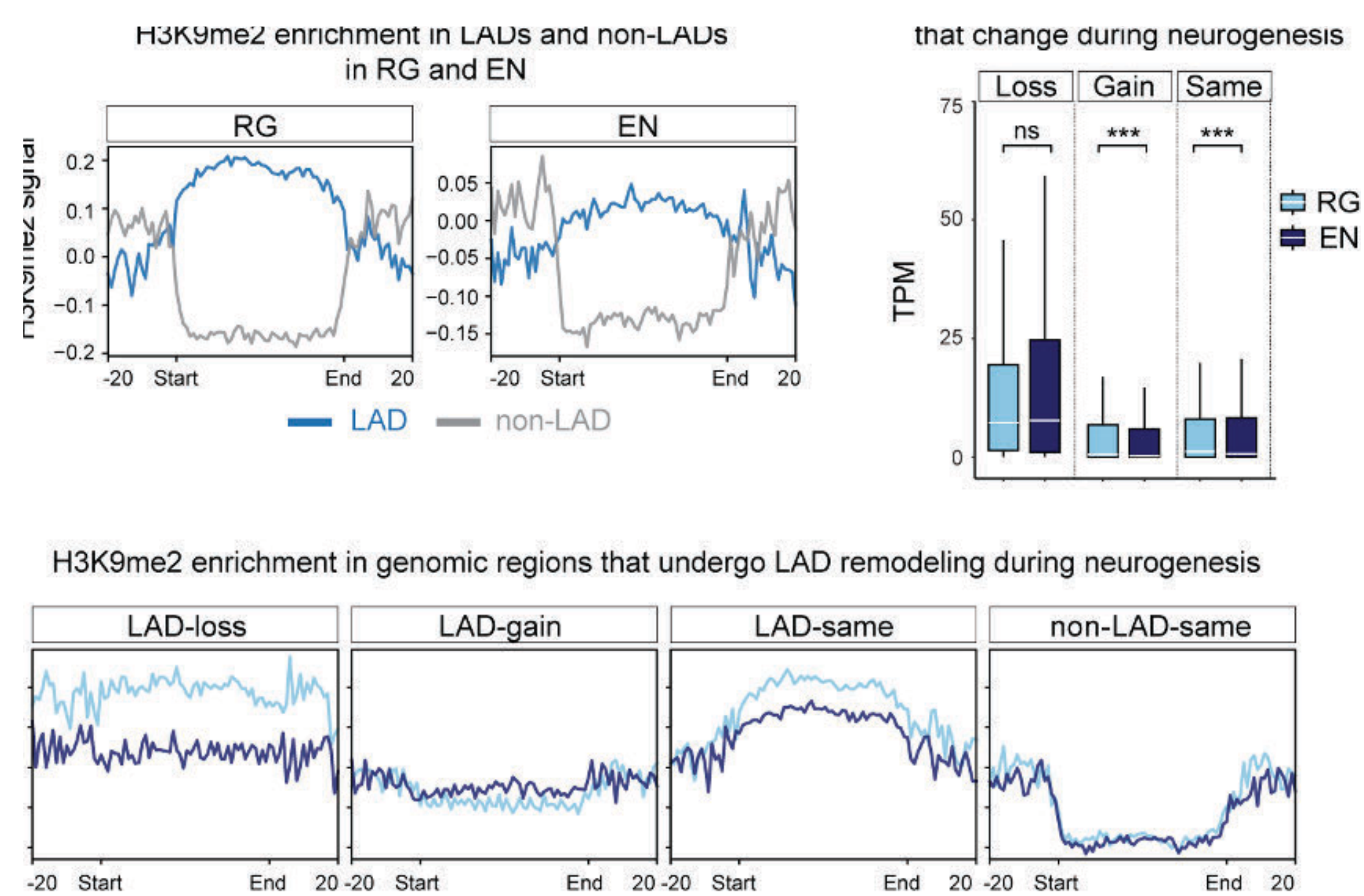

Reviewer Response Figure 3. a, Metagene plot showing H3K9me2 enrichment in LADs and non-LADs in RG and IPC. **b,** Gene expression in genomic regions that undergo changes in H3K9me2 domains. **c,** Metagene plot depicting H3K9me2 enrichment in LAD-loss, LAD-gain, LAD-same regions and non-LAD-same regions in RG and EN.

7. It is not very clear how the LADs and SPADs change during neurogenesis. For example, how many LADs exist in all three cells? how many LADs only exist in RG? how many LADs only exist in EN? how many LADs lost in IPC and then regained in EN. They author could use a figure to summarize these changes.

We have now clarified how LADs and SPADs change during neurogenesis. As noted in our response to Reviewer Comment #1, we assessed LAD dynamics by comparing profiles across RG and EN.

Below, we provide the coverage and specificity of LADs and SPADs identified in each cell type, summarized in **reviewer Fig. 4**:

RG Total LADs = 1284 Mb; RG-specific LADs = 65 Mb (5.1% of all RG LADs)
IPC Total LADs = 1211 Mb; IPC-specific LADs = 57 Mb (4.7% of all IPC LADs)
EN Total LADs = 1459 Mb; EN-specific LADs = 373 Mb (25.6% of all EN LADs)
LADs shared between RG, IPC, and EN = 899 Mb
LADs lost in IPC and regained in EN = 126 Mb

RG Total SPADs = 643 Mb; RG-specific SPADs = 174 Mb (27.1% of all RG SPADs)
IPC Total SPADs = 581 Mb, IPC-specific SPADs = 116 Mb (19.9% of all IPC SPADs)
EN Total SPADs = 380 Mb; EN-specific SPADs = 329 Mb (86.6% of all EN SPADs)

We would also like to emphasize that due to the inherent limitations and variability of domain calling algorithms for LADs/SPADs, we believe that percent genome coverage (which is used in the manuscript text/figures) offers a more reliable metric than domain counts.

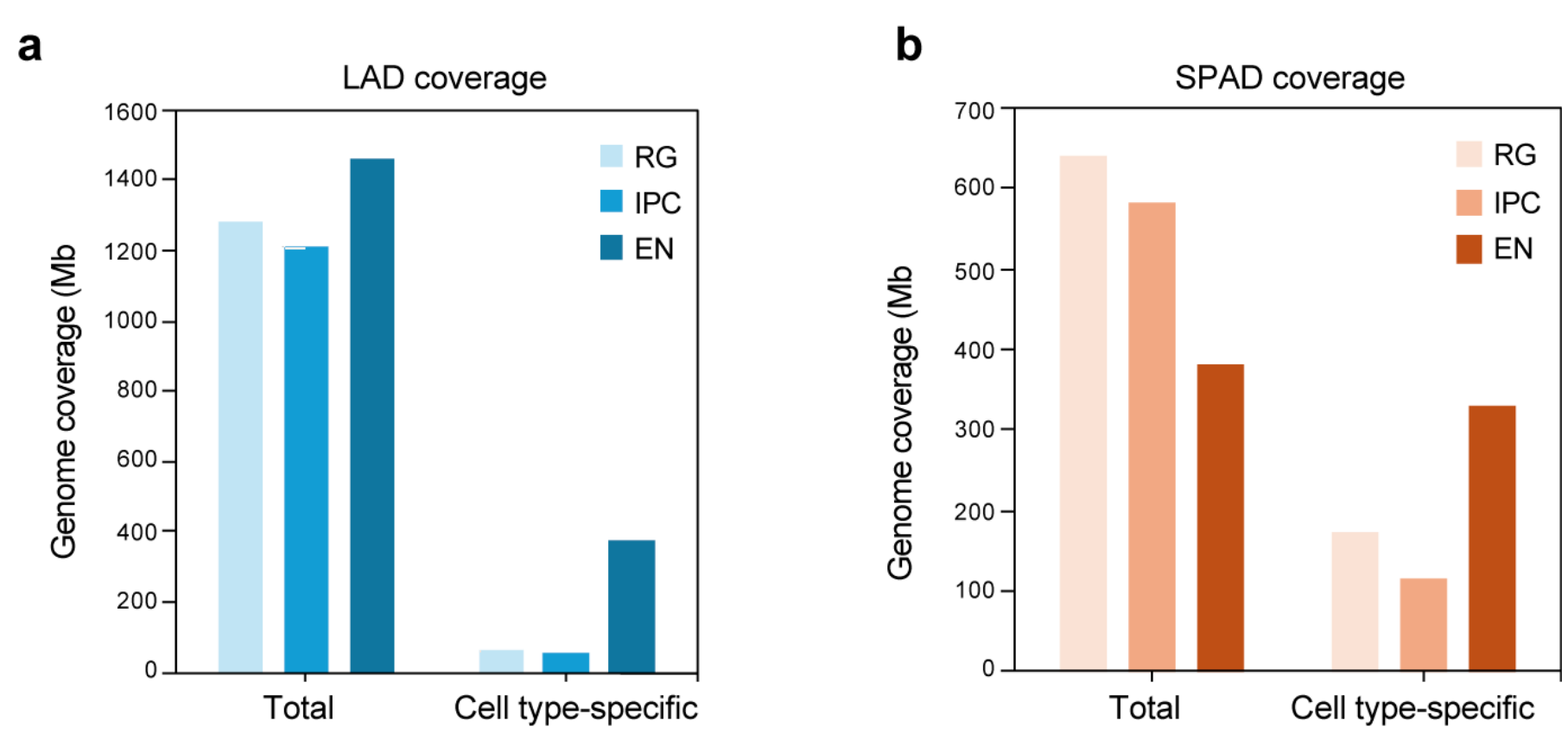

Reviewer Response Figure 4. Bar plots showing genomic coverage of total and cell type-specific LADs (**a**) and SPADs (**b**) at GW17.

8. The authors have not clearly shown the percentage of bivalent genes (as well as H3K4me3-only and H3K27me3-only genes) that are located within LADs, non-LADs/non-SPADs, and SPADs in the three cell types. This data would help readers better perceive to what extent bivalent genes are regulated by LADs.

To clarify the extent to which bivalent, H3K4me3-only, and H3K27me3-only genes are associated with different nuclear compartments, we have quantified the percentage of these gene categories located within LADs, SPADs, and non-LAD/non-SPAD regions in RG. This analysis revealed that bivalent genes are moderately overrepresented in LADs compared to H3K4me3-monovalent, H3K27me3-monovalent, and all genes. The results are summarized in **reviewer Fig. 5** below and have been added to the revised manuscript (**ED Fig. 7c**).

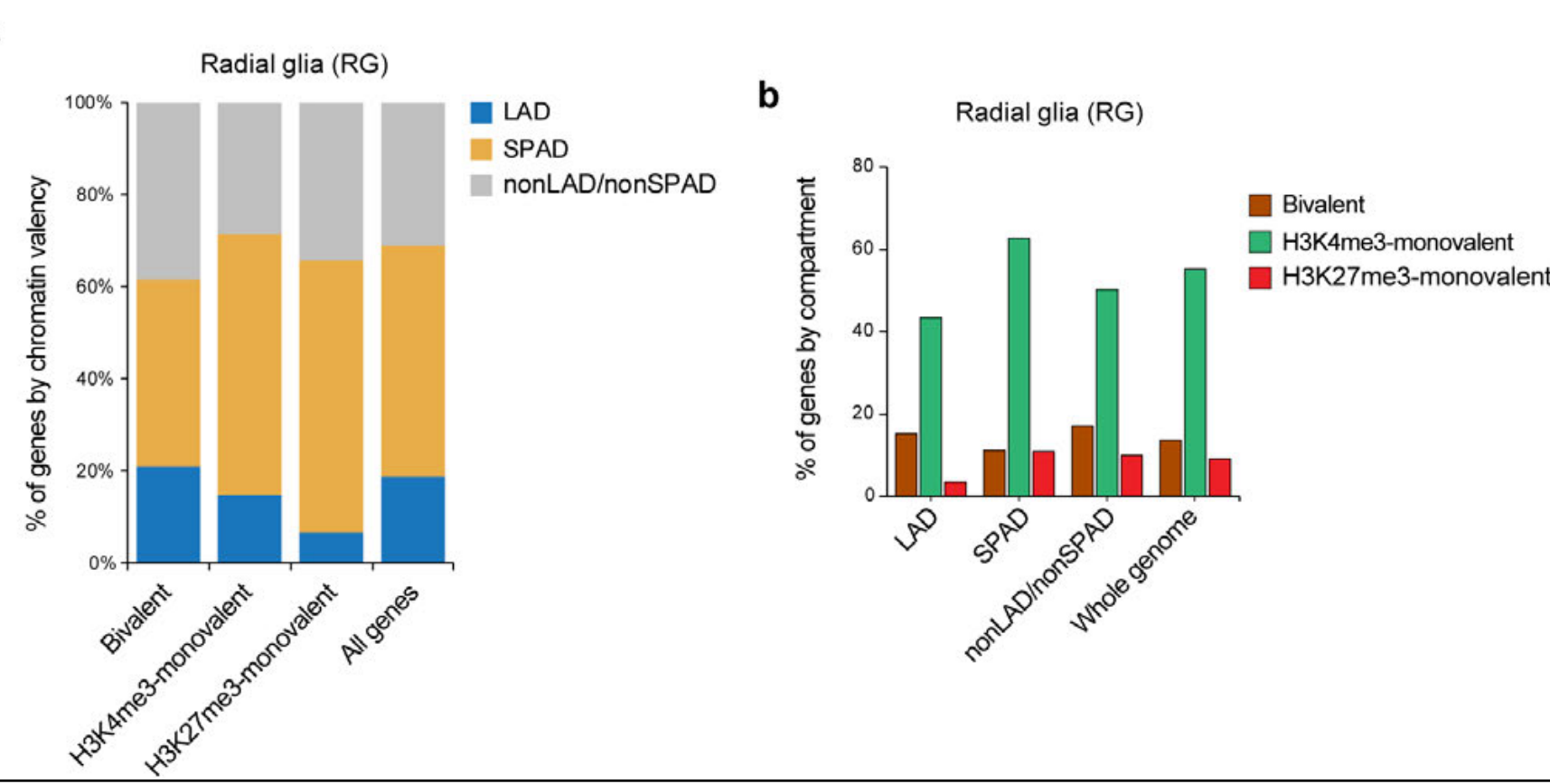

Reviewer Response Figure 5. a, b, Bar plots showing percentage of genes stratified by chromatin state in different genomic compartments in RG.

9. One observation from the example cases is that the bivalent regions marked by H3K4me3 and H3K27me3 were depleted in LaminB1/SON domains, as shown in Fig. 4d, Fig. 5d, and Fig. 7d. Is this depletion a common feature throughout the genome? If so, do the authors have any thoughts on this pattern?

To address the reviewer's point regarding local depletion of LaminB1 levels around bivalent promoters, we quantified LaminB1 signal within 300 kb of the transcription start site (TSS) for LAD-associated genes, stratified by chromatin state (**reviewer Fig. 6**). This analysis showed that the reduced LaminB1 signal near bivalent promoters is not unique to this group, but represents a broader pattern seen across all LAD-associated genes, regardless of chromatin state. As a control, genes located in non-LAD regions showed no LaminB1 enrichment, as expected. Importantly, the degree of this depletion varies by chromatin context: genes marked by H3K4me3 exhibit the most pronounced reduction in LaminB1 signal, followed by bivalent genes and H3K27me3-monovalent genes. These findings suggest that local variation in lamina association – reflected by LaminB1 enrichment – alongside promoter chromatin state, may modulate transcriptional activity within LADs, and help explain why some H3K4me3-marked LAD genes remain highly expressed.

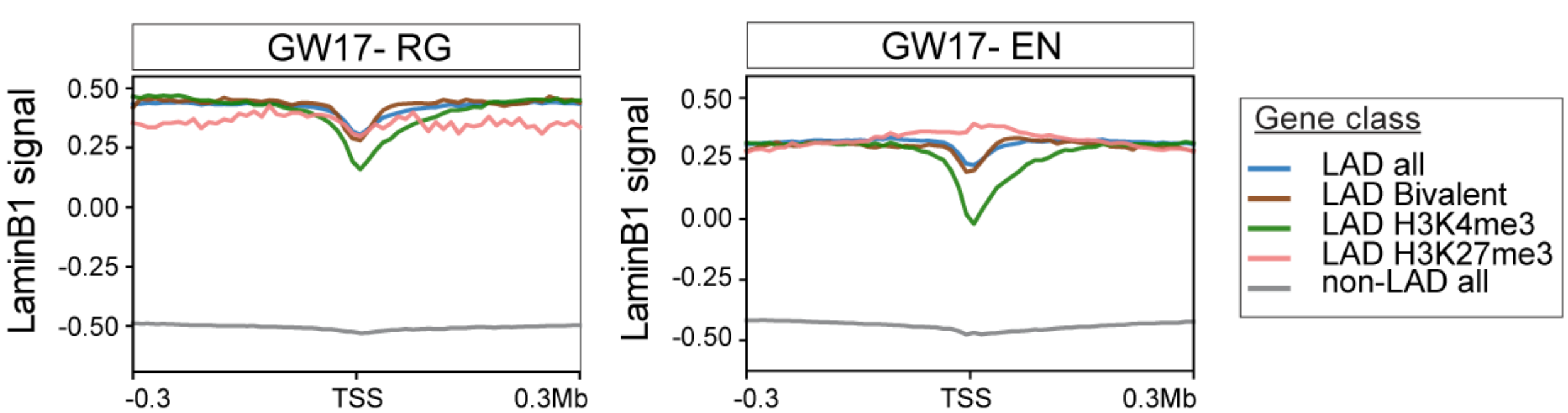

Reviewer Response Figure 6. Metagene plots of normalized LaminB1 signal centered around TSS for LAD genes stratified by chromatin state in RG and EN. LaminB1 signal for non-LAD genes is shown for comparison.

10. The authors used co-staining with DNA-FISH and LaminB1/SON to validate the movement of genes from the lamina to speckles. However, this measurement should be conducted in 3D rather than 2D (Fig. 3) for more accurate results.

We would like to clarify that the DNA-FISH measurements shown in Fig. 3e–g (**revised Fig. 2e-h**) were indeed conducted in 3D. Specifically, we performed three-dimensional image reconstruction from confocal z-stacks using Imaris x64 software (v9.2.1, Bitplane) to accurately determine gene positioning relative to nuclear landmarks, including the nuclear lamina (LaminB1) and nuclear speckles (SON). Further methodological details are provided in the Methods section under the subheading “DNA-FISH combined with immunocytochemistry and image analyses.”

11. In Fig 4C, the authors focused their analysis on bivalent regions at the RG state, but what about regions that become bivalent at the EN state? Exploring these regions could provide additional insights.

As suggested by the reviewer, we analyzed bivalency in EN at GW17. Genome-wide, we identified 2742 bivalent genes in EN, of which 1045 were specific to EN and 1697 were shared between RG and EN. We further provide a breakdown of genes by compartment and valency in EN (**reviewer Fig. 7a)** as we did for RG in comment #8. To determine whether EN-specific bivalent genes were enriched for any particular biological processes, we performed GO analyses. RG-only bivalent genes were enriched for terms related to neurodevelopment and neurogenesis (**reviewer Fig. 7b**). By contrast, EN-only bivalent genes were enriched for more general developmental processes, but not for neurodevelopment-related pathways (**reviewer Fig. 7c**). A similar trend was observed for the subset of EN-LAD bivalent genes, which also lacked enrichment for neurodevelopmental terms (**reviewer Fig. 7d**).

In addition, it would be useful to examine the changes in expression levels for genes within the same Lamin/SON domain but with different H3K4me3/H3K27me3 features.

As suggested by the reviewer, we additionally examined changes in gene expression levels for all permutations of chromatin state and subnuclear compartment change in RG and EN (**reviewer Fig. 7e and Fig. 3c**). Even after including all permutations of compartment changes, LAD-loss/LAD-to-SPAD switching still has the most dramatic influence on gene expression during neurogenesis.

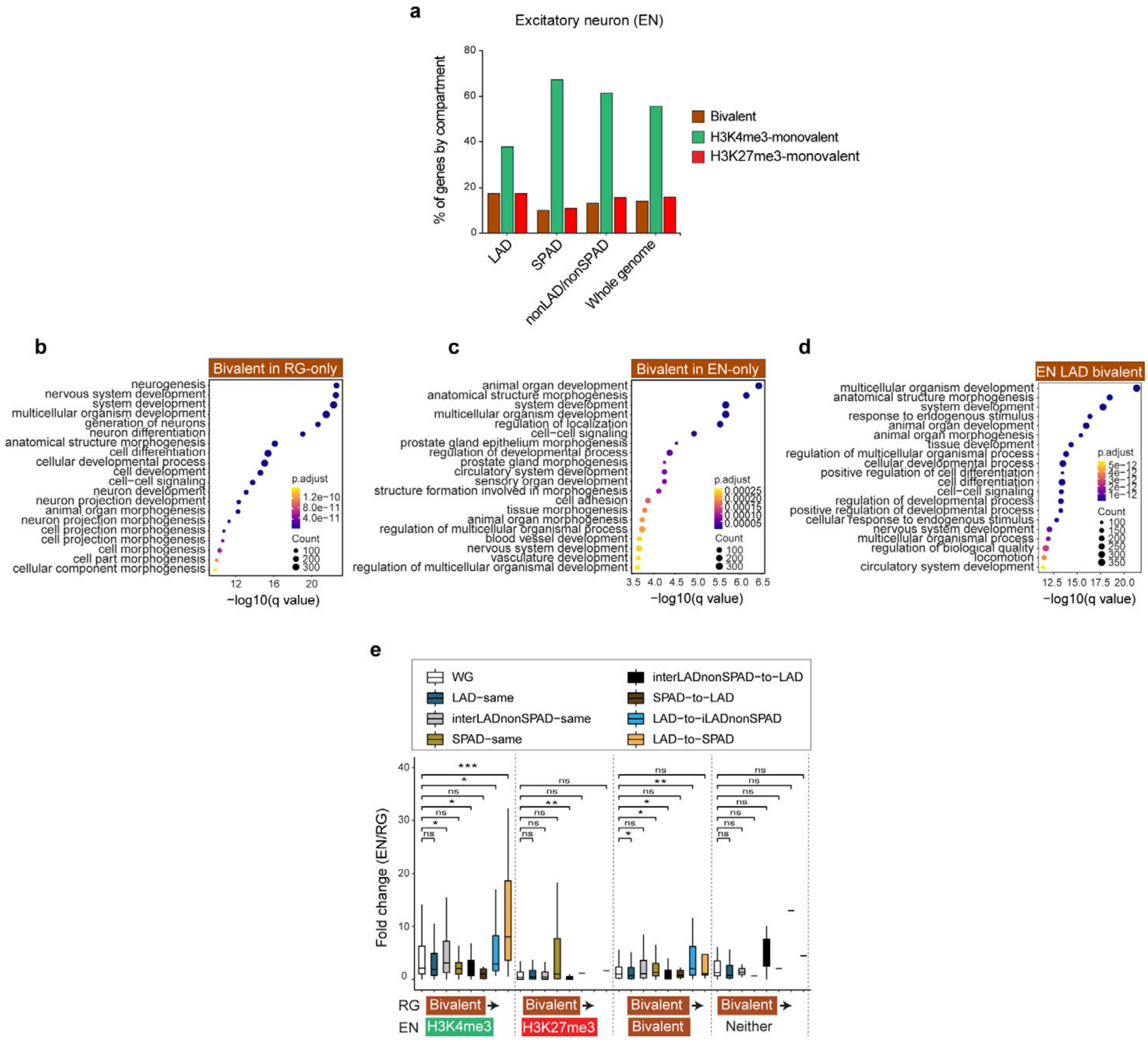

Reviewer Response Figure 7. a, Bar plot showing percentage of genes stratified by chromatin state in different genomic compartments in EN. **b-d,** GO analyses of genes that are bivalent in RG-only (**b**), bivalent in EN-only (**c**) and bivalent in EN LADs (**d**). **e,** Fold change of expression of bivalent genes that resolve to the H3K4me3-monovalent, H3K27me3-monovalent, remain bivalent, or lose both marks during RG to EN differentiation stratified by LAD to SPAD dynamics.

Minor points:

1. There is a typo in Fig. 4a and Extended Data Fig. 3c, which labeled “log1pTPM”, maybe should be “log10 TPM”.

In the revised manuscript we have used log10 TPM in Fig. 4a (now **Fig. 3a**) and Extended Data Fig. 3c (now **ED Fig. 2d**).

2. The authors separately displayed the LADs and SPADs regions in figure 1 and figure 2 and noted that they are “largely mutually exclusive”. However, it is challenging for readers to

compare those figures visually. The authors could consider combining LADs and SPADs in a single figure, and labeling them with different colors, and using with another color for overlapping regions. Meanwhile, they should provide the percentage of overlap between LADs and SPADs in the three cells.

In response, we provide a new figure (**reviewer Fig. 8**) that overlays LADs and SPADs across all chromosomes. LADs and SPADs are labeled in distinct colors, with overlapping regions highlighted in a third color to facilitate direct comparison. In addition, we have quantified the extent of overlap between LADs and SPADs for each cell type. The degree of overlap is negligible in RG (1 Mb, 0.1% of RG LADs) and IPC (15 Mb, 1.2% of IPC LADs). There is a greater degree of overlap in EN (146 Mb, 10.1% of EN LADs).

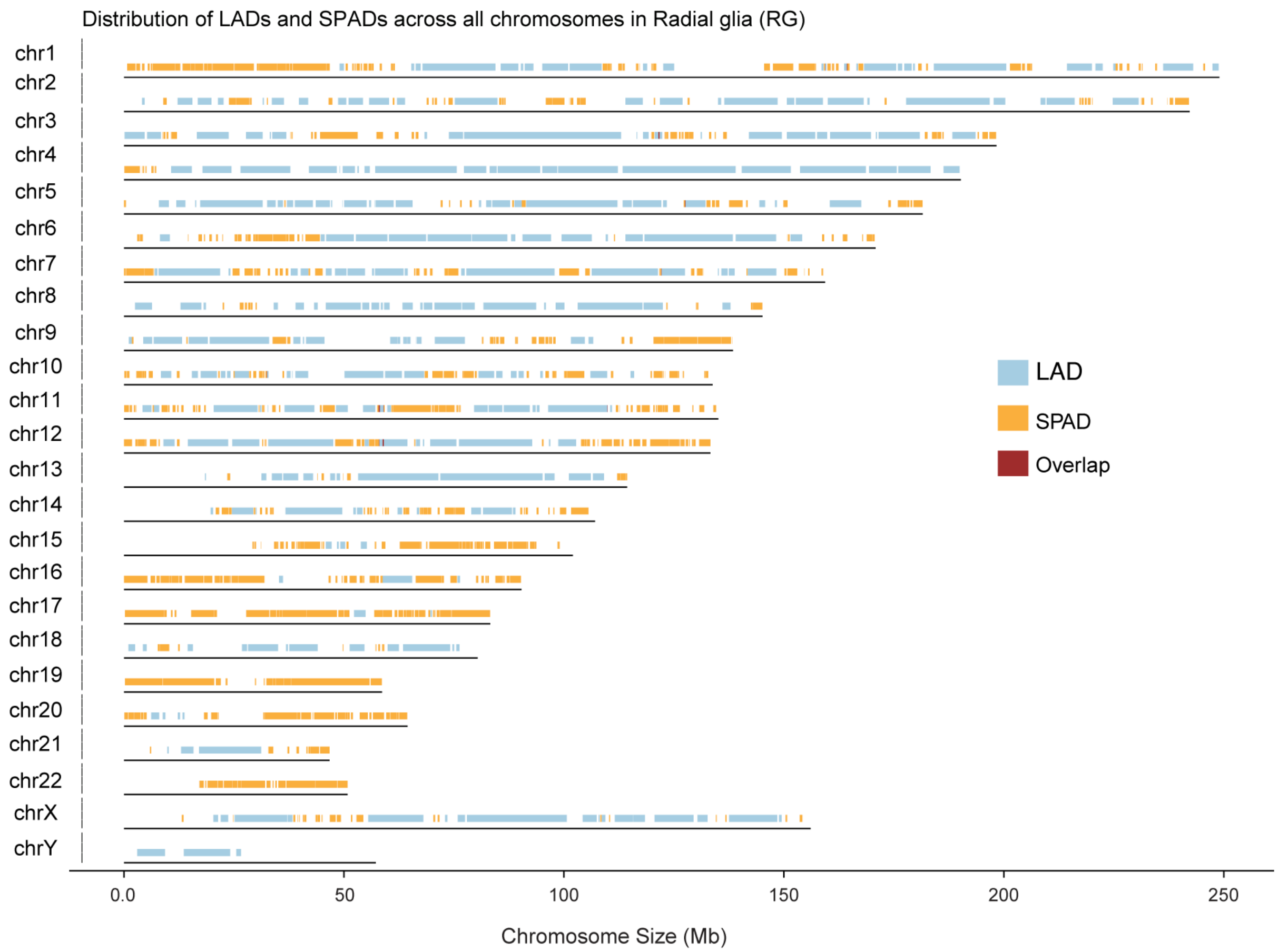

Reviewer Response Figure 8. Chromosome ideogram showing LADs and SPADs across all human chromosomes in GW17 RG.

3. The authors demonstrated that LAD-gain and LAD-loss are associated with the decrease and increase of gene expression during neurogenesis (RG to EN), respectively. However, the LAD-same regions also displayed significant gene expression changes between RG vs IPC and RG vs EN (Fig. 1D). The authors need to comment on this point.

In the revised Discussion, we have added the following comment to address this point: *"In this study, we focused primarily on LAD-loss, as this spatial movement of genomic regions*

corresponded to significant gene expression increase. While the average gene expression level in LAD-gain and LAD-same regions were also dynamic, these changes were more subtle in magnitude, suggesting that additional mechanisms, such as histone modifications, have strong interplay with lamina association."

If this reviewer is interested in a bit of speculation (not included in the revised text, at this point), we suspect that this phenomenon of LAD-same genes decreasing expression in IPC and EN reflects the progression to the terminally differentiated, postmitotic state of neurons. Since RG are multipotent stem cells, there are likely genes in LADs that are not expressed at a functional level as they are not involved in RG function, but may exist in a poised, lowly-expressed state (perhaps bivalency) in RG to be ready for expression upon differentiation to other fates (i.e., astrocytes and oligodendrocytes). When the RG/IPC undergoes irreversible differentiation into an EN, these alternative lineage genes in LADs may then become fully repressed (e.g., loss of H3K4me3), as they will never be "needed" again during the cell's lifetime.

4. When identifying differential LAD regions, the focus was only on RG and EN. What about IPC? Does it represent an intermediate state between these two?

As noted in our response to Reviewer Comment #1, our primary analysis of differential LAD regions focused on RG and EN because the LAD architecture of IPC was highly similar to that of RG (Jaccard index = 0.78-0.83, **Extended Data Fig. 2e**). This is consistent with their similar gene expression profiles, as both RG and IPC represent neural progenitor populations in the developing brain.

To assess whether IPCs represent a transitional state in LAD organization, we examined the LAD status of genomic regions that undergo remodeling during the RG-to-EN transition. Of the RG LADs that lost lamina association in EN (LAD-loss), 75% were also LADs in IPC. Likewise, of RG non-LADs that gained lamina association in EN (LAD-gain), 86% were also non-LAD in IPC, and 88% of LADs shared between RG and EN were also LADs in IPC. Notably, while the LAD architecture at RG-to-EN LAD-loss/gain regions was largely similar between RG and IPC (**reviewer Fig. 1b-c**), LaminB1 levels in IPCs were intermediate relative to RG and EN in LAD-loss regions (**reviewer Fig. 9, included in ED Fig. 2h**). By contrast, in LAD-gain regions, LaminB1 enrichment was equivalent in RG and IPC. These findings suggest that while the LAD architecture of IPCs more closely resembles that of RG than that of EN, IPCs do represent an intermediate or transitional state in the reorganization of LADs during neural differentiation.

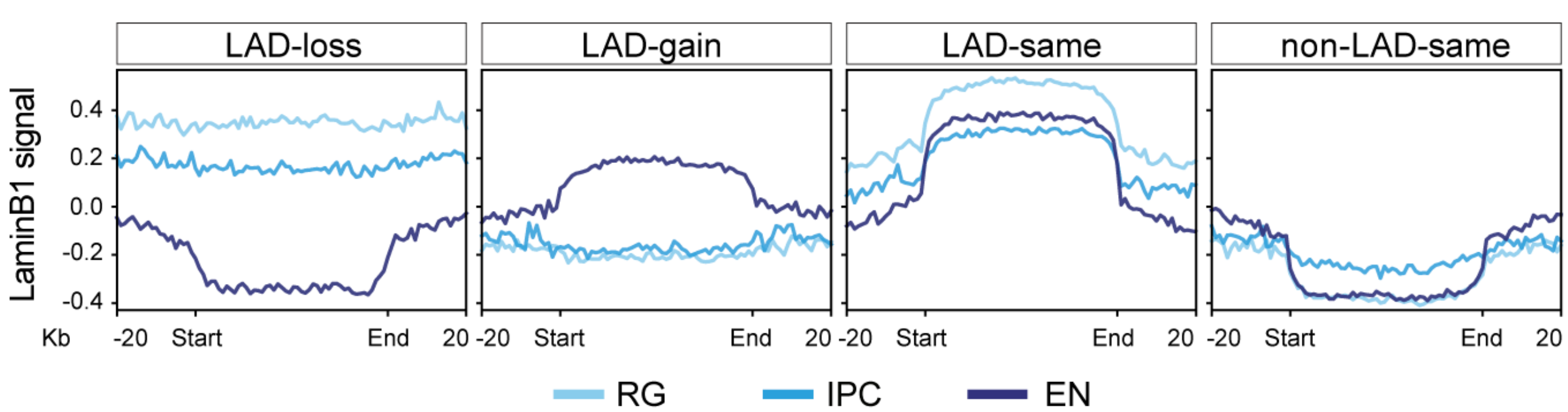

Reviewer Response Figure 9. Metagene plot depicting LaminB1 signal in LAD-loss, LAD-gain, LAD-same and non-LAD-same regions in RG, IPC and EN at GW17.

5. The authors need to provide characterization of the cell identity of the in vitro cultures which they used for profiling and validation studies.

In response to the reviewer's request, we have added detailed characterization of the in vitro cultures used in our profiling and validation studies. The identity of NPCs and NPC-derived neurons (iNs) was validated by immunostaining for NPC (PAX6, SOX2) and neuronal (Tuj1, NeuN) markers and RT-qPCR analysis of lineage-specific marker genes (**reviewer Fig. 10 a-b**). In addition, RNA-seq data corroborate these findings by demonstrating expression of appropriate NPC and neuronal markers (**reviewer Fig. 10c**). These data have been incorporated into the revised manuscript (**ED Fig. 8e-f**).

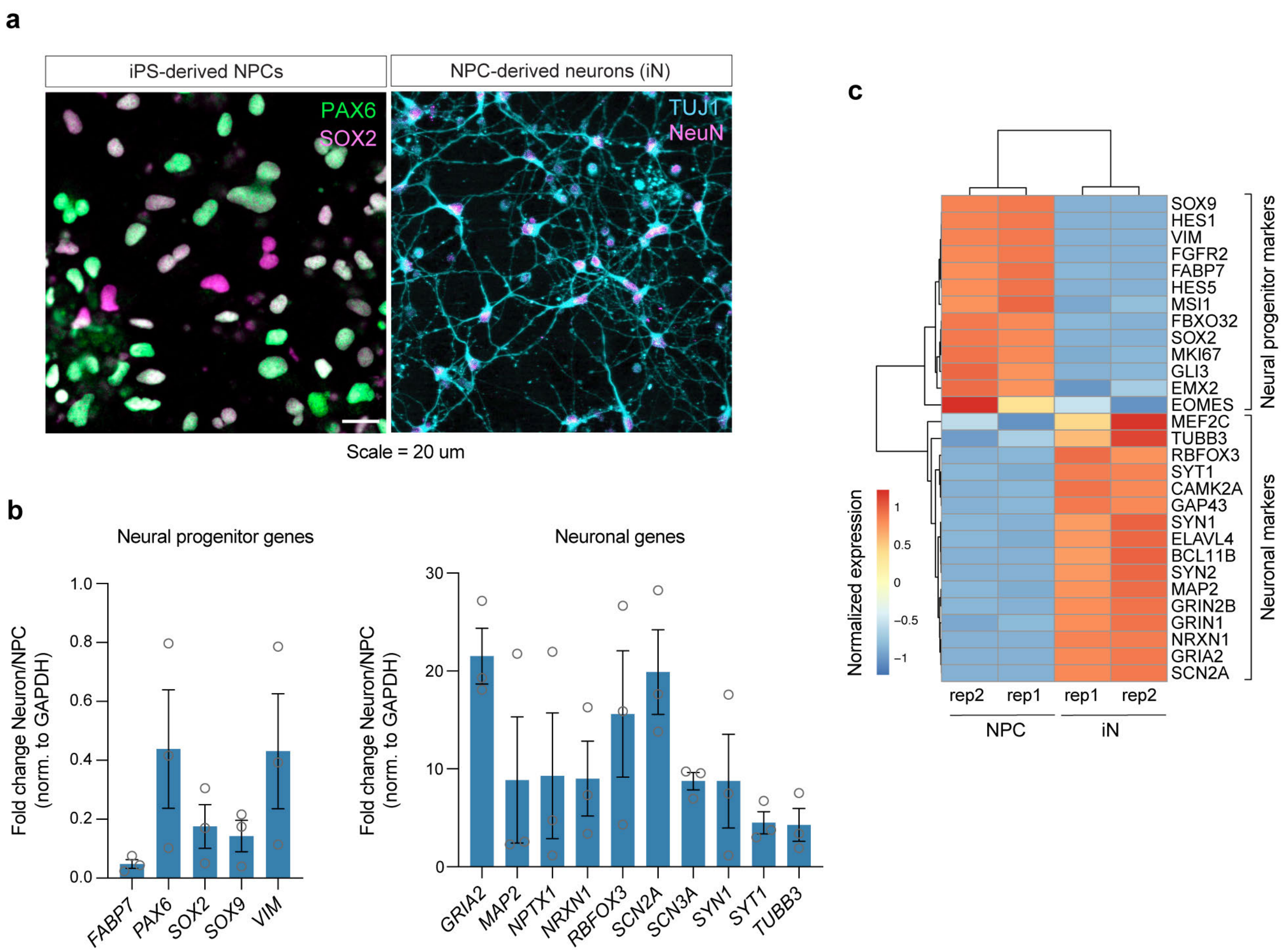

Reviewer Response Figure 10. a, Representative images of immunofluorescence staining for NPC and neuronal marks in iPSC-derived NPC and induced neurons (iN). **b,** RT-qPCR analysis of lineage-specific marker genes. **c,** Heatmap showing the expression (row normalized) of key NPC and neuronal marker genes in NPC and iN. Rep1 and rep2 indicate replicates.

Referee #2 (Remarks to the Author):

Ahanger et al performed genomic mapping of LADs and SPADs in midgestational human cortex in radial glia, intermediate progenitor cells and excitatory neurons. They find extensive reorganizations of genomic regions in both spatial compartments that include genes with neurodevelopmental function. Spatial repositioning away from the nuclear lamina and subsequent association with SPADs or non-LAD regions is associated with an increase in gene expression. The authors also identify genes in LADs that are in a bivalent state, for which rendering to monovalent H3K4me3 is associated with gene activation.

I find the observations interesting as not much work is performed in in vivo settings, though I also find that minimal new biological insight is obtained on the mechanistic role for LADs and SPADs in gene regulation. The manuscript is descriptive and data is at times difficult to interpret due to lack of analytical descriptions or in-debt qualitative and quantitative analyses. The concept that is proposed in the abstract is interesting, yet at present, I find it an oversimplification and insufficiently supported by the data. I find this manuscript more suited for a specialized genomics or neuronal journal and not for the broad readership of Nature.

We thank the reviewer for their thoughtful evaluation of our study and for recognizing the novelty of performing spatial genome mapping in primary human cortical cell types in vivo. We appreciate the reviewer's concerns regarding the depth of biological insight and the mechanistic clarity surrounding the role of LADs and SPADs in gene regulation. In response, we have substantially revised and expanded the manuscript to move beyond a descriptive framework and provide mechanistic evidence supporting the functional significance of spatial genome organization in neurodevelopment. We hope these additions address the reviewer's concerns, which have significantly strengthened our work.

To provide insight into the molecular mediators of lamina-associated transcriptional repression, we considered the possibility that lamina-associated transcriptional repression involves spatial segregation of active RNA Polymerase II (Pol II) and its regulators. We profiled the genome-wide occupancy of the initiating (Ser5P) and elongating (Ser2P) forms of Pol II during GW17 neurogenesis. RG LAD genes exhibited markedly lower levels of Pol II Ser5P and Ser2P as compared non-LAD genes (**ED Fig. 10f-g**).

Notably, bivalent genes in RG LADs showed reduced occupancy of elongating Pol II Ser2P relative to non-LAD bivalent genes, whereas Pol II Ser5P levels were similarly low at LAD and non-LAD bivalent gene promoters (**Fig. 6a**). Importantly, bivalent genes in LAD-loss regions, compared to bivalent genes genome-wide, showed greater increase in Pol II enrichment in EN, particularly for the elongating Ser2P form across the gene body (**Fig. 6b, ED. Fig. 10h-i**). These data suggest that proximity to the nuclear lamina restricts transcriptional elongation of bivalent genes.

To investigate whether the local environment of the nuclear lamina excludes transcriptional machinery necessary for productive transcription, we analyzed the subnuclear localization of Pol II regulatory cofactors involved in elongation. CDK9 and Cyclin T1 (positive transcription elongation factor P-TEFb subunits) – and ENL (super elongation complex), were depleted from the nuclear periphery and enriched in the interior (**Fig. 6c-d**). In contrast, the negative elongation factor NELF-B was relatively enriched at the lamina (**Fig. 6c-d**). Together, these findings support a model in which the nuclear lamina acts as a repressive environment by spatially segregating components essential for productive transcription. These new data have been incorporated into the revised manuscript in the subsection "Spatial segregation of Pol II

regulators limits transcription of bivalent genes at the lamina" and shown in **Fig. 6 and ED Fig. 10f-i**.

Below, we detail our responses to each of the reviewer's comments.

Comments in chronological order

-Line 167 reference to Fig. 1a should be Fig. 1b. This also makes that there is no reference to Fig. 1a.
This has been corrected in the revised manuscript.

-No LADs are found on five entire chromosomes. This is very surprising, unusual and unprecedented. This is a technical concern in relation to the sensitivity of the method. Perhaps due to the low-input cell number? This needs to be addressed i.e. by performing LAMINB1 GO-CaRT with dilution series of input material in comparable tissue types or iPCS. Or is this related to some form of computational thresholding that is applied? This I can't assess because of insufficient descriptions on this part (see comment below).

We have performed the suggested analyses and additional work to address these concerns about LAD coverage and other technical aspects of LaminB1 GO-CaRT. We describe the results of this work below:

LaminB1 GO-CaRT Sensitivity Validation:
As recommended by the reviewer, we performed a dilution series of LaminB1 GO-CaRT using freshly dissociated cells from the GW19 human germinal zone (RG-enriched) (**reviewer Fig. 11a**). We tested input levels of 5k, 10k, 25k, 100k, and 200k cells. As shown in the figure below, the resulting LaminB1 GO-CaRT tracks are highly consistent across all input levels (**reviewer Fig. 11b-c**). Importantly, even at the lowest cell numbers, LADs are still reliably detected, including on chromosomes typically exhibiting low lamina association (**reviewer Fig. 11c**). These results demonstrate that GO-CaRT is a highly sensitive and robust method for mapping LADs even with limited cell input.

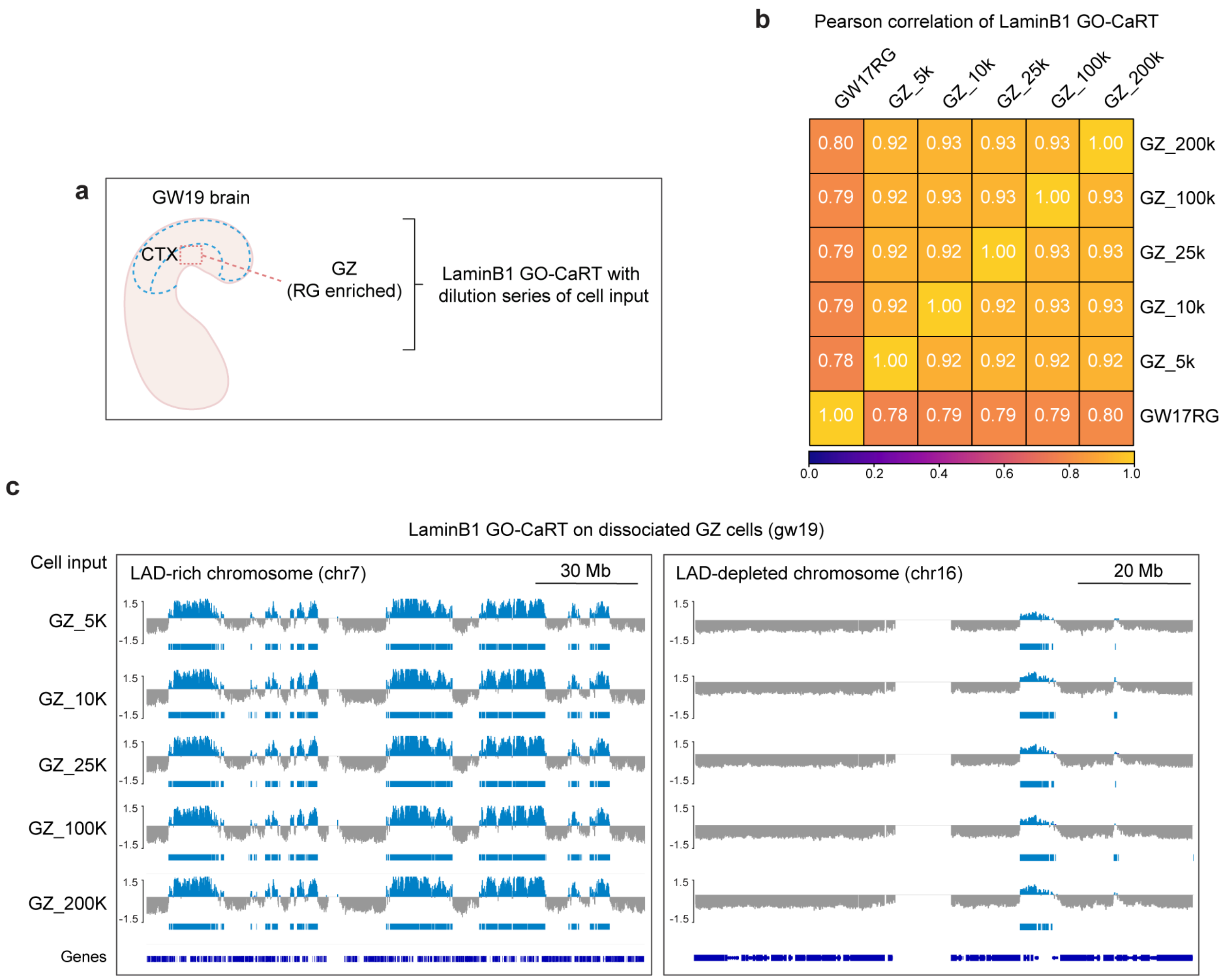

Reviewer Response Figure 11. a, Cartoon illustrating the microdissected germinal zone (RG-enriched) of GW19 cerebral cortex used for LaminB1 GO-CaRT with a dilution series of cell inputs. b, Spearman correlation of LaminB1 GO-CaRT profiles across different cell input amounts. c, Genome browser tracks of LaminB1 GO-CaRT for a LAD-rich chromosome (chr 7) and for a chromosome with low LAD coverage (chr 16).

Chromosomal LAD Coverage Clarification:
In the original Fig. 1b, we displayed only LADs that were observed at both GW17 and GW20. Due to compressed figure layout, LADs on smaller chromosomes – which we assume that the reviewer is referring to – may be difficult to visualize. We have now obtained LAD data from two age-matched replicates for both GW17 and GW20 and improved our LAD calling methods. Below we show genome browser tracks of LaminB1 GO-CaRT and LADs across these smaller chromosomes in RG, IPC and EN and previously published LAD profiles obtained with DamID in two cell lines (**reviewer Fig. 12**). These data show that LADs are detected across these smaller chromosomes, though we note that these smaller chromosomes exhibit very low LAD coverage, consistent with their more central positioning in the nucleus (e.g., Croft *et al*., J Cell Biol 1999; Bolzer *et al*., Plos Biol 2005; Guelen *et al*., Nature 2008). The revised Figs. 1-3 (previously Figs. 1-4) are now generated using the data from two biological replicates of GW17.

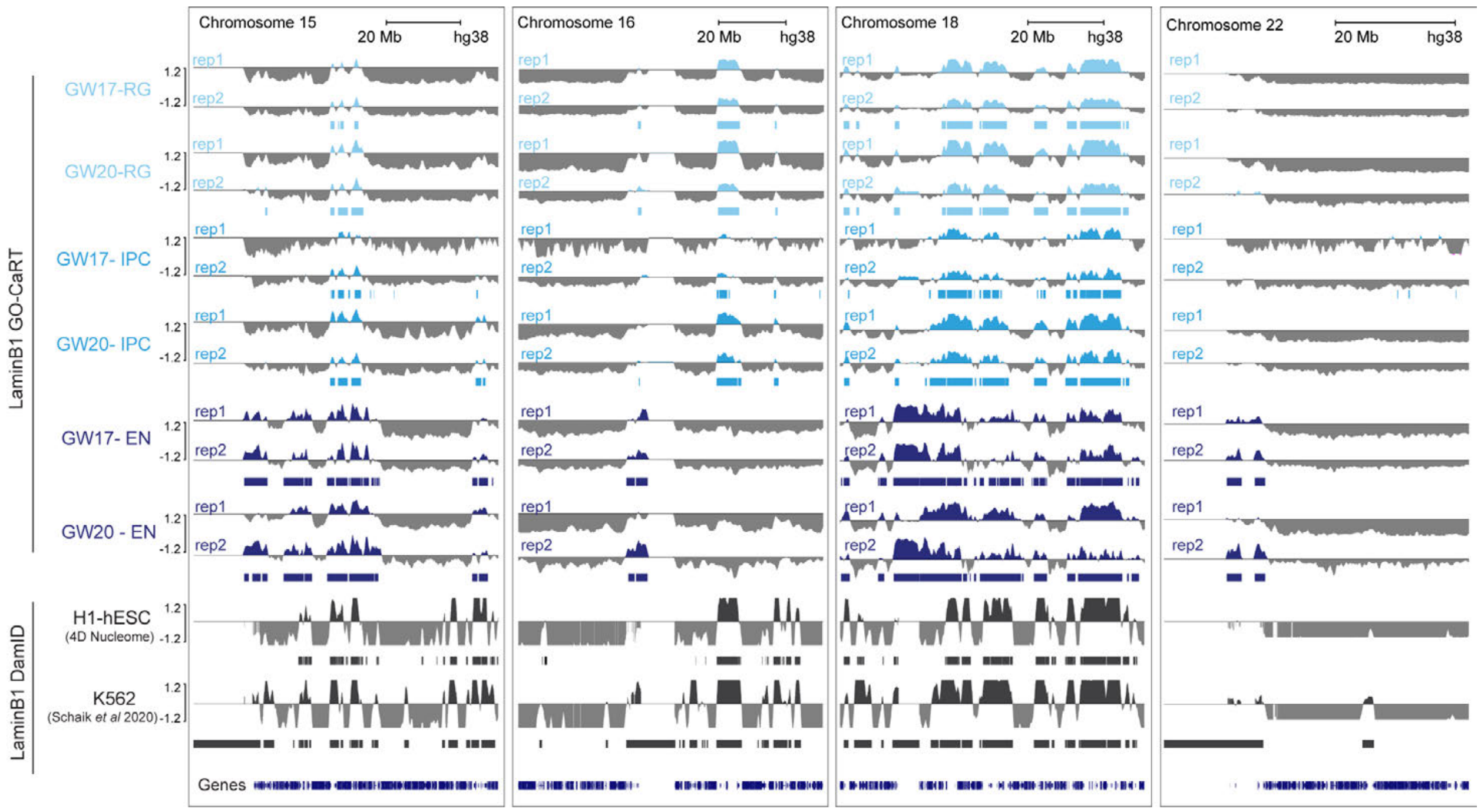

Reviewer Response Figure 12. Genome browser tracks of LaminB1 GO-CaRT from two biological replicates of RG, IPC, and EN isolated at GW17 and GW20. Shown are examples of chromosomes with low LAD coverage (chr15 chr16 and chr 22) and a LAD-rich chromosome (chr18). For reference, DamID tracks from H1-hESCs and K562 are included.

-Jaccard index (wrongly named Jaccard correlation) between GW17 and GW20 IPC is lower than between IPC and RG GW17 and GW20. This makes me question whether the origin of the cell-type specific changes are technical or biological.

We thank the reviewer for pointing out our mistaken use of the term “Jaccard correlation” in the manuscript. To more appropriately assess reproducibility, we now report Pearson correlation between replicates, as well as Jaccard index for LAD calls in the revised manuscript (**ED Fig. 2a, e**). Regarding the similarity in LAD architecture between IPC and RG, we note that both are neural progenitor populations in the developing human cortex and share overlapping transcriptional programs (Polioudakis *et al.*, Neuron 2019; Nowakowski *et al*., Science 2017). Therefore, the observed LAD similarity between IPC and RG, relative to EN (**ED Fig. 2e-f**), is consistent with their developmental and molecular relatedness.

We also acknowledge that comparing GW17 and GW20 samples is not optimal for assessing technical reproducibility due to potential developmental differences. To directly address this, we generated LaminB1 GO-CaRT profiles from RG, IPC, and EN cells isolated from two independent, age-matched GW17 biological replicates (**ED Fig. 2a**). These new data demonstrate high Pearson correlations between replicates for each cell type, reinforcing both the technical reproducibility and biological specificity of our LAD profiling approach (also see response to the next comment below).

-What is the reason behind choosing GW17 and GW20 as replicates? I assume this is because of tissue availability, but there is no explanation provided and these samples do not represent true biological replicates. This decision complicates data interpretation.

We agree that GW17 and GW20 represent distinct developmental time points and thus are not 'true' biological replicates. Initially, these samples were analyzed together due to practical constraints of limited tissue availability. However, we recognize that this approach may complicate data interpretation.

To directly address these concerns, we profiled LADs from RG, IPC, and EN populations in additional GW17 tissue specimens, thereby obtaining true biological replicates at this developmental stage. LAD profiles from GW17 RG, IPC and EN showed strong concordance between replicates (Pearson correlation 0.72-0.94; **reviewer Fig. 1a**). Moreover, LAD profiles were largely similar between RG and IPC according to Jaccard index calculations for LAD profiles called on the median of the two biological replicates (**response Fig. 1b**) and PCA analysis of LaminB1 GO-CaRT (**response Fig. 1c**). Thus, for analyses of LAD dynamics during neurogenesis, we focused on comparisons between RG and EN. SPAD profiles for RG, IPC and EN were also generated from two age-matched (GW17) biological replicates. For downstream analyses, SPAD calls from two replicates were intersected.

Importantly, our main conclusions remain unchanged. Analyses based solely on GW17 biological replicates confirm the extensive remodeling of LADs and SPADs during the RG to EN differentiation, as well as the strong association between subnuclear genome compartmentalization and the regulation of bivalent chromatin state (**revised manuscript Figs. 1–3**). Further, analysis of two biological replicates each at GW17 and GW20 revealed that the LAD architectural changes observed at GW17 are essentially the same at this later developmental stage (**ED Fig. 2a-g; ED Fig. 3; ED Fig. 4c**), supporting the conclusion that LAD remodeling is a robust feature of human neurogenesis.

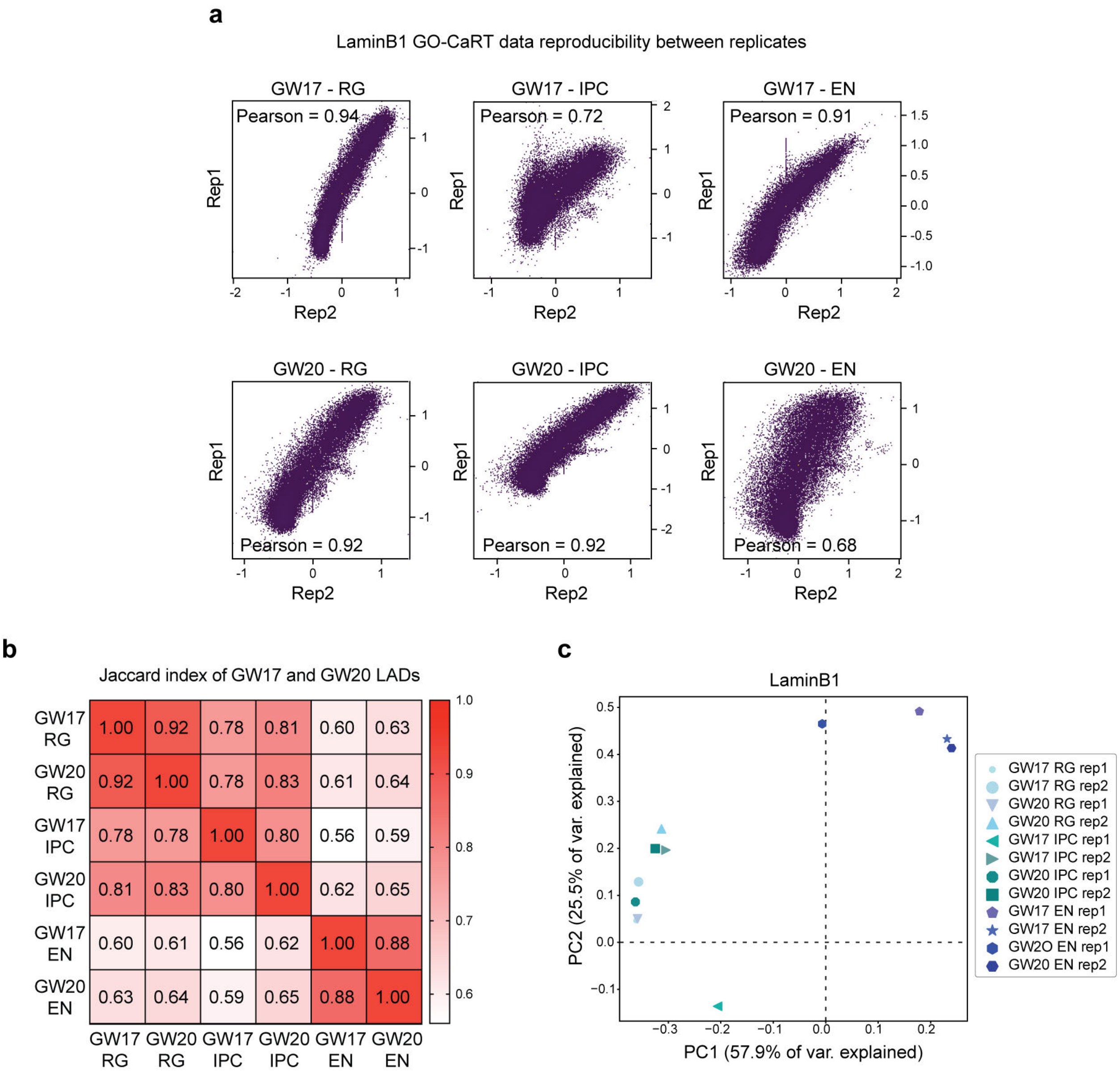

Reviewer Response Figure 1. a, Pearson correlation of LaminB1 GO-CaRT profiles of RG, IPC and EN isolated by FANS from GW17 and GW20 brain cortex. **b,** Jaccard similarity index of LADs in RG, IPC and EN at GW17 and GW20. **c,** PCA plot of LaminB1 GO-CaRT profiles of RG, IPC and EN at GW17 and GW20.

-Quantitative comparisons are performed between RG and EN stages for Fig ED3e-f. However, there is no mentioning of how this comparison is performed. I assume this is based on the combined data of the GW17 and GW20 sample of each of the three cell types? Also, how is the comparison performed? Is this based on domain calling or some binary cut-off? Smoothing or not? How is the data normalized and is the data quality between the three cell types compatible? All these factors are hugely important for data interpretation and evaluation and much more in debt descriptions (both in main and methods) and analyses are needed to substantiate and evaluate these findings.

We apologize for the lack of detail in the original submission regarding the data quality assessment and LAD remodeling analysis presented in Fig. ED3e–f. We agree that data quality assessment and analytical procedures are critical for accurate interpretation of LAD remodeling and have now addressed these points thoroughly in the revision.

We performed multiple quality control assessments and show that data quality is comparable across cell types. Specifically, human genome alignment rate, fragment size distribution, duplication rate, and sequencing depth post-duplicate removal were all similar across cell types (**reviewer Fig. 13**). To ensure accurate comparisons between experimental samples, we performed normalizations of sample reads by downsampling them to be the same for LaminB1 (or SON) antibody and IgG within each sample.

In the original submission, LAD remodeling analyses were performed using domain calling data from RG and EN at both GW17 and GW20. In the revised manuscript, we now focus our analysis on age-matched biological replicates of RG and EN at GW17 (and a separate analysis at GW20) to ensure a more controlled and interpretable comparison. Importantly, our main conclusions remain unchanged with this revised approach. We have now included detailed descriptions of the analysis pipeline in Methods sections of the revised manuscript, including how domains were called, the criteria for LAD identification, normalization procedures, and quality control metrics across cell types.

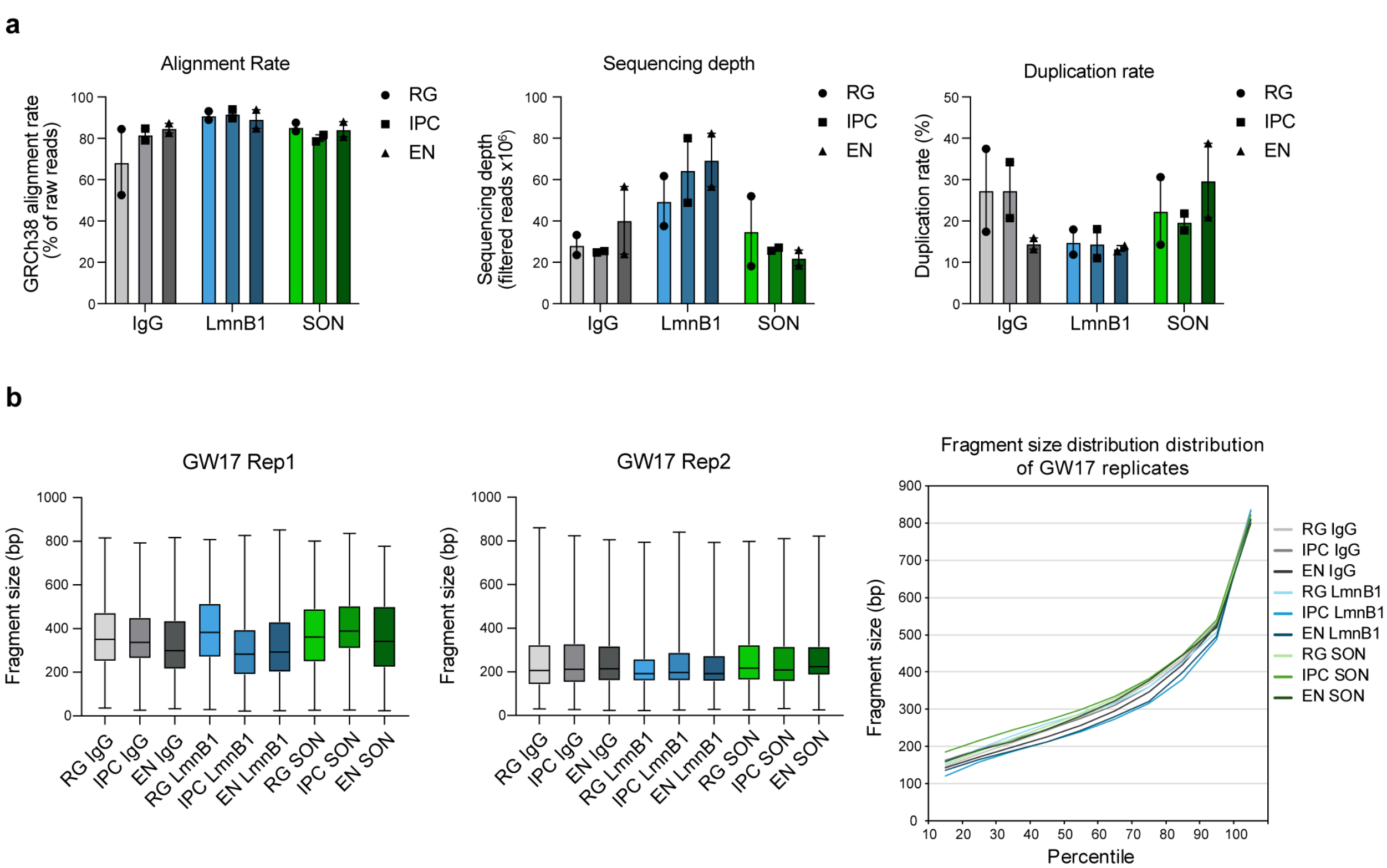

Reviewer Response Figure 13. Data quality analyses showing Alignment rate, sequencing depth, and duplication rate for LaminB1 GO-CaRT, SON GO-CaRT and IgG controls across RG, IPC and EN at GW17. **b,** Fragment size distribution for LaminB1 GO-CaRT, SON GO-CaRT and IgG controls across RG, IPC and EN at GW17.

-Fig. ED1d it is unclear if i.e. LAD loss in EN represents the change compared to IPC -> RG or both. It would be informative to include the number of genes involves per category.

We believe that the reviewer is referring to Fig. 1d. We now clarify in the text that LAD-loss/gain in EN represents the change in comparison to RG, as the LAD architecture between RG and IPC is largely similar (**reviewer Fig. 1b-c**). We also now include the number of genes in each LAD remodeling category in the figure legends.

Also, the effect size is very small between most classes. With genomics data the tiniest changes are often significant, yet that does not necessarily mean that the effect is relevant. I.e. in the LAD-same category, the effect size of the changes are within similar range between all categories except for the increase in mRNA counts for the LAD-gain EN category. I therefore also find the statement on lines 180-182 not nuanced enough. I would say that the change in mRNA counts is neglectable for the LAD-gain category.

We agree that the observed changes in gene expression for the LAD-gain and LAD-same regions are subtle. We have modified the text in the Results and Discussion accordingly and now display **Fig. 1d and ED Fig. 4c** as a violin plot so readers can more accurately assess the distribution of the data. In the revised Discussion, we have added the following comment: *"In this study, we focused primarily on LAD-loss, as this spatial movement of genomic regions corresponded to significant gene expression increase. While the average gene expression level in LAD-gain and LAD-same regions were also dynamic, these changes were more subtle in magnitude."*

-It is unclear whether the SON-Go-CaRT data is obtained with GW17 and GW20 stages like for LADs. I am missing a comparison between the replicates to assess overall data quality. I.e. the Jaccard index for Fig. ED4b does not include replicates. Unlike the LAD data in Fig. 1b, the SON data in Fig. 2b appears fragmented and much noisier. An in-debt analysis of data quality is needed to evaluate the profiles. Similarly, the dynamic range of the data plotted in Fig. 2d is very narrow. This usually is an indicator of poor data quality. The y-axis label does not indicate what is plotted here, so I find it hard to assess what the nature of the low dynamic range could be (plus my comments above).

We apologize for the lack of clarity regarding the SON GO-CaRT dataset in the original submission. The SON GO-CaRT data presented in the original submission were generated from GW17 samples. In the revised manuscript, we have added a second biological replicate from age-matched GW17 tissue for SON GO-CaRT profiling across RG, IPC, and EN populations to strengthen the robustness of our findings. Pearson correlation between SON GO-CaRT replicates were 0.62 for RG, 0.61 for IPC and 0.57 for EN (**reviewer Fig. 14a**), and RG and IPC profiles were more similar to each other than to EN (**reviewer Fig. 14b, included in the revised manuscript, ED. Fig. 5)**. We agree that the SON GO-CaRT profiles appear noisier than the LAD data. This observation is consistent with the lower signal-to-noise ratio we have previously reported for SON profiling in primary tissues compared to cell lines (Ahanger *et al.* 2021 Nat. Neurosci.). To increase rigor of our analyses, we called SPADs on each replicate individually and used the intersection of the two replicates' SPAD calls for further analysis.

Regarding Fig. 2d, we have clarified that the y-axis represents the log_2(SON/IgG) enrichment. The dynamic range for SON-GO-CaRT is indeed narrower compared to LaminB1 GO-CaRT, which likely reflects both biological differences in chromatin associations and the inherently lower signal-to-noise ratio of SON profiling in primary tissue.

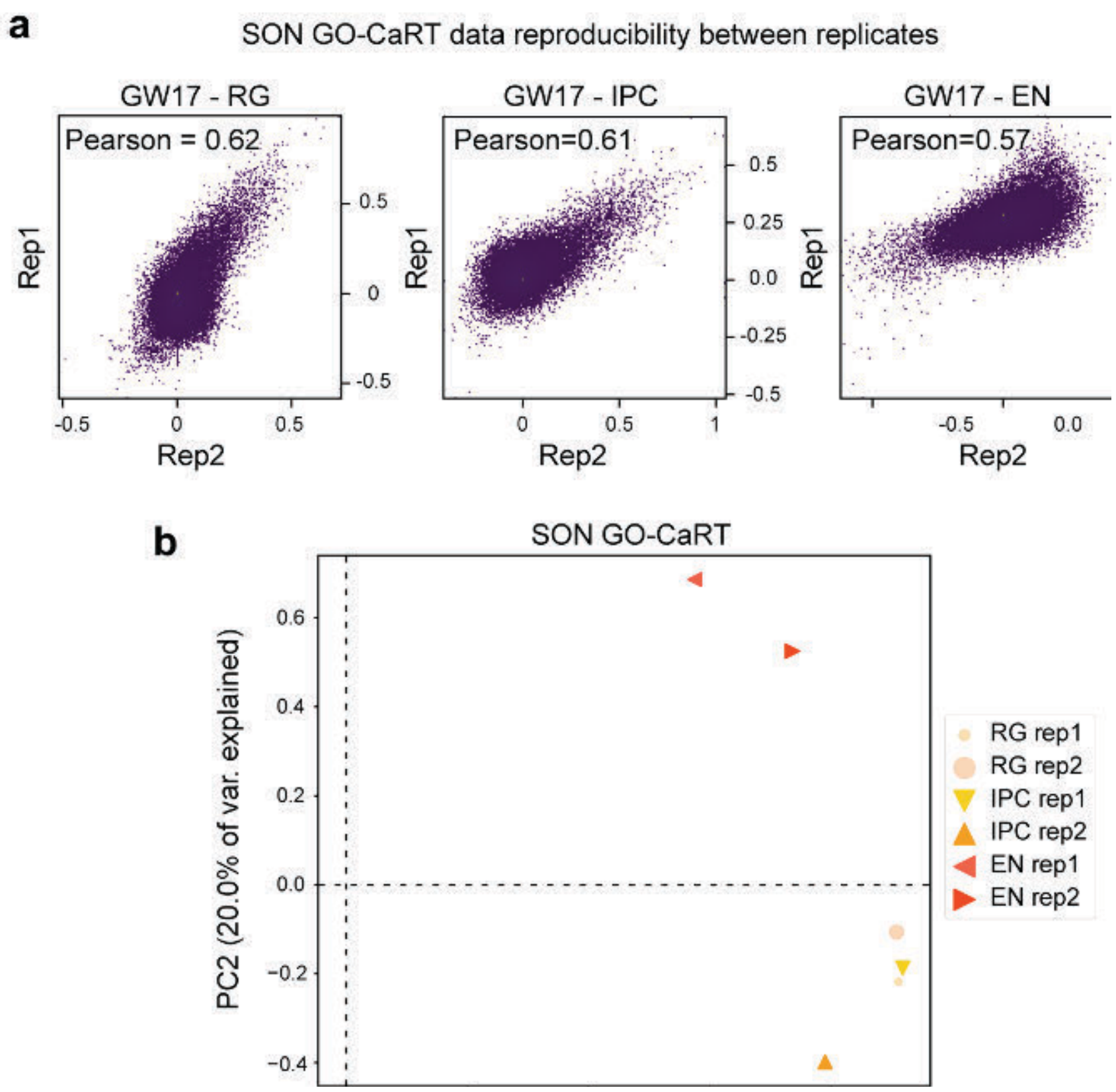

Reviewer Response Figure 14. Replicate reproducibility of SON GO-CaRT. a, Pearson correlation of SON GO-CaRT profiles of GW17 RG, IPC and EN b, PCA plot of SON GO-CaRT profiles of RG, IPC and EN at GW17.

-Fig. ED4a I am surprised genes in LADs are so short. Normally genes in LADs are long, such as the olfactory receptor genes.

We thank the reviewer for pointing this out. In the analysis of the new LAD data, we confirm that genes in LADs are indeed longer as compared to genes in SPADs (**reviewer Fig. 15a; ED Fig. 6c**). Importantly, this re-analysis does not affect our previous findings regarding greater isoform diversity and higher expression levels (**reviewer Fig. 15b, c; Fig. 2b; ED Fig. 6c**) for SPAD-associated genes.

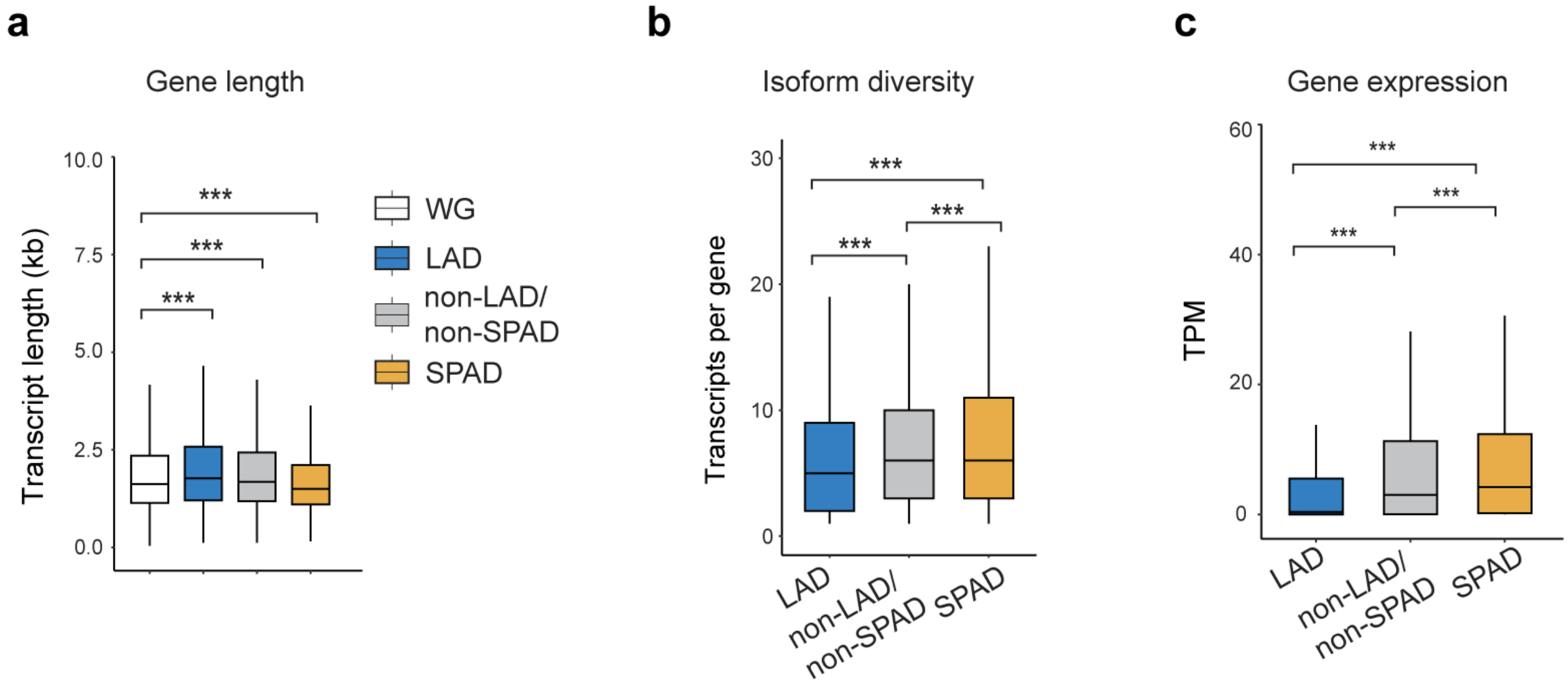

Reviewer Response Figure 15. a, Average transcript length, **b,** isoform diversity and **c,** gene expression in different genomic compartments across the neurogenic lineage at GW17.

-What is the genomic coverage of SPADs per cell type? It appears EN cells have much lower coverage? Is this related to data quality? In general, in such instances, the domain-size is also smaller as domains become more fragmented due to detection limitations. This needs to be shown and addressed. Especially when concluding that the SPADs in EN are very different from IPC and RG.

We acknowledge that differences in SPAD coverage by cell type could potentially be due to data quality. There is indeed the least SPAD coverage in EN, with SPADs encompassing 643 Mb in RG, 581 Mb in IPC, and 380 Mb in EN. However, various quality control metrics (alignment rate, duplication rate, read depth, and fragment size) were not significantly different for SON profiles between cell types (**reviewer Fig. 13**). While previously optimizing GO-CaRT, we found that read depth can be as low as 10 million reads without compromising data quality for nuclear compartments. Notably, all SON samples maintained a depth of at least 20 million reads after duplicate removal and filtering of low-quality reads (MAPQ score <30). So, while we therefore feel it is unlikely that data quality or detection limits have substantially impacted our conclusions, we do agree with the reviewer that SON genome tracks appear more fragmented than those for LaminB1 (see comment above). To account for potential variability and increase rigor of our analyses, we called SPADs on each replicate individually and used the intersection of the two replicates' SPAD calls for further analysis. We agree with the reviewer that this may represent a methodological limitation and have therefore removed discussion of differences in SPAD architecture between EN and RG/IPC from the revised manuscript.

-For the GO-analyses in Fig.3 it would be good to include only the genes that change in expression upon LAD/SPAD loss or gain between cell types. Many genes with LADs are "bystander" products of spatial genome repositioning, yet are irrelevant to the biological process.

Following this suggestion, we performed GO analyses in **Fig. 2** (previously Fig. 3) using only genes that change in expression during RG to EN differentiation. Using this criterion, LAD-loss and LAD-to-SPAD regions were still enriched for GO terms related to neurodevelopment, neurogenesis and synapse organization (**reviewer Fig. 16**). LAD-gain regions were not enriched for any specific cellular pathway.

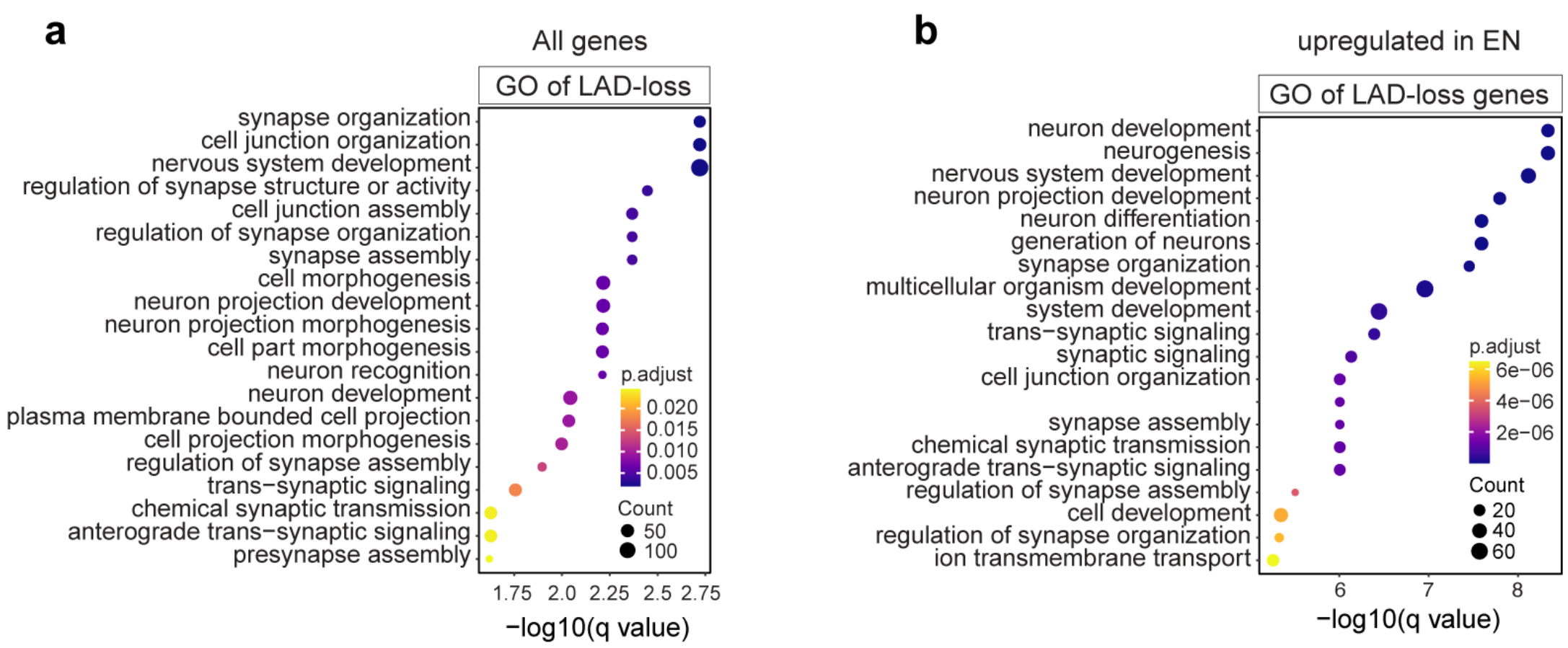

Reviewer Response Figure 16. a, GO analyses of all genes in LAD-loss regions. **b,** GO analyses of genes in LAD-loss regions that are upregulated in EN.

-The conclusion that bivalent genes in LADs are enriched for neurodevelopmental function on lines 270-271 requires a comparison to the entire genome and non-LADs. It is expected that across the genome in RG cells genes with bivalent marks are enriched for neurodevelopmental. This does not necessarily mean anything in relation to LAD function.

As the reviewer suggested, we performed GO analyses of bivalent genes in LADs and non-LADs using the whole genome as the background. These analyses revealed that both LAD-and non-LAD-associated bivalent genes were enriched for GO terms related to neurodevelopmental functions, though non-LAD bivalent genes were overrepresented in GO terms related to synapse function (**reviewer Fig. 17**).

However, our data demonstrate that both the resolution and transcriptional fate of bivalent genes is powerfully shaped by LAD dynamics. During RG-to-EN differentiation, only ~39% of non-LAD bivalent genes resolve to H3K4me3 with a modest ~2-fold activation (similar to the whole-genome average, **reviewer Fig. 17c, d**). In contrast, bivalent genes released from LADs exhibit much higher resolution of bivalency and strong transcriptional upregulation (**reviewer Fig. 17c, d)**. In the subset of LAD-loss regions that relocated to non-LAD/non-SPADs, ~65% of RG bivalent genes became monovalent for H3K4me3 and exhibited a ~3-fold increase in gene expression in EN. In the subset of LAD-loss regions that relocated to SPADs in EN, ~85% of RG bivalent genes were resolved to the H3K4me3--monovalent state with an ~8-fold increase in expression – significantly higher than all other spatial categories. These results suggest that while bivalent genes across the genome are enriched for neurodevelopmental functions, LAD-loss-associated bivalent genes undergo more pronounced resolution and transcriptional activation during RG-to-EN differentiation, highlighting LAD dynamics as a key contributor to neurogenic transcription. These data are included in in the revised manuscript, **Fig. 3** and **ED Fig. 7c-f**.

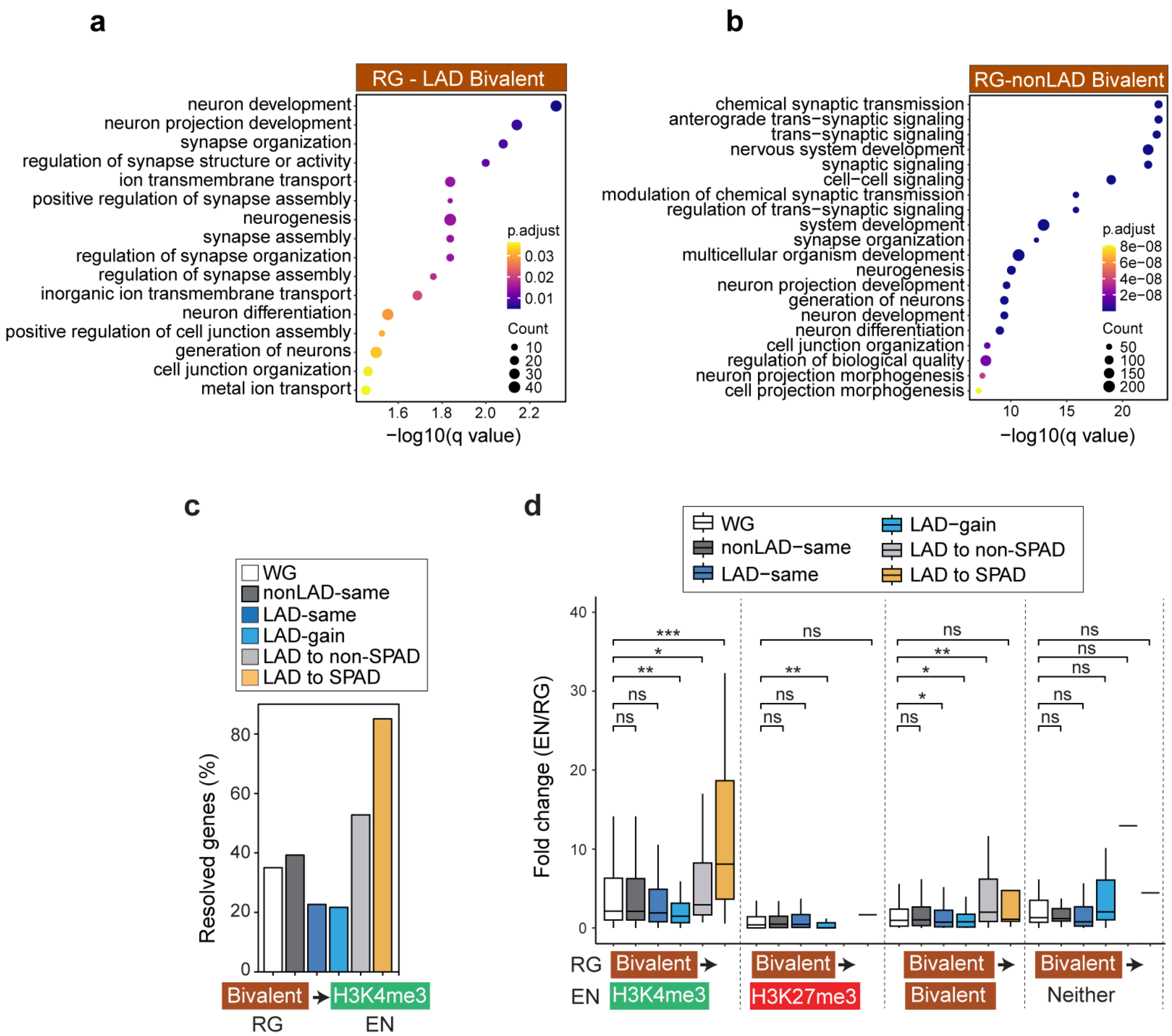

Reviewer Response Figure 17. a,b, GO analyses of RG LAD and non-LAD bivalent genes. **c,** Percentage of RG bivalent genes that resolve to the H3K4me3-monovalent state in EN stratified by LAD-to-SPAD dynamics. **d,** Fold change of expression of bivalent genes that resolve to the H3K4me3-monovalent, H3K27me3-monovalent, remain bivalent, or lose both marks during RG to EN differentiation stratified by LAD to SPAD dynamics.

-For data interpretation, I suggest to plot the levels of H3K27me3 and H3K4me3 over promoters for the bivalent category stratified by LADs, non-LADs and SPADs. Are the levels of these marks the same? If not, this may also explain why these genes are transcribed at lower levels compared to non-LAD regions. LADs are known to attenuate gene activities in general.

We agree with the reviewer that differences in H3K4/27me3 levels between compartments could confound the interpretation of gene expression differences. We performed the requested analysis (**reviewer Fig. 18**) and found that H3K4me3 levels at bivalent gene promoters were identical across compartments in RG. Similarly, H3K27me3 levels were equivalent between all bivalent genes and those in LADs and were actually lower at LAD bivalent genes than at nonLAD/nonSPAD bivalent genes, suggesting that differences in promoter enrichment for H3K4/27me3 do not explain the low expression of lamina-associated bivalent genes.

H3K4me3 and H3K27me3 levels at bivalent gene promoters stratified by genomic compartment

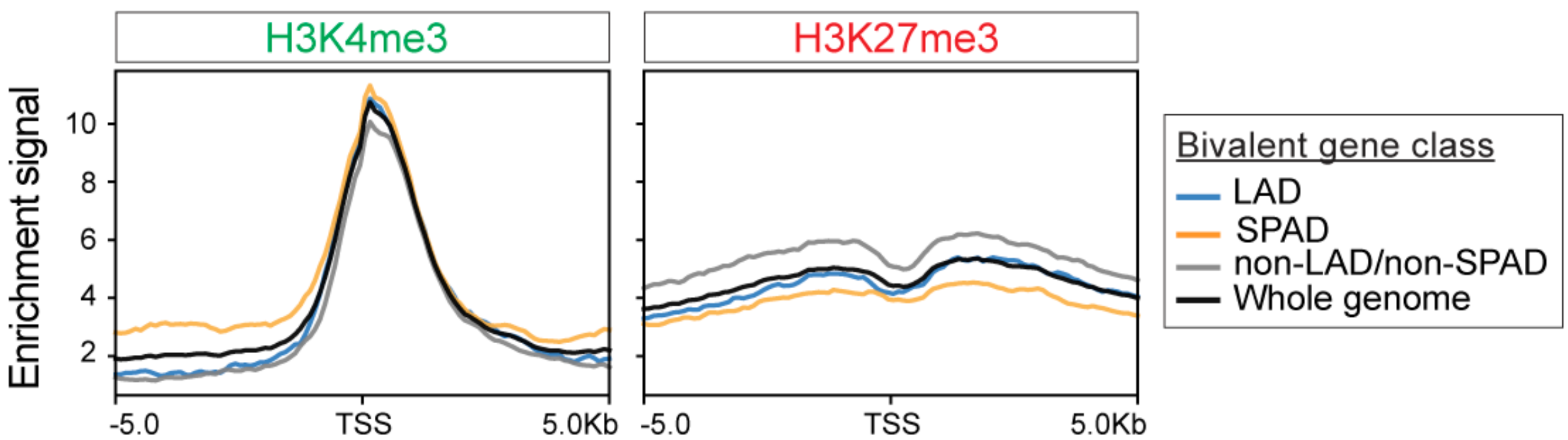

Reviewer Response Figure 18. Metagene plots of H3K4me3 and H3K27me3 signal, centered on the TSS of bivalent genes in different genomic compartments.

-Fig. 4c the gene expression changes of genes going from bivalent (RG) to monovalent H3K4me3 (EN) is similar for genes that stay within LADs (LAD-same) as for genes that change their spatial location to SPADs or elsewhere. What does that mean for the importance of spatial redistribution of genes for gene regulation? And the overall conclusions related to bivalency in general?

In Fig. 4c of the original manuscript, we observed that bivalent genes in RG that resolved to monovalent H3K4me3 in EN showed increased expression, but this was not significant for LAD-same regions. In contrast, bivalent genes that resolved to monovalent H3K4me3 and moved from LADs to SPADs exhibited a ~7-fold increase in expression.

In the revised manuscript (and **reviewer Fig. 17 above)**, we reanalyzed this using histone marks, LAD and SPAD data from age-matched GW17 replicates. Across the genome, ~35% of RG bivalent genes resolved to H3K4me3 monovalency in EN; these genes exhibited a 2.1-fold average increase in expression (**revised Fig. 3b, c**). In regions located outside of LADs in both RG and EN, expression increases were similar (2.1-fold) to the whole-genome average (**revised Fig. 3b, c**). In LAD-same and LAD-gain regions, gene expression increases were 1.9-fold and 1.4-fold, respectively, with resolved genes in LAD-gain regions being significantly less induced than the whole-genome average (**revised Fig. 3b, c**). Likewise, resolution frequencies were lower (around 20% for both LAD-same and LAD-gain) than the whole-genome average.

Consistent with our previous observations, transcriptional upregulation was significantly higher in LAD-loss regions. In the subset of LAD-loss regions that relocated to non-LAD/non-SPADs, ~65% of RG bivalent genes became monovalent for H3K4me3 and exhibited a ~3-fold increase in gene expression in EN (**revised Fig. 3b, c)**. In the subset of LAD-loss regions that relocated to SPADs in EN, ~85% of RG bivalent genes were resolved to the H3K4me3--monovalent state with an ~8-fold increase in expression – significantly higher than all other spatial categories (**revised Fig. 3b, c**). These findings indicate that spatial repositioning away from the lamina, particularly toward speckles, strongly promotes resolution of bivalency and robust transcriptional activation during RG-to-EN differentiation.

-It would be interesting to plot the re-ChIP experiments in Fig.5 genome-wide. I.e. as a density scatter plot of all bi-valent genes in LADs (H3K4me3 and H3K27me3 on the axis) compared to non-LADs or SPADs.

We would like to clarify our LaminB1 GO-CaRT-ChIP-reChIP methodology shown in the original Fig. 5 (**Fig. 4 in the revised manuscript**). This experiment was designed to confirm the bivalent chromatin state of LAD-associated genes. To do so, we first performed LaminB1 GO-CaRT on NPCs. We then carried out sequential chromatin immunoprecipitation (ChIP-reChIP) using LaminB1 GO-CaRT–released chromatin as input, with H3K27me3 ChIP followed by H3K4me3 ChIP, and vice versa (referred to as LaminB1 GO-CaRT–ChIP-reChIP). Thus, only LAD genes (not non-LAD or SPAD genes) can be analyzed in this dataset.

As suggested, we have now extended this analysis by plotting the LaminB1 GO-CaRT.ChIP-reChIP data genome-wide for H3K4me3-monovalent, H3K27me3-monovalent, and bivalent LAD genes identified independently by CUT&RUN for H3K4me3 and H3K27me3 (**reviewer Fig. 19**). These results are now included in the revised manuscript (**ED Fig. 8d**).

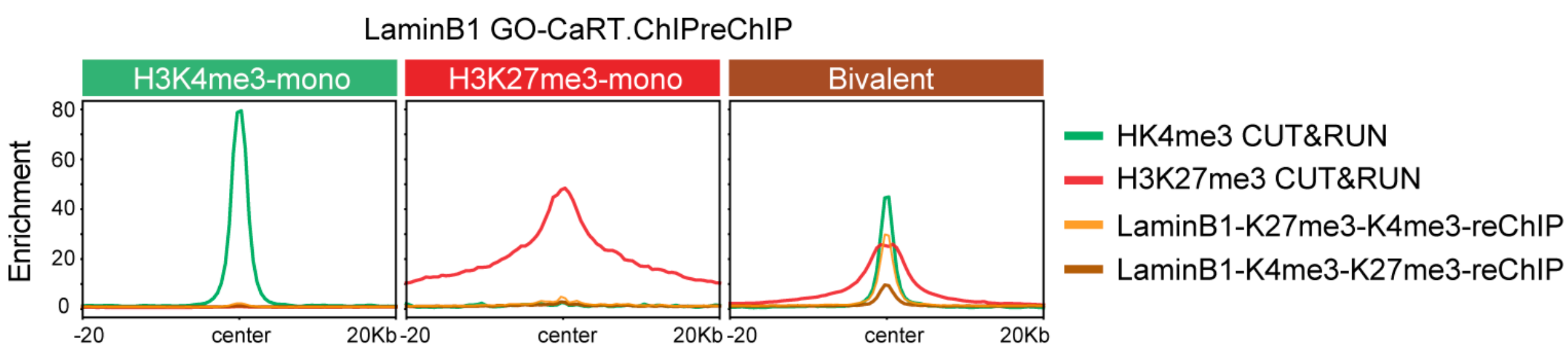

Reviewer Response Figure 19. Metagene plots of LaminB1 GO-CaRT-ChIP-reChIP signal at H3K4me3-monovalent, H3K27me3-monovalent, and bivalent genes as defined by independent CUT&RUN profiling for H3K4me3 and H3K27me3.

-In Fig. 6d the data is noisy for LAMINB1 and H3K27me3 and the difference between NPC and iN is very small. Is this related to few genes included in this analysis? I am surprised that genes are identified as LAD-loss if the differences are so small. This may be related to the flanking regions being more LAD-associated in NPC compared to iN, yet the promoters do not. Because of the domain calling, these promoters then become annotated as LAD regions. This is also apparent from the examples in Fig. 4d-e. It appears the promoters of the bivalent genes are not actually themselves associated with the nuclear lamina. How do the authors interpret this, and can we then still talk about spatial repositioning?

In the case of H3K27me3, the reviewer's keen observation caused us to realize that we mistakenly plotted log2-transformed (IgG-normalized) H3K27me3 tracks for this figure (apparent because the y-axis goes into negative values), which was inconsistent with our presentation of histone data throughout the rest of the manuscript. The variation introduced by IgG reads creates a noisy/jagged appearance and reduces the apparent magnitude of difference between the NPC and iN conditions. We have therefore re-plotted the RPGC-normalized H3K27me3 signal in the revised manuscript (**ED Fig. 9b, d**) to be consistent with our data presentation in the other figures.

We agree with the reviewer's observation that the LaminB1 signal in Fig. 6d appears noisy, which is indeed due to a limited number of genes in this analysis. Indeed, when we plot LaminB1 enrichment across LAD-loss/gain regions, the contrast in LaminB1 enrichment between NPC and iN becomes is much more apparent (**reviewer Fig. 20a,** also included in revised manuscript **ED Fig. 9a**). These differences in LaminB1 signal are also evident for bivalent genes in LAD-loss and LAD-gain regions (**reviewer Fig. 20b).**

To address the reviewer's point regarding local depletion of LaminB1 levels around bivalent promoters, we quantified LaminB1 signal within 300 kb of the transcription start site (TSS) for LAD-associated genes, stratified by chromatin state (**reviewer Fig. 20c**). This analysis showed that the reduced LaminB1 signal near bivalent promoters is not unique to this group, but represents a broader pattern seen across all LAD-associated genes, regardless of chromatin state. As a control, genes located in non-LAD regions showed no LaminB1 enrichment, as expected. Importantly, the degree of this depletion varies by chromatin context: genes marked by H3K4me3 exhibit the most pronounced reduction in LaminB1 signal, followed by bivalent genes and H3K27me3-monovalent genes. These findings suggest that local variation in lamina association – reflected by LaminB1 enrichment – alongside promoter chromatin state, may modulate transcriptional activity within LADs, and help explain why some H3K4me3-marked LAD genes remain highly expressed.

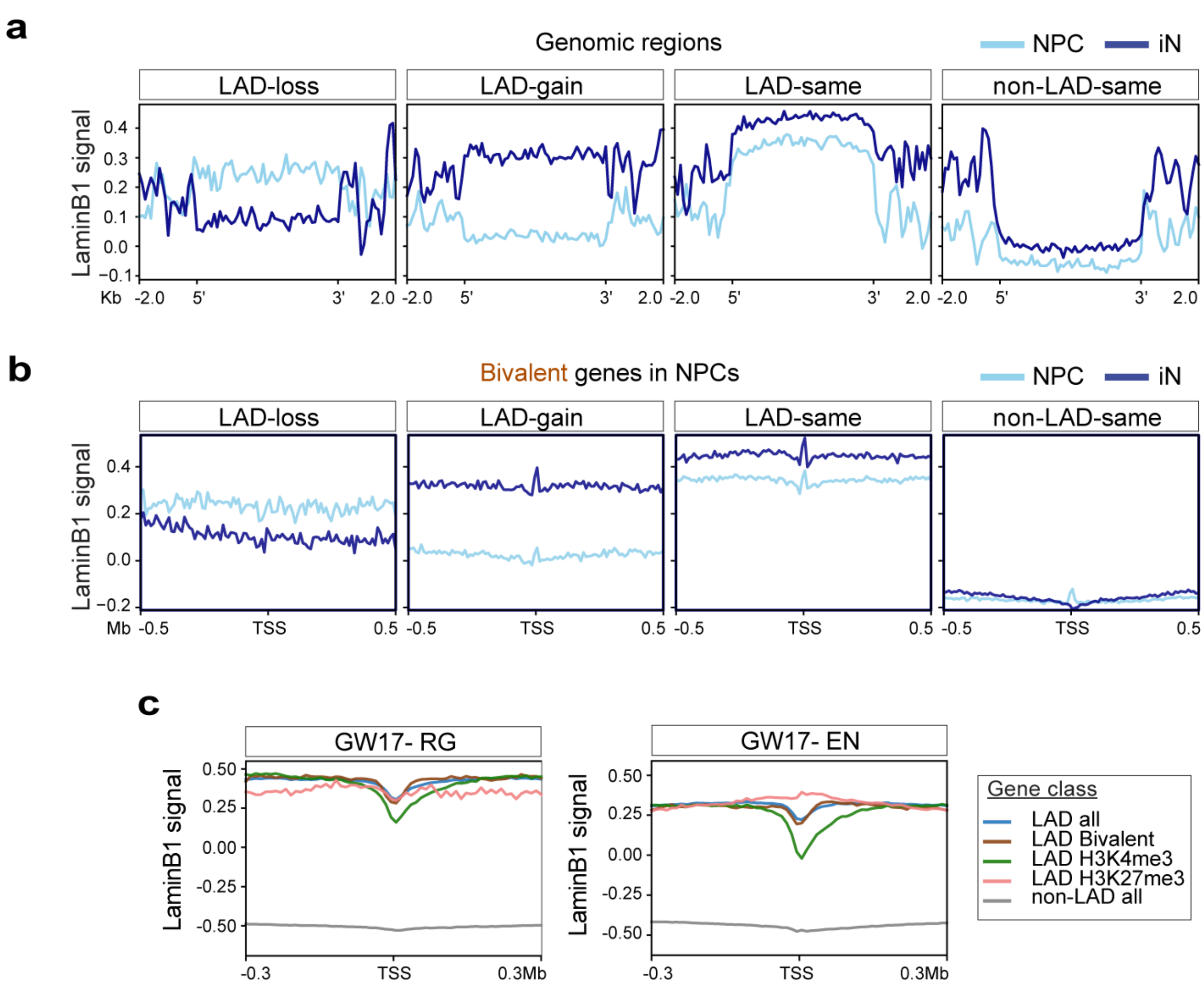

Reviewer Response Figure 20. a, Metagene plots of normalized LaminB1 signal in LAD-loss, LAD-gain and LAD-same regions in NPC and iN. b, Metagene plots of normalized LaminB1 signal in at LAD bivalent genes in NPCs. **c**, Metagene plots of normalized LaminB1 signal at LAD genes stratified by chromatin state in RG and EN. LaminB1 signal for non-LAD genes is shown for comparison.

-The conclusion of the experiments presented in Fig. 7 is that upon H3K27me3 inhibition gene expression of bivalent genes does not change in LADs. However, it does also not change in non-LADs (Fig. 7c). I therefore do not see the significance of the finding and how it contributes conceptually to interrelationship between LADs and bivalent genes.

We appreciate the opportunity to strengthen the conceptual significance of our findings. In our original experiments, we utilized ESC-derived NPCs. While we observed increased gene expression following EZH2 inhibition in these cells, the change did not reach statistical significance due to the limited number of bivalent genes. Nonetheless, it was notable that bivalent genes within LADs remained lowly expressed despite the depletion of H3K27me3. In light of the relatively small number of bivalent genes in ESC-derived NPCs, we repeated the EZH2 inhibition experiment in NPCs derived from the WTC11 iPSC line. Compared to ESC-derived NPCs, these iPSC-derived NPCs exhibit higher levels of H3K27me3 and a correspondingly greater number of bivalent genes. Consistent with our findings in ESC-derived NPCs, EZH2 inhibition in iPSC-derived NPCs led to global loss of H3K27me3 without any detectable changes to LAD architecture. Although the expression of bivalent genes in both LADs and non-LADs was significantly de-repressed upon EZH2 inhibition, bivalent genes located within LADs remained significantly more repressed than those outside LADs. These results support the conclusion that lamina association plays a primary role in maintaining the repression of bivalent genes independent of H3K27me3. These findings are described in the revised manuscript in the section "The lowly expressed state of lamina-associated bivalent genes is independent of H3K27me3" and shown in **Fig. 5a-d and ED Fig. 10a-b**.

In addition to uncovering a more robust phenotype of EZH2 inhibition in NPCs, we sought to further strengthen the claim that the environment of the lamina is sufficient for transcriptional repression. In order to directly test whether lamina association is sufficient to repress transcription, we developed a LacI-LacO tethering system to target plasmid DNA reporters to the nuclear periphery. This system includes (1) a LacI-GFP or LacI-GFP-LaminA fusion protein, and (2) a 256-copy LacO array-containing plasmid encoding RFP under the *Ef1a* promoter. Upon co-transfection, tethering the pLacO-RFP construct to the nuclear lamina via LacI-LaminA resulted in robust transcriptional repression of RFP expression. Notably, this repression occurred independently of local H3K27me3 levels: EZH2 inhibition significantly increased reporter expression from untethered plasmids but had no effect on expression of plasmids tethered to the lamina. These data demonstrate that lamina association is sufficient to induce transcriptional repression, independent of local H3K27me3. These results are presented in the revised manuscript under the section "Nuclear lamina-tethered DNA constructs are repressed independent of H3K27me3" and shown in **Fig. 5e-l and ED Fig. 10c-e**. Together, these new experiments strongly support a model in which nuclear lamina association is a primary regulator of the repressed state of LAD genes, acting independently of local H3K27me3.

Referee #1 (Remarks to the Author):

The reviewers provided new data and analyses and have addressed most of my previous concerns. The new data on mechanistic insight is exciting.

We thank the reviewer for this positive assessment of our manuscript. The constructive feedback received from their earlier review helped improve the clarity and depth of the story.

Referee #2 (Remarks to the Author):

The authors have carefully addressed most of my earlier comments, and the study overall provides interesting insights into spatial genome organization. Nevertheless, the data supporting Fig. 6 are too limited in quality to substantiate the conclusions drawn, and I would recommend reconsidering their inclusion in the manuscript. Furthermore, while the work is of clear relevance to the field, I do not believe the findings reach the level of broad conceptual advance expected for Nature more for a specialized journal like Nature Neuroscience.

We thank the reviewer for their careful re-evaluation of our manuscript. In response to concerns regarding Fig. 6, we have substantially expanded and strengthened the underlying analyses through new biological replicates, additional CUT&RUN experiments, extended quantitative analyses, and integration of independent nascent transcriptomic datasets. These additions significantly reinforce the robustness of the findings and our mechanistic conclusions. We believe the revised manuscript provides broadly relevant conceptual insights into how subnuclear genome organization regulates transcription, local chromatin states and developmental gene programs.

The main revisions are summarized below:

- **Additional Pol II Profiling:** We performed CUT&RUN profiling of total Pol II, Ser5P, and Ser2P in additional RG replicates and iPSC-derived NPCs, strengthening evidence for restricted transcriptional elongation at the nuclear lamina (**revised Fig. 6a, Extended Data Fig. 10f**).
- **Integration of GRO-seq:** We integrated published GRO-seq data from iPSC-derived NPCs, providing independent functional evidence for reduced transcriptional elongation at LAD-associated bivalent genes (**revised Fig. 6b**).
- **Extended quantitative imaging analyses:** We expanded quantitative imaging analyses of all subunits of the NELF complex (NELF-A, NELF-B, NELF-CD and NELF-E) and elongation factors with additional replicates (and ≥50 cells per condition), confirming the depletion of elongation factors and relative enrichment of the NELF complex at the lamina (**revised Fig. 6d-f)**.
- **Genomic mapping of the NELF Complex:** We successfully generated genomic profiles of NELF-A and NELF-E in RG and NPCs, demonstrating their presence at LAD gene promoters, including lamina-associated bivalent genes (**revised Fig. 6g, Extended Data Fig. 10j**).

Point-by-point response:

1. I find the conclusion that RG LAD-associated genes show reduced Pol II-Ser2 occupancy relative to non-LAD bivalent genes, whereas Pol II-Ser5 does not, insufficiently supported by the

data in Fig. 6a. In fact, Pol II-Ser5 signal appears somewhat higher in LADs compared to non-LAD bivalent domains. The observed differences are not particularly convincing and may primarily reflect differences in baseline signal rather than meaningful biological variation.

We acknowledge the reviewer's concern that the differences in Pol II occupancy between LAD and non-LAD bivalent genes in the previous Fig. 6a appeared modest, which may have been due to low cell input. To address this, we performed new CUT&RUN experiments for total Pol II, Pol II-Ser5P, and Pol II-Ser2P in RG using increased input material. These experiments improved the signal-to-noise ratio and reveal clearer differences in Pol II-Ser2P occupancy between LAD and non-LAD bivalent genes. While total Pol II and Pol II Ser5P were enriched at bivalent promoters regardless of their LAD status, Pol II-Ser2P was markedly depleted from the gene bodies of LAD-associated bivalent genes in RG (**revised Fig. 6a; Extended Data Fig. 10f**; **see also response to reviewer comment # 4**). Part of this data is shown below in **reviewer Fig. 1a.**

To further strengthen our conclusions, we performed parallel CUT&RUN analyses for total Pol II, Ser5P, and Ser2P in iPSC-derived NPCs. These data also showed pronounced reductions in elongating Pol II-Ser2P at LAD-associated bivalent genes relative to non-LAD bivalent genes, compared to modest differences in total Pol II and Pol II-Ser5P (**revised Fig. 6a; also shown below in reviewer Fig. 1b**).

To directly assess the transcriptional activity of Pol II, we analyzed published nascent transcription data (GRO-seq) from iPSC-derived NPCs. This independent dataset demonstrated that LAD-associated bivalent genes exhibit significantly reduced gene-body transcription (**revised Fig. 6b and shown below in reviewer Fig. 1c)**, providing additional evidence that transcriptional elongation is restricted at the lamina.

Together, these results demonstrate that proximity to the nuclear lamina restricts transcriptional elongation of bivalent genes despite promoter-proximal Pol II recruitment.

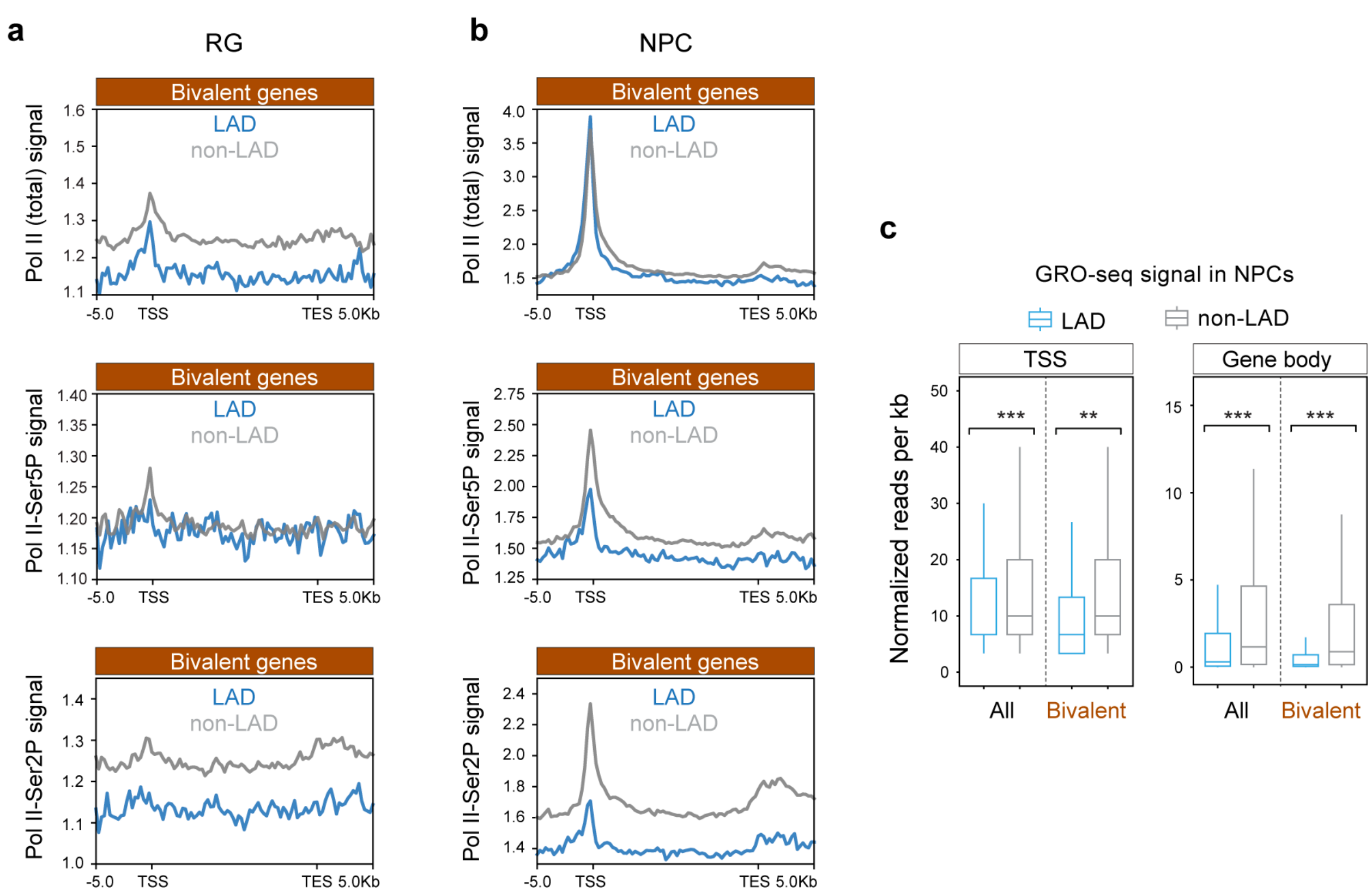

Reviewer Figure 1. (**a-b)** Metagene profiles showing CUT&RUN signal for total Pol II, Pol II-Ser5P, and Pol II-Ser2P at bivalent genes in **(a)** RG and **(b)** iPSC-derived NPCs. IgG normalized signals are plotted across the gene body from the transcription start site (TSS) to the transcription end site (TES) including 5 kb upstream and downstream flanking regions. **c,** Box plots showing nascent transcription levels (GRO-seq signal) at the TSS and gene body for LAD and non-LAD genes in iPSC-derived NPCs.

2. Expanding the analysis window to ±10 kb upstream and downstream of the gene body might help clarify whether the apparent trends persist beyond the immediate gene region, especially given the current signal appears quite noisy.

As suggested, we have expanded the analysis window to ±10 kb flanking regions (**reviewer Fig. 2a**), which are presented alongside the ±5 kb window for comparison (**reviewer Fig. 2b**). This broader window does not change the overall occupancy patterns of Pol II. We have updated the figures in the revised manuscript to use a ±5 kb flanking window (extended from the original ±2 kb) to provide a clearer view of these occupancy patterns (**revised Fig. 6a, c, g; Extended Data Fig. 10 f, g, j**).

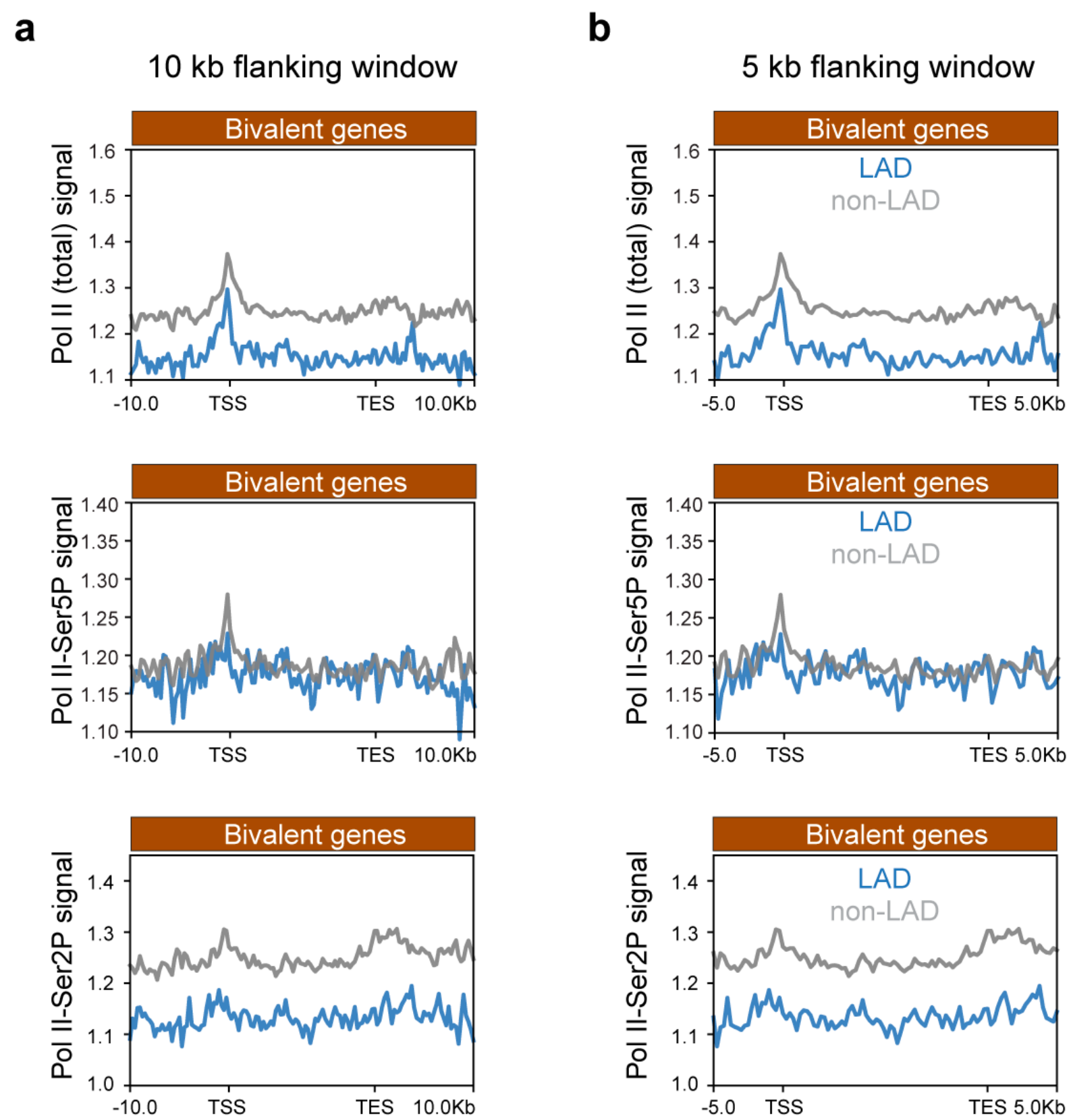

Reviewer Figure 2. Metagene profiles showing total Pol II, Pol II-Ser5P, and Pol II-Ser2P occupancy at bivalent genes located in LADs and non-LADs. IgG normalized signals are plotted across the gene body from the TSS to the TES. Profiles are shown for a **(a)** ±10 kb flanking window and **(b)** ±5 kb flanking window.

3. Additionally, I am puzzled by the difference in both pattern and baseline levels of Pol II-Ser2 and Pol II-Ser5 in RG samples for LAD-associated genes when comparing Fig. 6a and Fig. 6b. While I understand that the gene selections differ slightly between panels, such discrepancies raise concerns about the consistency of the signal and reduce confidence in the robustness of the conclusions.

The apparent differences in Pol II-Ser2P and Pol II-Ser5P patterns between Fig. 6a and Fig. 6b reflect the distinct biological gene sets analyzed in these panels rather than technical inconsistency. Specifically, Fig. 6a examines all bivalent genes stratified by LAD association, representing a broader gene set. Fig. 6b focuses on a more restricted subset of bivalent genes located in LAD-loss regions that resolve to H3K4me3 monovalent state during RG to EN differentiation. As a result, differences in baseline signal and pattern between the two panels primarily reflect differences in gene set composition.

Of note, during the revision process we realized that we had accidentally generated these metagene plots without regard to strandedness. Thus, when computing the metagene average, genes on the "-" strand were processed "backwards" with their TSS shown as the TES in the plot. This may have contributed in part to low apparent signal-to-noise ratios at the TSS. We have corrected this error in the revised figure, which now accounts for strandedness (**revised Fig. 6c; also shown below in reviewer Fig. 3**).

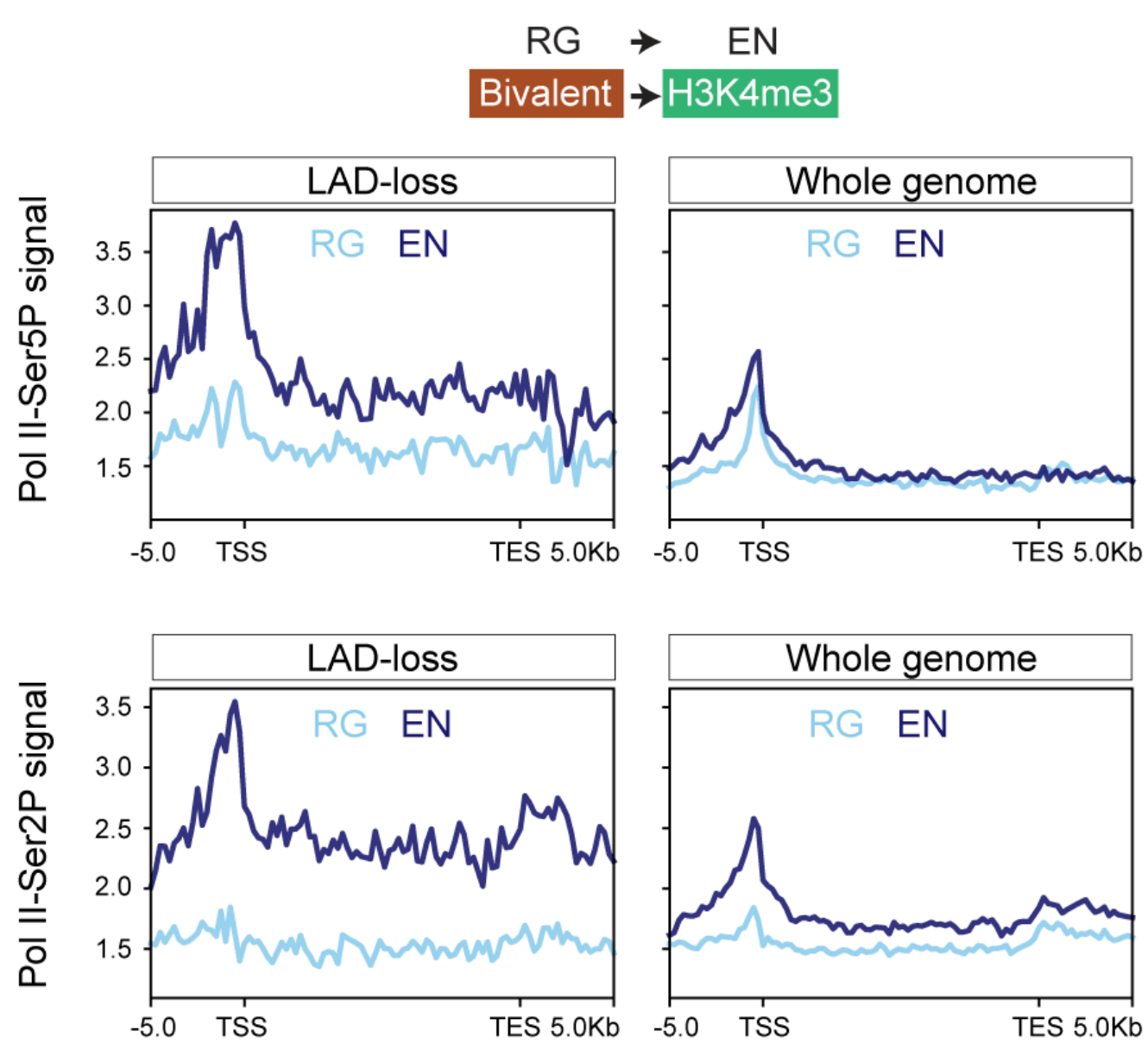

Reviewer Figure 3. Metagene profiles showing Pol II-Ser5P and Pol II-Ser2P occupancy patterns in RG and EN. Signals are plotted across the gene body from the TSS to the TES, including 5.0 kb flanking regions. Data are shown for a subset of genes that are bivalent in RG and resolve to H3K4me3 monovalent state in EN in LAD-loss regions compared to the whole genome.

4. To better assess the quality of this dataset, it would be useful to perform a similar analysis across all active and inactive genes. This would help establish the expected differences in Pol II levels and clarify the baseline signal levels.

As it stands, the data quality appears insufficient to robustly support the key conclusions drawn in relation to Fig. 6.

We thank the reviewer for suggesting these analyses. We examined total Pol II, Pol II-Ser5P, and Pol II-Ser2P signals across active (TPM > 1) and inactive (TPM < 0.1) genes in RG to establish an expected dynamic range of Pol II signal. While both LAD and non-LAD bivalent genes exhibited recruitment of total Pol II and initiating Pol II-Ser5P at the TSS, LAD-associated bivalent genes showed a strong reduction in elongating Pol II-Ser2P signal throughout the gene body (**reviewer Fig. 4)**. This Ser2P signal in LAD bivalent genes (blue) remained near the level

of inactive genes (red), whereas non-LAD bivalent genes (grey) showed intermediate occupancy between active and inactive states. These data indicate that the nuclear lamina environment restricts the transition to transcriptional elongation at bivalent genes, rather than preventing initial Pol II recruitment. This analysis supports the technical quality of the dataset and strengthens confidence in the robustness of the conclusions drawn in revised Fig. 6.

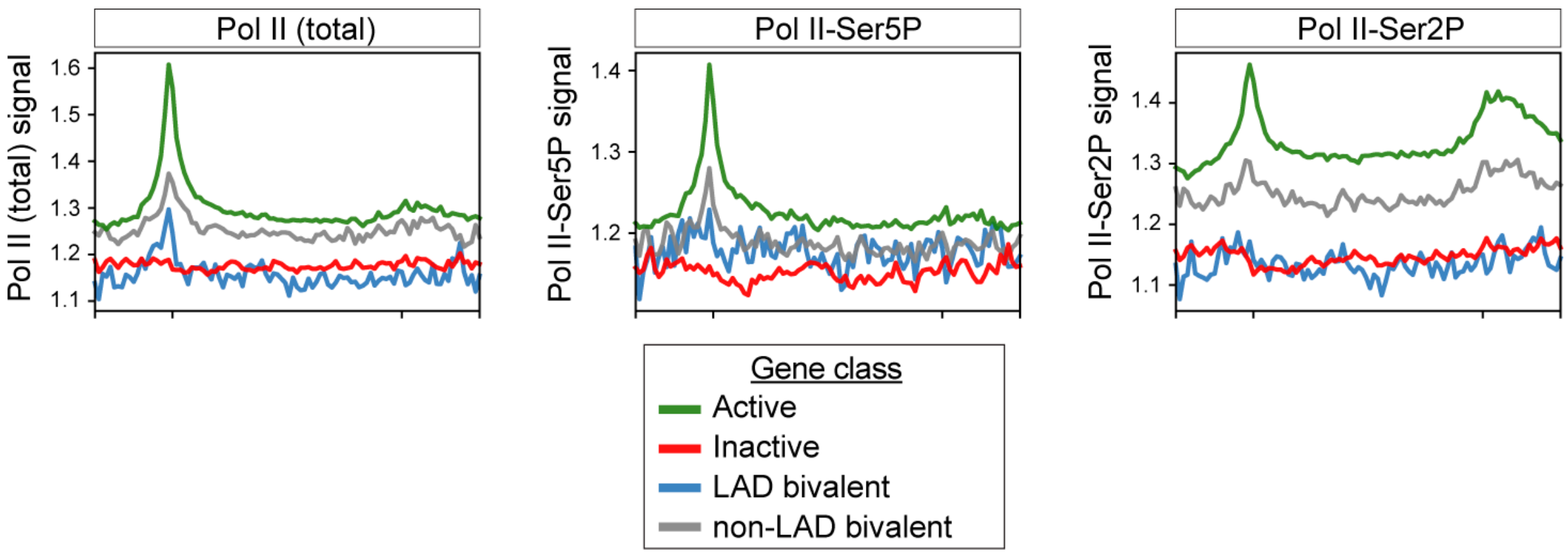

Reviewer Figure 4. Metagene profiles showing total Pol II, Pol II-Ser5P, and Pol II-Ser2P signal across different gene classes in RG. Genes are categorized as **active** (TPM > 1), **inactive** (TPM < 0.1), **LAD bivalent** and **non-LAD bivalent**.

5. The results shown in Fig. 6c–d are promising and raise interesting hypotheses. Nonetheless, the current analysis appears to be based on single experimental replication and low cell numbers, which weakens the robustness of the conclusions. In particular, the findings related to NELF enrichment are potentially significant but would benefit from supporting genomic evidence to confirm its specific localization at the nuclear periphery and association with bivalent genes.

We thank the reviewer for these constructive comments, which have helped strengthen the conclusions presented in previous Fig. 6c–d. In response to the concern regarding replication and cell numbers, we performed additional immunostaining experiments for NELF, and elongation factors using independent biological replicates and expanded our quantitative analyses to ≥50 cells per condition. Furthermore, beyond NELF-B, we extended our analysis to additional components of the NELF complex, namely NELF-A, NELF-C/D, and NELF-E. Notably, all examined NELF subunits show significant enrichment at the nuclear lamina compared to elongation factors (**revised Fig. 6d-f;** also shown below in **reviewer Fig. 5a-b**), reinforcing the robustness of our imaging-based findings.

To provide complementary genomic evidence, we performed CUT&RUN analyses for NELF complex in both RG and iPSC-derived NPCs. Of note, CUT&RUN for NELF has not been previously reported, so we spent considerable effort optimizing this experiment, which is performed on rare, difficult-to-obtain cell populations (primary RG cells purified from human prenatal brain). Although CUT&RUN experiments using antibodies against NELF-B and NELF-C/D were unsuccessful despite multiple attempts, we obtained high-quality datasets for NELF-A and NELF-E for RG and NPC. These data demonstrate that in both cell types, NELF-A and NELF-E engage with promoters of LAD genes including bivalent genes (**revised Fig. 6g, Extended Data Fig. 10j;** also shown below in **reviewer Fig. 5c**). This genomic analysis provides orthogonal evidence consistent with our imaging-based observations of NELF at the nuclear lamina.

Together, these additional imaging and genomic analyses substantially strengthen the robustness of Fig. 6 and support the conclusion that transcriptional elongation is restricted at lamina-associated bivalent genes.

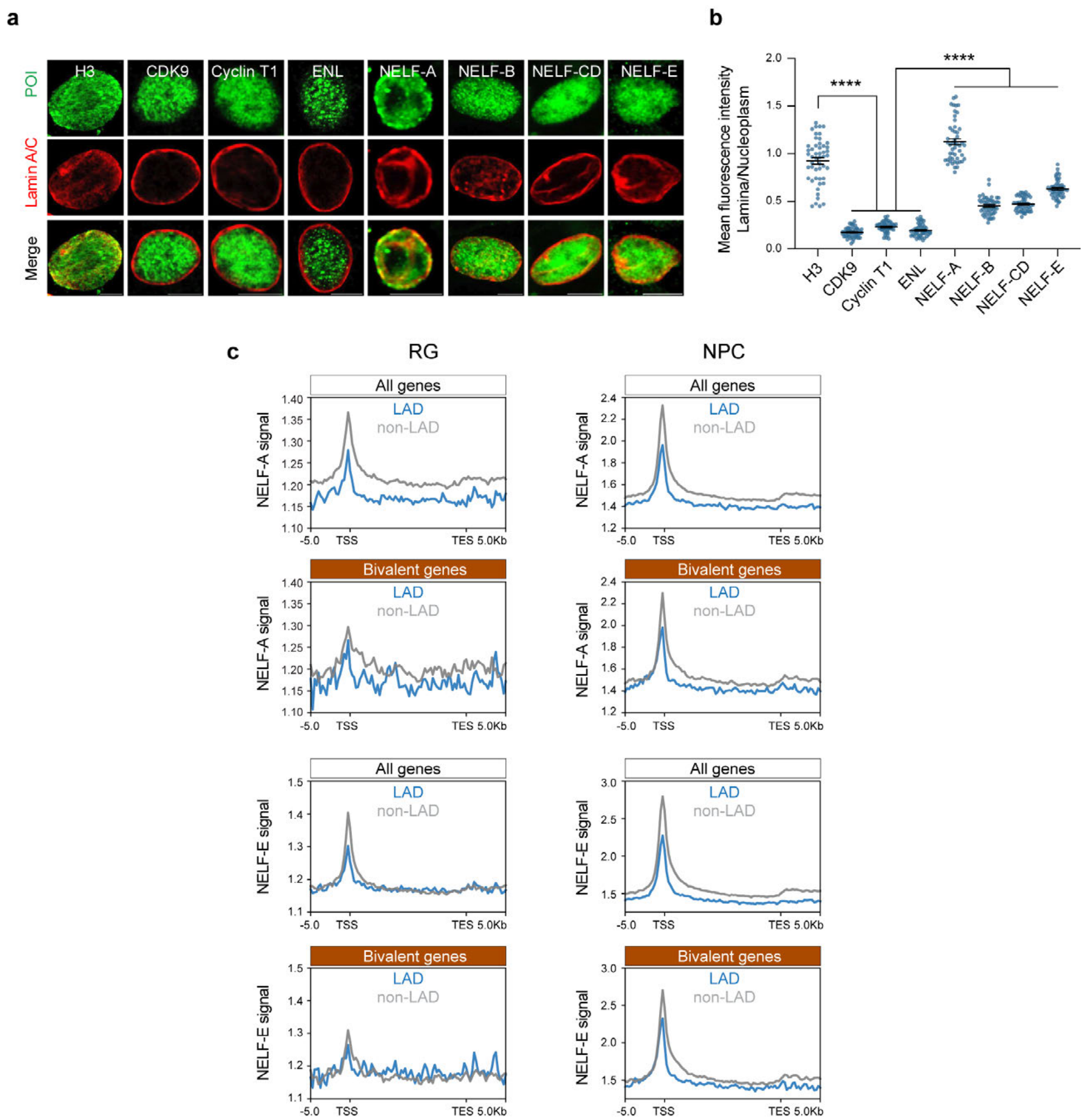

Reviewer Figure 5. (a) Representative immunofluorescence images of nuclei showing the distribution of the indicated proteins of interest (POI) relative to the nuclear lamina. **(b)** Quantification of mean fluorescence intensity ratios (Lamina/Nucleoplasm) for the indicated proteins. **(c)** Metagene profiles showing occupancy of NELF-A and NELF-E in RG and NPCs. Signals are plotted across the gene body (TSS to TES) including 5.0 kb flanking regions for all genes and bivalent genes.
